# metaGOflow: a workflow for the analysis of marine Genomic Observatories shotgun metagenomics data

**DOI:** 10.1093/gigascience/giad078

**Published:** 2023-10-18

**Authors:** Haris Zafeiropoulos, Martin Beracochea, Stelios Ninidakis, Katrina Exter, Antonis Potirakis, Gianluca De Moro, Lorna Richardson, Erwan Corre, João Machado, Evangelos Pafilis, Georgios Kotoulas, Ioulia Santi, Robert D Finn, Cymon J Cox, Christina Pavloudi

**Affiliations:** Institute of Marine Biology, Biotechnology and Aquaculture (IMBBC), Hellenic Centre for Marine Research (HCMR), Former U.S. Base of Gournes, 71003 Heraklion, Crete, Greece; KU Leuven, Department of Microbiology, Immunology and Transplantation, Rega Institute for Medical Research, Laboratory of Molecular Bacteriology, 3000 Leuven, Belgium; European Molecular Biology Laboratory, European Bioinformatics Institute (EMBL-EBI), Wellcome Genome Campus, Hinxton, Cambridge CB10 1SD, UK; Institute of Marine Biology, Biotechnology and Aquaculture (IMBBC), Hellenic Centre for Marine Research (HCMR), Former U.S. Base of Gournes, 71003 Heraklion, Crete, Greece; Flanders Marine Institute (VLIZ), 8400 Oostende, Belgium; Institute of Marine Biology, Biotechnology and Aquaculture (IMBBC), Hellenic Centre for Marine Research (HCMR), Former U.S. Base of Gournes, 71003 Heraklion, Crete, Greece; Centro de Ciências do Mar (CCMAR), Universidade do Algarve, Campus de Gambelas, 8005-139 Faro, Portugal; European Molecular Biology Laboratory, European Bioinformatics Institute (EMBL-EBI), Wellcome Genome Campus, Hinxton, Cambridge CB10 1SD, UK; CNRS, FR 2424, ABiMS Platform, Station Biologique de Roscoff (SBR), 29680 Roscoff, France; Centro de Ciências do Mar (CCMAR), Universidade do Algarve, Campus de Gambelas, 8005-139 Faro, Portugal; Institute of Marine Biology, Biotechnology and Aquaculture (IMBBC), Hellenic Centre for Marine Research (HCMR), Former U.S. Base of Gournes, 71003 Heraklion, Crete, Greece; Institute of Marine Biology, Biotechnology and Aquaculture (IMBBC), Hellenic Centre for Marine Research (HCMR), Former U.S. Base of Gournes, 71003 Heraklion, Crete, Greece; Institute of Marine Biology, Biotechnology and Aquaculture (IMBBC), Hellenic Centre for Marine Research (HCMR), Former U.S. Base of Gournes, 71003 Heraklion, Crete, Greece; European Marine Biological Resource Centre (EMBRC-ERIC), 75005 Paris, France; European Molecular Biology Laboratory, European Bioinformatics Institute (EMBL-EBI), Wellcome Genome Campus, Hinxton, Cambridge CB10 1SD, UK; Centro de Ciências do Mar (CCMAR), Universidade do Algarve, Campus de Gambelas, 8005-139 Faro, Portugal; Institute of Marine Biology, Biotechnology and Aquaculture (IMBBC), Hellenic Centre for Marine Research (HCMR), Former U.S. Base of Gournes, 71003 Heraklion, Crete, Greece; Department of Biological Sciences, The George Washington University, 20052 Washington, DC, USA

**Keywords:** shotgun metagenomics, MGnify, Common Workflow Language (CWL), containers, provenance, RO-Crate

## Abstract

**Background:**

Genomic Observatories (GOs) are sites of long-term scientific study that undertake regular assessments of the genomic biodiversity. The European Marine Omics Biodiversity Observation Network (EMO BON) is a network of GOs that conduct regular biological community samplings to generate environmental and metagenomic data of microbial communities from designated marine stations around Europe. The development of an effective workflow is essential for the analysis of the EMO BON metagenomic data in a timely and reproducible manner.

**Findings:**

Based on the established MGnify resource, we developed metaGOflow. metaGOflow supports the fast inference of taxonomic profiles from GO-derived data based on ribosomal RNA genes and their functional annotation using the raw reads. Thanks to the Research Object Crate packaging, relevant metadata about the sample under study, and the details of the bioinformatics analysis it has been subjected to, are inherited to the data product while its modular implementation allows running the workflow partially. The analysis of 2 EMO BON samples and 1 Tara Oceans sample was performed as a use case.

**Conclusions:**

metaGOflow is an efficient and robust workflow that scales to the needs of projects producing big metagenomic data such as EMO BON. It highlights how containerization technologies along with modern workflow languages and metadata package approaches can support the needs of researchers when dealing with ever-increasing volumes of biological data. Despite being initially oriented to address the needs of EMO BON, metaGOflow is a flexible and easy-to-use workflow that can be broadly used for one-sample-at-a-time analysis of shotgun metagenomics data.

## Introduction

It is well established that microbial assemblages support multiple ecosystem services and that microbial community profiling using metagenomics methods can help elucidate the mechanisms that govern the structure of these communities and their interactions with the environment [1]. The community composition and structure of the marine microbiome is directly correlated with environmental quality [2, 3]. Indeed, the quality of a marine microbial environment (e.g., a marine sediment) can impact the food chain [4] through the physical and chemical effects of secondary metabolites [5]. In addition, secondary metabolites produced by microorganisms may also become targets for bio-prospecting in medicine and industry [6]. Monitoring the changes in microbial community composition and function due to climate change–related stressors, such as ocean acidification or increases in temperature and UV absorption, can provide insights on ecosystem function, health, and resilience [7].

Pioneering research programs, such as the Ocean Sampling Day [8], Malaspina circumnavigation expedition [9], and Tara Oceans [10], have been instrumental in collecting large series of marine genomic samples from sites around the globe. The analysis of data resulting from these studies has greatly increased our understanding of the importance, the role, and the mechanisms governing microbial communities in some of the most common, sensitive, or threatened marine environments [11–13]. European Marine Omics Biodiversity Observation Network (EMO BON) [14], a European Marine Biological Resource Centre (EMBRC-ERIC) [15] initiative, is designed to continue and expand this effort by regular bimonthly microbial genomic biodiversity samplings at designated marine coastal stations around the European coastline. In the first 2 years of the EMO BON (2021–2022), it is expected that more than 540 shotgun metagenomic data sets from water column and sediment samples will be generated from 17 European sites.

The ultimate success of Genomic Observatories (GOs) depends on the development and adoption of standards for sampling, metadata collection, sequencing, and data analysis. The provision of metadata relating to the raw sequence data, data products, and their analysis methods is of high importance for interpretation and interoperability, and it needs to be accessible in both human- and machine-readable formats. Legislative frameworks, such as the Nagoya Protocol for Access and Benefit Sharing [16], and community written frameworks, such as those developed by the Genomic Standards Consortium [17, 18], as well as initiatives encouraging adherence to best practices, such as the Better Biomolecular Ocean Practices project [19, 20], have all been key to providing an agreed-upon standard that aims to fulfill these needs. Standard operating procedures and standardized methods of analysis enable the comparison of results among sites, through time, and among projects, without which much of the value of the data for environmental assessment is lost.

Effective analysis of shotgun metagenomic data is time-consuming, especially regarding computational steps such as sequence assembly and annotation [21]. Moreover, microbial community profiling and functional analyses are most useful when samples are maximally comparable in space and time, and they have been thereby treated using the same analytical procedures. To address the challenges that arise when analyzing metagenomic data, numerous workflows and pipelines have been developed. Notable pipelines include metaWRAP [22], bioBakery [23], and nf-core [24], which provide a collection of pipelines such as nf-core/ampliseq [25] and nf-core/taxprofiler [24]. Recently, containerization approaches (e.g., Docker [26], Singularity [27]), along with workflow managers (e.g., Nextflow [28], Snakemake [29]), have been widely used to (i) address the complexity of the analysis, (ii) facilitate execution and reproducibility, and (iii) distribute and share software to a broader audience [30]. nf-core and ATLAS [31] shotgun metagenomic analysis pipelines are examples of the implementation of such approaches.

Additionally, there are (data analysis) resources like MG-RAST [32], MGnify [33], and IMG/M [34] that come with their own distinct advantages and disadvantages.

The computing requirements for the analysis of the EMO BON data may exceed the computing capacity that a single research institute and/or regional high-performance computing (HPC) (i.e., tier 2) systems can support using the available workflows. Indicatively, for a single dataset, software tools related to the retrieval of taxonomic profiles require up to 160 CPU hours and up to 100 GB of RAM [35]. Computing requirements for the functional annotation of shotgun reads are even higher. Nevertheless, timely provision of data and data products from GOs is of paramount importance to facilitate long-term ecological studies, to accelerate policy-making, and to directly assess the impact of anthropogenic effects on the marine environment.

**Figure 1:**
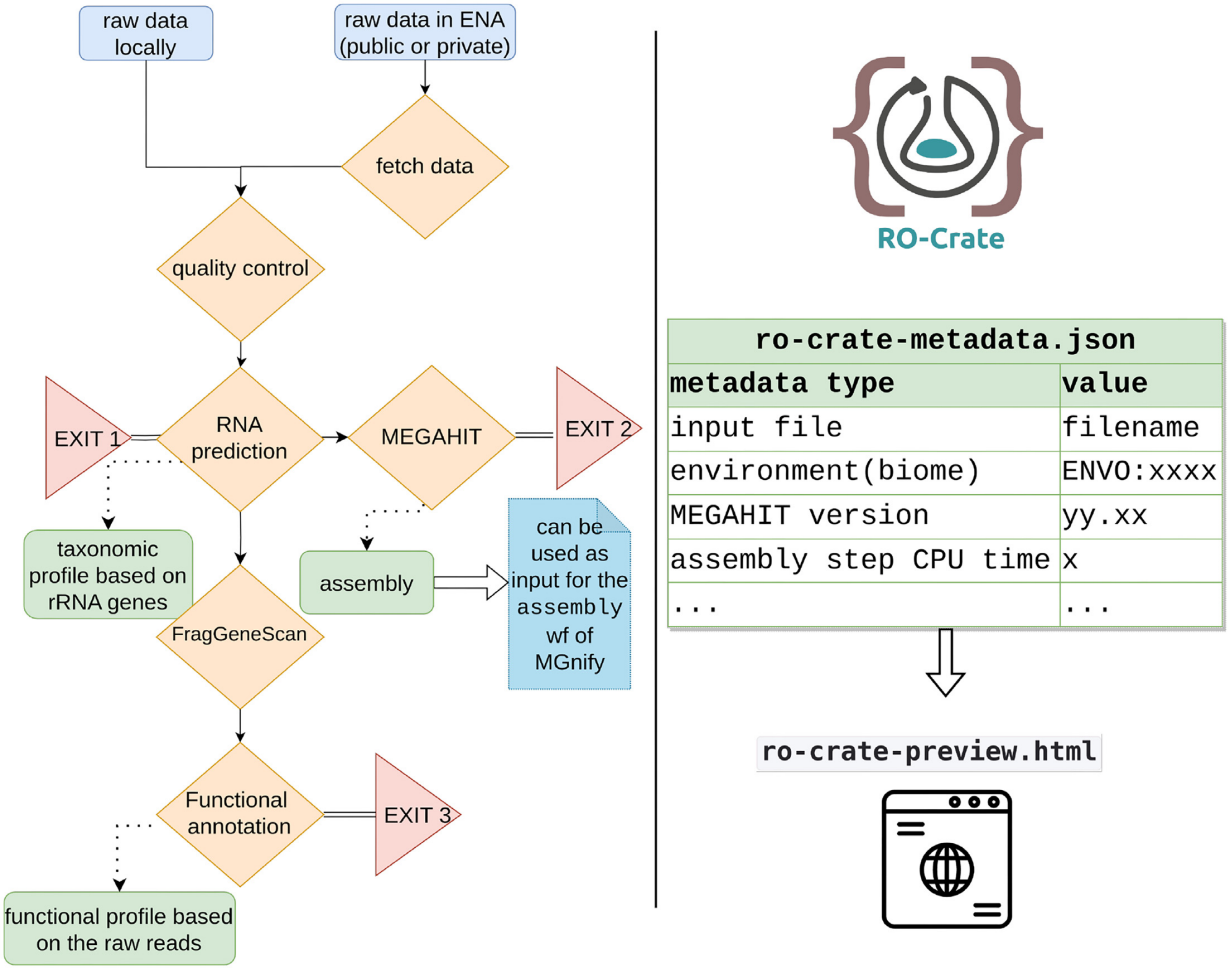
Schematic overview of metaGOflow, showing the main steps of the analysis along with their corresponding data products; the partial execution of the workflow is also shown by the potential exit points (left). Independent of the steps to be performed, once completed, an RO-Crate is built (right).

To address the challenges of analyzing GO data in a timely and standardized framework, we developed metaGOflow: a MGnify-based [33] computational workflow that implements the critical steps of a shotgun metagenomic bioinformatics analysis and provides rich provenance metadata describing the data, data products, and workflow execution (Fig. 1). The novel aspects of this workflow are mainly (i) partial workflow execution (e.g., the user has the flexibility to choose whether to run the functional annotation subworkflow or not, or even run it at a later point using the data products of the previous steps), (ii) the incorporation of an alternative assembler with a significantly lower computational cost as compared to the MGnfiy default one, and (iii) the ultimate generation and verification of a Research Object (RO) crate ensuring the workflow’s FAIRness. On top of that, several updates of the databases and tools invoked by MGnify have been performed.

metaGOflow consists of two basic concepts:

- an *analytical workflow* that provides taxonomic inventories and community gene function profiles of the samples as data products packaged in RO-Crates [36],
- a *data provenance workflow* that generates extensive metadata and thereby provides compliance of the data, data products, and analytical procedures with Findable, Accessible, Interoperable, and Reusable (FAIR) data practices and the principles of Open Science, also packaged in the RO-Crates [19, 37].

## Implementation

### Overview

The pillars around which metaGOflow [SCR_023674] has been built—namely, containerization technologies such as Docker [26] and Singularity [27], as well as the Common Workflow Language (CWL) [39, 40]—ensure the workflow’s ability to perform in different HPC and cloud computing platforms, following the MGnify example.

metaGOflow inherits the architecture of MGnify pipeline-v5 [41] and exploits several of the already containerized tools and the subworkflows implemented in the MGnify pipeline. Several enhancements and upgrades allow metaGOflow to make use of the latest versions of the tools and databases invoked. metaGOflow makes extensive use of CWL *subworkflows* and *conditional* step execution to address the specific needs of the EMO BON project from a computing resources point of view.

For example, the user can run the workflow to only generate the taxonomic inventory of a sample. Then, at a later time and by using the output of the first analysis, the user can also generate the assembly of this sample’s reads and/or their functional annotation. This flexibility in the workflow is essential as there are a considerable number of samples to be analyzed (preferably in as short a period of time as possible), and the computing requirements, especially for the functional annotation step, can be substantial .

In its current version (v.1.0.1[42]), metaGOflow has 5 distinct steps. As in MGnify, metaGOflow analyzes a single sample at a time (see Fig. 1). The user may either provide locally stored raw data (.fastq files) or start the workflow by giving a European Nucleotide Archive (ENA) [43] run accession number. In the later case, metaGOflow invokes the fetch_tool [44] to retrieve the raw sequence files from ENA; if the data to be retrieved are held privately, the username and password of the associated ENA account are also requested. The user sets the steps of the workflow to be performed and provides values for certain tool parameters through a text-based configuration file (config.yml).

To enhance the FAIRness of the data products and of the bioinformatic analysis, metaGOflow data products are packaged as RO-Crates: this allows the set of files to be semantically described, to be accompanied by the metadata that describe the precise steps of the workflow execution, as well as the tools and the parameters used, and to flag the specific input and output files. This description is provided in a JSON-LD file following a particular (user-generated) profile. Along with the data products, the RO-crate contains information describing the version of the workflow per se, including the software and database versions that it uses.

A comparison of the main features of metaGOflow with other commonly used pipelines for shotgun metagenomic analysis is given in Table 1.

**Table 1.** Comparison of the main features and implementation of pipelines similar to metaGOflow

Category	Feature	MetaWRAP	ATLAS	nf-core/taxprofiler	nf-core/funcscan	metaGOflow
	Quality control	fastqc	—	fastp, falco	—	fastp
Preprocessing	Filtering	Trim Galore	BBTools	porechop, fastp, bbduk, prinseq++, Filtlong	—	fastp
	Host-read removal	bmtagger	—	Bowtie2 for short reads and minimap2 for long reads	—	—
	Taxonomy assignment of rRNA genes	—	—	—	—	mOTUs, MAPseq
Taxonomy	Taxonomic assignment of reads and/or contigs	kraken, kraken2	—	Kraken2, DIAMOND, mOTUs, MetaPhlAn3, MALT	—	—
	Taxonomic assignment of bins	TAXATOR-TK	GTDB-tk	—	—	—
	Short-read assembly	metaspades and/or MEGAHIT	MEGAHIT	—	—	MEGAHIT
Assembly	Hybrid assembly	—	Yes	—	—	—
	Groupwise coassembly	Yes	Yes	—	—	—
	Genome binning	metaBAT2, MaxBin2, CONCOCT	metabat2, maxbin2	—	—	—
BINs-MAGs	Bin refinement	Binning-refiner	—	DAS Tool	—	—
	Gene prediction	—	—	prodigal	—	FragGeneScan
Annotation	Functional annotation	prokka (using the bins)	eggNOG	—	hAMRonization, AMP-combi, comBGC.py	InterProScan, eggNOG, hmmsearch
	Ontologies	—	eggNOG	—	—	KEGG, GO, pfam, eggNOG, InterPro
	Keeping track of sample’s metadata	—	—	—	—	Yes
FAIR-ness	Output as RO-Crate	—	—	—	—	Yes
	Workflow provided through containers	—	—	—	Yes	Yes
Architecture	Workflow manager	—	snakemake	nextflow	nextflow	cwl

metaGOflow is available on GitHub [45]. A Continuous Integration/Continuous Deployment workflow using GitHub Actions ensures the validity of the workflow’s cwl main script and, therefore, all its components. A thorough description of how to install and use metaGOflow, as well as common errors that might occur during the analysis of a sample, can be found at its wiki page [46], as well as on its main documentation page [47]. The databases to be installed before using metaGOflow require 160 GB of storage, and as a rule of thumb, the user should allocate 1 TB of storage to perform a metaGOflow analysis.

The development and testing of metaGOflow were performed in the “Zorbas” HPC of the Institute of Marine Biology, Biotechnology and Aquaculture (IMBBC) [30] and at the HPC facility of the Center of Marine Sciences (CCMAR). Further testing was performed on the Luxembourg national supercomputer MeluXina [48]. The use case experiments (see "Use case" section) were performed in a “fat” node of the “Zorbas” HPC (2× Inter Xeon Gold 6230 CPU @ 2.10 GHz 40 cores and 500 GB).

### Step 1: Sequence preprocessing

**Figure 2:**
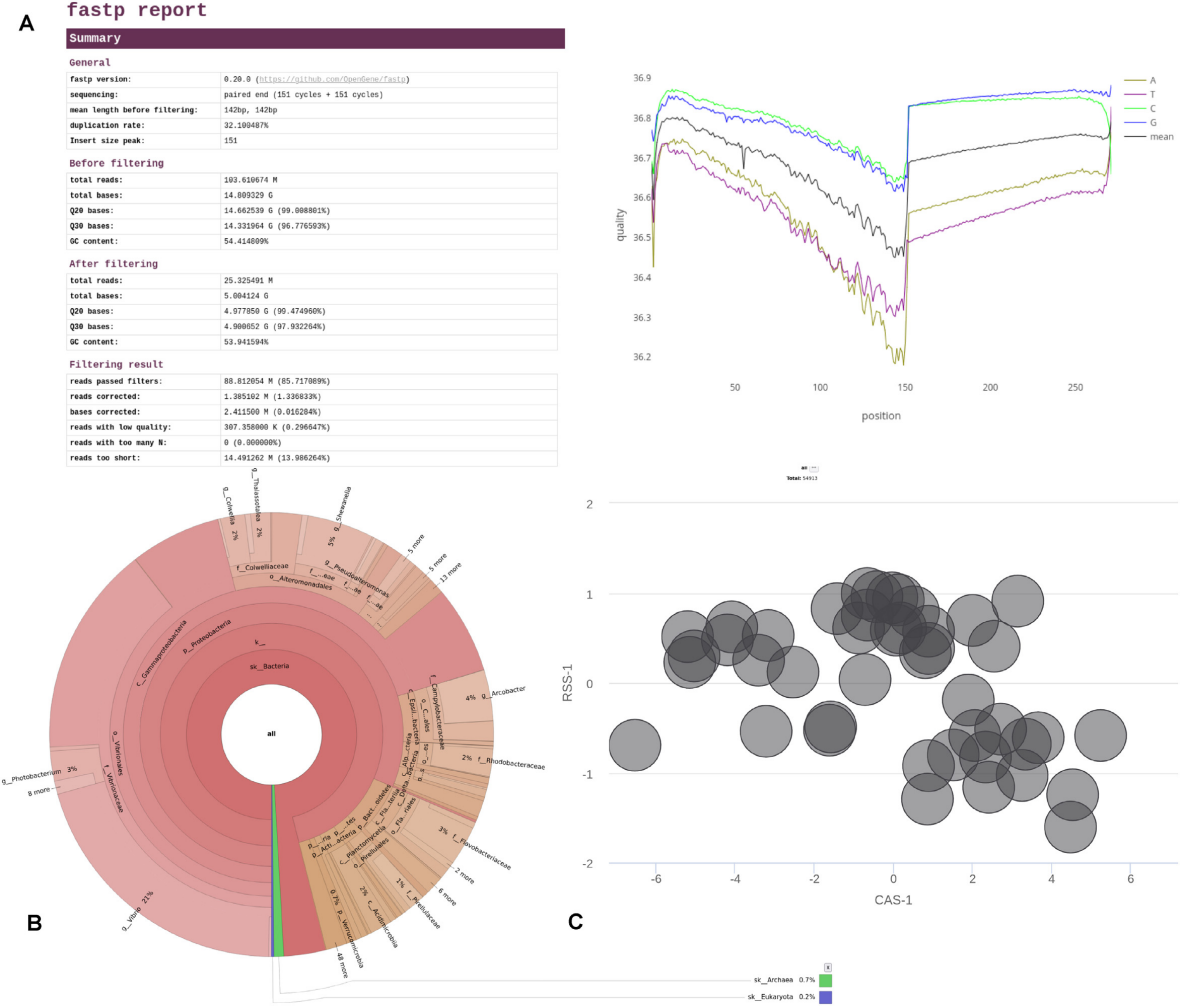
Visualization of metaGOflow’s main output. (A) Raw data are first filtered and only high-quality sequences are analyzed further in the next steps. An .html file with the report of the merged reads is produced. Here, an excerpt of this report is shown: reads’ statistics before and after filtering (left), ATGC chart with the quality of each base cycle after cycle for the merged reads (right). (B) The taxonomy inventory step returns molecular operational taxonomic units (mOTUs) and the taxonomic composition based on the LSU and the SSU genes. Here, the taxonomic composition is represented by a Krona interactive visualization. (C). The functional annotation step returns text files with the GO, KEGG, InterProScan, and Pfam terms retrieved. The retrieved GO terms are presented using Navigo [38], the Co-occurrence Association Score (CAS-1), and the Relevance Semantic Similarity (RSS-1). The Gene prediction step returns a .ffn and a .faa file while the assembly step a .fasta file, including the contigs retrieved. The main output of the provenance feature is the ro-crate-metadata.json file.

Sequences are filtered and merged using fastp (version 0.20.0) [49]. Short, low-quality, and nonmerging sequences are removed and a series of statistical tests describing the quality of the sequencing are performed. An .html file, returned by the fastp tool, provides visualizations of these statistics (see Fig. 2A). The filtered sequences and the merged filtered sequences are returned as .fasta files.

### Step 2: Taxonomy inventory

metaGOflow makes use of the esl-sfetch miniapp of the EASEL library (S. R. Eddy, unpublished data) to index the filtered sequences and support fast sequence retrieval. Then cmsearch, an Infernal program [50, 51], is performed using the ribosomal and the noncoding RNA Rfam covariance models (version v13.0) against the filtered sequences. Eventually, this is followed by taxonomic classification using MAPseq (version 1.2.3) [52] and the SILVA database (version 132) for the taxonomic classification of the small subunit (SSU) and the large subunit (LSU) sequences, while mOTUs2 [53] quantifies both known and unknown taxa on the filtered sequences. metaGOflow automatically returns Krona plots (an interactive visualization approach of hierarchical data as multilayered pie charts [54]) using the taxonomic assignments made for the SSU and LSU genes (see Fig. 2B).

### Step 3: Assembly

Shotgun metagenomic read assembly requires significant computing resources as discussed in Mitchell et al. [33] and in Vollmers et al. [55]. The extent of the computational “burden” depends heavily on the chosen algorithm. To be able to handle the vast amount of data produced by EMO BON in a timely manner, and since we aim more at unravelling biodiversity at the community rather than at the individual (i.e., species) level, metaGOflow makes use of the MEGAHIT algorithm [56]. Longer contigs would be returned if, for example, metaSPAdes [57] was employed, but given metaGOflow’s high-pace data generation and analysis needs, the MEGAHIT algorithm seems a better match.

### Step 4: Gene prediction on the reads

metaGOflow performs gene prediction using FragGeneScan (v1.20) [58] like MGnify. This step is a prerequisite for the functional annotation of the reads (step 5). To partially run this step, the user needs to provide the merged filtered .fasta file, provided by the sequence preprocessing step.

### Step 5: Functional annotation of the reads

metaGOflow focuses on the potential metabolic processes of the whole community rather than the processes of each individual species. Therefore, it performs functional annotation at the reads level. Using InterProScan (v.57-90) [59], metaGOflow annotates the reads with InterPro5 [60], PFam [61], TIGRFAM [62], ProSite patterns and profiles [63], and GO [64] terms. Functional annotations are returned as text files. Both GO and GO Slim (available at geneontology.org) annotations are returned. EggNOG5 [65] annotation is also performed using the eggnog-mapper (v2.1.8) [66]. Last, metaGOflow invokes the HMMER [67] tool along with the KOfam library [68] to get KEGG orthology annotations [69]. This step requires a significant amount of computing time.

To partially run this step, the user needs to provide the merged filtered .fasta file, provided by the sequence preprocessing step (step 1) as well as the output of the gene prediction step (step 4).

For the visualization of each annotation type, there is number of software; indicatively, in Fig. 2C, the Co-occurrence Association Score of the GO terms found in the sample are plotted against their Relevance Semantic Similarity scores, which quantify the frequency of co-occurring GO terms within the gene annotations in the GO Annotation database, as described in Navigo [38].

### Building RO-Crates

**Figure 3:**
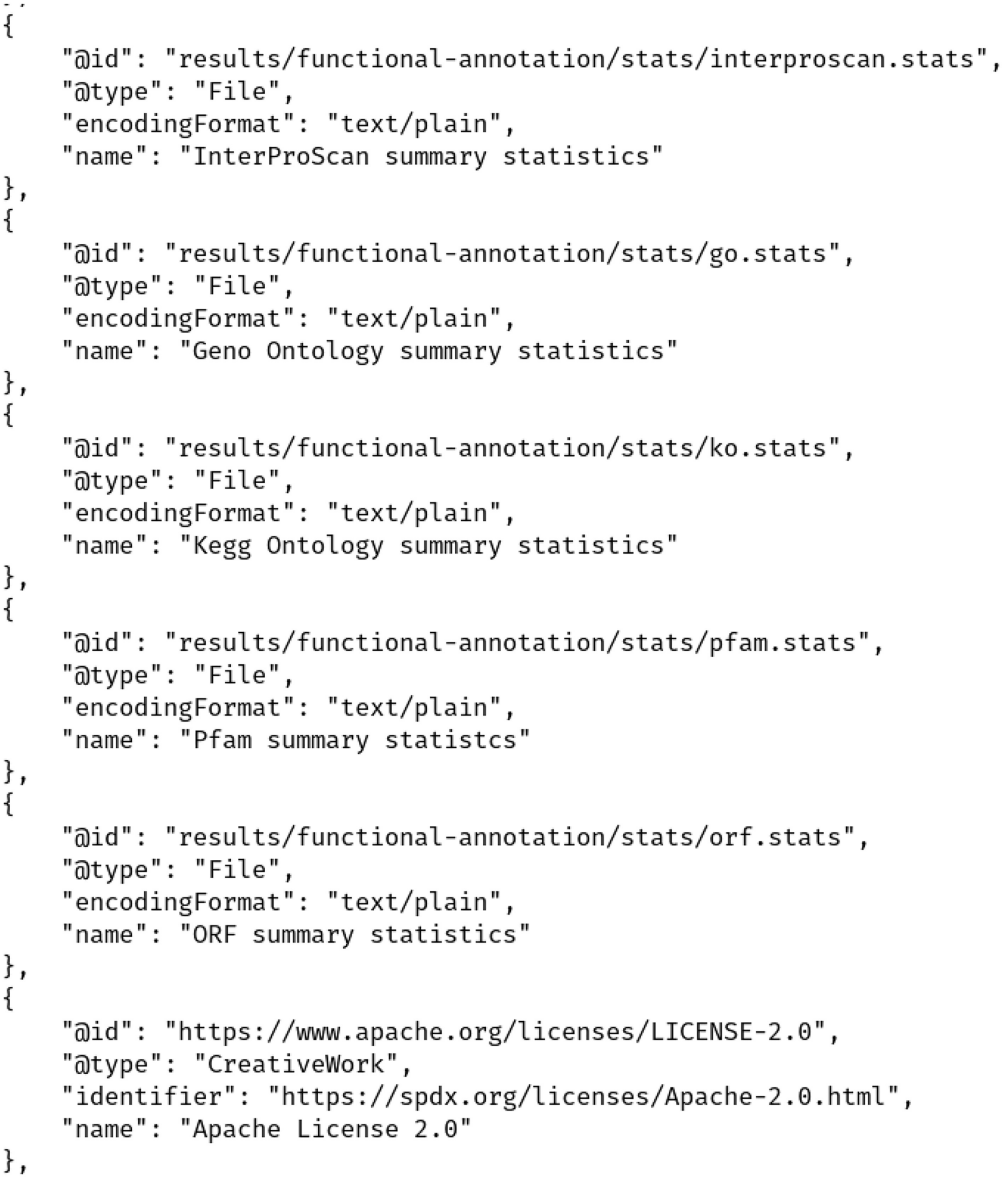
Part of the ro-crate-metadata.json file describing the metaGOflow output files.

An RO-Crate is created automatically by the workflow to store the data products of the aforementioned steps, along with the MetaGOflow run-associated metadata (including the user set parameters, the version, and the source of the workflow used). To this end, the rocrate Python library [70, 71] is used. As mentioned, an RO-Crate object is accompanied by a JSON-LD file (called ro-crate-metadata.json), part of which is shown in Fig. 3, which includes the descriptions of both input and output files.

A thorough list of the metaGOflow’s data products along with their descriptions can be found in the *Description of metaGOflow’s data products* page of the manual. Supporting documentation, related to some of the software tools invoked by metaGOflow, is also provided to support the interpretation of the data products.

### Parameter tuning

The config.yml file is the interface between the user and the pipeline. Through this file, the user sets which steps to perform, a number of parameters related to the idiosyncrasy of each experiment, and parameters that may affect the time efficiency of metaGOflow to a great extent (i.e., number of chunks). Further, metaGOflow supports inline arguments describing technical aspects of how to run (e.g., which containerization technology should be used). A thorough description of these parameters, as well as best practices and rules of thumb, is available at metaGOflow’s manual on the *Arguments and parameters* page.

## Use case

To demonstrate metaGOflow and its key features, the analysis of a sediment and a water column sample from EMO BON was performed. As mentioned in the EMO BON handbook [72] and the EMO BON paper [14], DNA extraction, cleaning, library preparation, and sequencing are performed at a centralized facility to minimize biases and maximize consistency in sequence quality. DNA extraction is performed using commercially available kits, to minimize deviations among samples. The samples were randomly chosen from 2 different stations but are considered representative of EMO BON data. Moreover, an already publicly available marine metagenome sample from the Tara Oceans expedition [73], with size (in Gb) similar to those of the EMO BON data, was also analyzed. All steps of metaGOflow were performed for each of these samples and the computational time (in hours), and the maximum memory (RAM, in GB) is reported in Table 2. Additionally, to demonstrate the applicability of metaGOflow for all types of shotgun metagenomic data, it was implemented for the analysis of a fish gut and a human gut metagenomic sample. All 5 samples were sequenced in different platforms: NovaSeq (EMO BON), HiSeq 2000 (Tara Oceans), BGISEQ-500 (fish gut), and NextSeq 550 (human gut). The metaGOflow results for the gut samples are included in the Zenodo repository [74], and the respective statistics are given in Supplementary Table S1.

**Table 2.** Computing requirements for the analysis of a sediment and a water column EMO BON sample as well as a Tara Oceans water sample, using metaGOflow in a “fat” node of the Zorba HPC

	Computational time (hours)			Memory (max RAM in Gb)		
Workflow step(s)	EB sediment	EB water	TO water	EB sediment	EB water	TO water
Preprocessing and taxonomy inventory (steps 1 and 2)	14.5	12.6	26.4	4.55	4.65	4.15
Assembly (step 3)	1.6	1.22	0.4	8.8	4.38	2.7
Gene calling and functional annotation (steps 4 and 5)	98.7	92.4	84.2	205.1	188.6	155.4

EB: EMO BON; TO: Tara Oceans.

Raw sequences were preprocessed using 130 bp as the minimum length of the reads and at least 30 bp of overlap for the merging step for the 2 EMO BON samples. In case of the Tara Oceans sample, a minimum length of 108 bp was used as the sequences were shorter. The preprocessing and the taxonomic inventory step lasted about from 10 to 24 hours. By allocating a computing node similar to the one used for the use case, taxonomic inventories from at least 300 metagenomes could be produced per year, based on the results from the EMO BON samples.

For the assembly step, a minimum contig length of 200 bp was used for all the samples. The assembly of the reads using the MEGAHIT algorithm was performed in less than 2 hours, while the maximum memory required was less than 10 Gb, which is at least 1 order of magnitude less than what other software (e.g., metaSPAdes) would require. The large number of contigs returned suggests one could aim for a higher minimum contig length. For example, using a minimum contig length of 500 bp for the Tara Oceans sample, the number of contigs was decreased from 102,343 (Table 2) to 34,426, and the required time was about 30 minutes.

The gene calling and the functional annotation steps were those requiring the most computing resources, as expected. For each of the 3 samples, it took about 4 days to complete these steps, with the InterProScan part being the most computationally expensive with respect to both time and memory. In order for metaGOflow to exploit the available computing resources in an optimal way, the user is strongly advised to follow the “Improving performance” instructions of InterProScan and set the relative arguments accordingly.

A summary of the metaGOflow outputs and their respective size for this use case is shown in Table 3. A visual representation of the detailed results (quality control report, taxonomic inventories, functional annotations) of the workflow can be found through this GitHub page [75]. An example of the complete data product of metaGOflow, packed in an RO-Crate, can be found through this Zenodo repo [74]. For the EMO BON samples, the default configuration files config.yml were used; for the Tara Oceans sample, the config.yml is included in the respective RO-crate object, which is available in the Zenodo repository.

**Table 3.** metaGOflow results for the 2 EMO BON samples (marine sediment and a water column) and the Tara Oceans (seawater) sample

Product	EMO BON sediment	EMO BON water	Tara Oceans water
Total reads (M)	51.8	44.0	36.5
Filtered reads (M)	33.2	28.2	19.9
SSU	438	361	345
LSU	719	469	444
Contigs	348,405	338,467	102,343
Reads with predicted CDS (M)	32.4	27.4	18.8
Predicted CDS with IPS match (M)	9.9	9.4	5.2
Predicted CDS with GO match (M)	5.4	5.6	3.2
Predicted CDS with Pfam match (M)	9.3	8.9	4.9
Predicted CDS with KO match (M)	1.0	1.15	0.5

CDS, coding sequences; M, millions.

Based on the scientific questions to be addressed, several types of downstream statistical analysis using the metaGOflow data products might be performed. Most of these statistical approaches are not specific for the analysis of metagenomic datasets per se [76]. On the contrary, they are well established in several research communities: microbial ecologists, microbiologists, and medical scientists. However, the natur–e of the metagenomic data led to several challenges, such as the “compositional effect” that needs to be dealt to the best possible extent [77, 78].

## Discussion and conclusions

Metagenomic applications include different procedures and require expertise in different topics, from field sampling, to lab analyses, to sequencing [79]. This inevitably leads to delays in raw data production, let alone usable scientific results. On top of that, metagenomic raw data are not directly usable as they require time-consuming and computationally demanding processing as well as specialized bioinformatics expertise [76, 79]. For EMO BON and other GOs to produce applicable and fit-for-purpose data, it is of huge importance that quality-controlled and standardized data, as well as informative data products, are made rapidly available. The disentanglement of the analyses from technical expertise and extensive computing infrastructures will allow the direct generation of meaningful data products, even by nonexperts. There is a paramount added value to the provision of preliminary results and data products (i.e,. taxonomic inventories) from metagenomic GO samples as it can lead to the full exploitation of the data, including enhanced and timely decision-making and successful environmental quality monitoring of the marine environment.

metaGOflow was developed with the ultimate objective to build a distributed workflow for analyses of marine metagenomic data generated by GOs such as EMO BON. The modular notion of metaGOflow allows us to perform the steps related to the taxonomy inventories and at a later point investigate the functional potential of a sample. Taxonomic inventories, essential for the case of GOs, are retrieved in a few hours. The functional annotation, as implemented, is highly time-consuming compared to any other step of the workflow. That is mostly because of the InterProScan implementation; the vast amount of sequences but also the standalone module with which the scan is performed lead to long single-threaded processes. However, once the clustermode will be as fault tolerant as the standalone, metaGOflow will adopt it. On top of that, optimizations on the implementation of the InterProScan step would decrease further the total time for the complete analysis. MEGAHIT provides an assembly of the reads that can then be used with the corresponding MGnify workflow for further analysis. Ultimately, using the parallel option of the cwltool combined with HPC environments and its modular notion, metaGOflow, enables the effective, on-time, and valid analysis of GO data.

metaGOflow packages all its output, the workflow’s metadata, and the user’s settings in RO-crates, which is a novel feature in metagenomics bioinformatics analysis pipelines, to the best of our knowledge and as mentioned in Table 1. This novelty in the workflow’s implementation allows the EMO BON community to access all data products, along with details on the employed methods, in a machine-readable way, either directly (see Zenodo example [74]) or through portals such as MGnify. Thus, it is now far easier for data and data products to be reused for meta-analyses but also to be exploited by data integration approaches [80, 81].

CWL (i.e., the language that the workflow is built on) has certain drawbacks. Among them, the requirement for explicit input–output declarations, the fact that the Javascript ExpressionTools may affect the portability of the workflow, and mainly being a data-driven “dataflow” mean that handy control workflow patterns (e.g., loops) cannot be used [82]. However, some other features of the language (i.e., its modularity and its consistency when combined with containerization technologies) allowed us to build on top of the well-established MGnify environment; thus, metaGOflow enables the robust, standardized, and fast-enough analysis of GO data. By all means, other workflow managers, such as Nextflow [28], may also support such community efforts. Toil [83] and similar technologies will be investigated for better exploitation of the provided computing resources, as well as cloud-based implementations of the workflow. The future integration of metaGOflow in e-infrastructures will be also considered.

The need for different approaches in the analysis of the shotgun metagenomics raw data has been well established [76]. metaGOflow’s data products, like the output of any bioinformatics analysis of shotgun metagenomics data [84], may be explored in various ways through a great range of downstream analysis. Questions about key taxa in a sample or in a group of samples, about essential metabolic pathways that characterize a sample or a group of samples compared with others and so on, can now be addressed using the findings of shotgun metagenomics analysis as input. Liu et al. [85] distinguish the possible downstream analysis in “overall,” exploring differences in alpha/beta-diversity and taxonomic composition in a feature table, and “details analysis,” identifying biomarkers via comparison (using correlation and/or network analysis, machine learning, etc.).

metaGOflow adds to a list of similar approaches such as nf-core/mag [86], metaWRAP [22], MG-RAST [32], JGI-IMG [34], and bioBakery 3 (MetaPhlan 3) [23]. metaGOflow highlights the potential that modern workflow managers and containerization technologies support for building workflows upon workflows. Regarding raw data deriving from GOs, metaGOflow facilitates data generation and, subsequently, interpretation of times-series biodiversity data, thus granting valuable insights to the scientific community and building a solid foundation for long-term sustainable and high-value data outputs. Long-term sustainability is ensured by the FAIRness of the outputs and the strategic support of the EMBRC-ERIC infrastructure. Moreover, even if it was initially developed to address the specific needs of a GO project such as EMO BON, metaGOflow is overall a user-friendly flexible workflow that can be broadly used for one-sample-at-a-time analysis of shotgun metagenomics data.

## Availability of Source Code and Requirements

- Project name: metaGOflow: A workflow for marine Genomic Observatories data analysis
- Project homepage: https://github.com/emo-bon/MetaGOflow
- Manual page: https://metagoflow.readthedocs.io
- WorkflowHub: https://workflowhub.eu/workflows/384
- RRID: SCR_023674
- biotools id: metagoflow
- Operating system(s): Unix
- Programming language: Common Workflow Language (CWL)
- Other requirements: Docker or Singularity engines. Node.js is required in cases where Docker is not available.
- License: Apache License 2.0. For third-party components separate licenses apply. Any restrictions to use by nonacademics: license needed.

## Supplementary Material

giad078_GIGA-D-23-00127_Original_Submission

giad078_GIGA-D-23-00127_Revision_1

giad078_GIGA-D-23-00127_Revision_2

giad078_GIGA-D-23-00127_Revision_3

giad078_GIGA-D-23-00127_Revision_4

giad078_Response_to_Reviewer_Comments_Original_Submission

giad078_Response_to_Reviewer_Comments_Revision_1

giad078_Response_to_Reviewer_Comments_Revision_3

giad078_Response_to_Reviewer_Comments_Revision_4

giad078_Reviewer_1_Report_Original_Submission Artem Barski -- 6/8/2023 Reviewed

giad078_Reviewer_1_Report_Revision_1 Artem Barski -- 7/17/2023 Reviewed

giad078_Reviewer_2_Report_Original_Submission Samuel Lampa, PhD -- 6/10/2023 Reviewed

giad078_Supplemental_File

## Data Availability

Snapshots of our code and other data further supporting this work are openly available in the *GigaScience* repository, GigaDB 102443 [87]. All the raw sequence files of this study are available at ENA [43]: EMO BON super study accession number PRJEB51688 [88] EMO BON marine sediment sample: run accession number ERS14961254 [89], study accession number PRJEB51652 [90] EMO BON water column sample: run accession number ERS14961281 [91], study accession number PRJEB51664 [92] Tara Oceans sample: run accession number ERR599171 [93], study accession number PRJEB402 [94]

## Additional Files

**Supplementary Table S1**. metaGOflow results for the 2 gut samples. ERR4765907: fish gut sample; SRR9654976: human gut sample.

## Abbreviations

bp: base pair; CDS: coding sequences; CWL: Common Workflow Language; EMBRC: European Marine Biological Resource Centre; EMO BON: European Marine Omics Biodiversity Observation; ENA: European Nucleotide Archive; Gb: gigabase; GO terms: Gene Ontology terms; GOs: Genomic Observatories; HPC: high-performance computing; LSU: large subunit; RO-Crate: Research Object Crate; SSU: small subunit.
