# Supplementary material for: metaGOflow: a workflow for the analysis of marine Genomic Observatories shotgun metagenomics data
Source: Gigascience. 2023 Oct 18;12:giad078. doi: 10.1093/gigascience/giad078 (PMC10583283; doi:10.1093/gigascience/giad078)
Supplement: giad078_GIGA-D-23-00127_Revision_1 [file giad078_giga-d-23-00127_revision_1.pdf]

GigaScience

metaGOflow: a workflow for the analysis of marine Genomic Observatories shotgun metagenomics data

--Manuscript Draft--

**Manuscript Number:**	GIGA-D-23-00127R1
**Full Title:**	metaGOflow: a workflow for the analysis of marine Genomic Observatories shotgun metagenomics data
**Article Type:**	Technical Note
**Funding Information:**	HORIZON EUROPE European Research Council (824087) — Not applicable<br>European Marine Biological Resource Centre — Not applicable
**Abstract:**	Background: Genomic Observatories (GOs) are sites of long-term scientific study that undertake regular assessments of the genomic biodiversity. The European Marine Omics Biodiversity Observation Network (EMO BON) is a network of GOs that conduct regular biological community samplings to generate environmental and metagenomic data of microbial communities from designated marine stations around Europe. The development of an effective workflow is essential for the analysis of the EMO BON metagenomic data in a timely and reproducible manner.<br>Findings: Based on the established MGnify resource we developed metaGOflow; metaGOflow supports the fast inference of taxonomic profiles from GO-derived data based on rRNA genes and their functional annotation using the raw reads. Thanks to the Research Object Crate (RO-Crate) packaging, relevant metadata about the sample under study, and the details of the bioinformatics analysis it has been subjected to, are inherited to the data product while its modular implementation allows running the workflow partially. The analysis of two EMO BON and one Tara Oceans samples was performed as a use case.<br>Conclusions: metaGOflow is an efficient and robust workflow that scales to the needs of projects producing big metagenomic data such as EMO BON. It highlights how containerization technologies along with modern workflow languages and metadata package approaches can support the needs of researchers when dealing with ever-increasing volumes of biological data. Despite being initially oriented to address the needs of EMO BON, metaGOflow is a flexible and easy-to-use workflow that can be broadly used for one-sample-at-a-time analysis of shotgun metagenomics data.
**Corresponding Author:**	Haris Zafeiropoulos<br>KU Leuven Rega Institute for Medical Research.: Katholieke Universiteit Leuven Rega Institute for Medical Research<br>Leuven, BELGIUM
**Corresponding Author Secondary Information:**	
**Corresponding Author's Institution:**	KU Leuven Rega Institute for Medical Research.: Katholieke Universiteit Leuven Rega Institute for Medical Research
**Corresponding Author's Secondary Institution:**	
**First Author:**	Haris Zafeiropoulos
**First Author Secondary Information:**	
**Order of Authors:**	Haris Zafeiropoulos

Powered by Editorial Manager® and ProduXion Manager® from Aries Systems Corporation

	Martin Beracochea
	Stelios Ninidakis
	Katrina Exter
	Antonis Potirakis
	Gianluca De Moro
	Lorna Richardson
	Erwan Corre
	João Machado
	Evangelos Pafilis
	Ioulia Santi
	Georgios Kotoulas
	Robert Daniel Finn
	Cymon Cox
	Christina Pavloudi
**Order of Authors Secondary Information:**	
**Response to Reviewers:**	Our point-by-point response has been uploaded as a separate file.
**Additional Information:**	
**Question**	**Response**
Are you submitting this manuscript to a special series or article collection?	No
**Experimental design and statistics**<br><br>Full details of the experimental design and statistical methods used should be given in the Methods section, as detailed in our Minimum Standards Reporting Checklist. Information essential to interpreting the data presented should be made available in the figure legends.<br><br>Have you included all the information requested in your manuscript?	Yes
**Resources**<br><br>A description of all resources used, including antibodies, cell lines, animals and software tools, with enough information to allow them to be uniquely identified, should be included in the Methods section. Authors are strongly encouraged to cite Research Resource	Yes

Identifiers (RRIDs) for antibodies, model organisms and tools, where possible.<br><br>Have you included the information requested as detailed in our Minimum Standards Reporting Checklist?	
**Availability of data and materials**<br><br>All datasets and code on which the conclusions of the paper rely must be either included in your submission or deposited in publicly available repositories (where available and ethically appropriate), referencing such data using a unique identifier in the references and in the "Availability of Data and Materials" section of your manuscript.<br><br>Have you have met the above requirement as detailed in our Minimum Standards Reporting Checklist?	Yes

Manuscript
Click here to access/download;Manuscript;marine_GOs_CWL_workflow_rev

OXFORD (GIGA)^n SCIENCE

GigaScience, 2022, 1–10

doi: xx.xxxx/xxxx
Manuscript in Preparation
Technical Note

TECHNICAL NOTE

metaGOflow: a workflow for the analysis of marine Genomic Observatories shotgun metagenomics data

Haris Zafeiropoulos[1,2] *, Martin Beracochea[3] *, Stelios Ninidakis[1], Katrina Exter[4], Antonis Potirakis[1], Gianluca De Moro[5], Lorna Richardson[3], Erwan Corre[6], João Machado[5], Evangelos Pafilis[1], Georgios Kotoulas[1], Ioulia Santi[7,1], Robert D. Finn[3], Cymon J. Cox[5] and Christina Pavloudi[1,8] †

[1] Institute of Marine Biology, Biotechnology and Aquaculture (IMBBC), Hellenic Centre for Marine Research (HCMR), Former U.S. Base of Gournes P.O. Box 2214, 71003, Heraklion, Crete, Greece and [2] KU Leuven, Department of Microbiology, Immunology and Transplantation, Rega Institute for Medical Research, Laboratory of Molecular Bacteriology, 3000 Leuven, Belgium and [3] European Molecular Biology Laboratory, European Bioinformatics Institute (EMBL-EBI), Wellcome Genome Campus, Hinxton, Cambridge CB10 1SD, UK and [4] Flanders Marine Institute (VLIZ), Oostende, Belgium and [5] Centro de Ciências do Mar (CCMAR), Universidade do Algarve, Campus de Gambelas, 8005-139, Faro, Portugal and [6] CNRS, FR 2424, ABiMS Platform, Station Biologique de Roscoff (SBR), Roscoff, France and [7] European Marine Biological Resource Centre (EMBRC-ERIC), Paris, France and [8] Department of Biological Sciences, The George Washington University, District of Columbia, USA

* Corresponding authors: haris.zafeiropoulos@kuleuven.be & mbc@ebi.ac.uk
† Current affiliation: PSL Research University: EPHE-UPVD-CNRS, UAR CNRS 3278 Centre de Recherche Insulaire et Observatoire de l'Environnement (CRIOBE), France & Laboratoire d'Excellence "CORAIL", Centre de Recherche Insulaire et Observatoire de l'Environnement (CRIOBE), French Polynesia

Abstract

Background: Genomic Observatories (GOs) are sites of long-term scientific study that undertake regular assessments of the genomic biodiversity . The European Marine Omics Biodiversity Observation Network (EMO BON) is a network of GOs that conduct regular biological community samplings to generate environmental and metagenomic data of microbial communities from designated marine stations around Europe. The development of an effective workflow is essential for the analysis of the EMO BON metagenomic data in a timely and reproducible manner.
Findings: Based on the established MGnify resource we developed metaGOflow; metaGOflow supports the fast inference of taxonomic profiles from GO-derived data based on rRNA genes and their functional annotation using the raw reads. Thanks to the Research Object Crate (RO-Crate) packaging, relevant metadata about the sample under study, and the details of the bioinformatics analysis it has been subjected to, are inherited to the data product while its modular implementation allows running the workflow partially. The analysis of two EMO BON and one Tara Oceans samples was performed as a use case.
Conclusions: metaGOflow is an efficient and robust workflow that scales to the needs of projects producing big metagenomic data such as EMO BON. It highlights how containerization technologies along with modern workflow languages and metadata package approaches can support the needs of researchers when dealing with ever-increasing volumes of biological data. Despite being initially oriented to address the needs of EMO BON, metaGOflow is a flexible and easy-to-use workflow that can be broadly used for one-sample-at-a-time analysis of shotgun metagenomics data.

Key words: shotgun metagenomics; MGnify; Common Workflow Language (CWL); containers; provenance; RO-Crate

Compiled on: June 30, 2023.
Draft manuscript prepared by the author.

Introduction

It is well established that microbial assemblages support multiple ecosystem services and that microbial community profiling using metagenomics methods can help elucidate the mechanisms that govern the structure of these communities and their interactions with the environment [1]. The community composition and structure of marine microbiome is directly correlated with environmental quality [2, 3]. Indeed, the quality of a marine microbial environment (e.g. a marine sediment) can impact the food chain [4] through the physical and chemical effects of secondary metabolites [5]. In addition, secondary metabolites produced by microorganisms may also become targets for bio-prospecting in medicine and industry [6]. Monitoring the changes in microbial community composition and function due to climate change-related stressors, such as ocean acidification or increases in temperature and UV absorption, can provide insights on ecosystem function, health, and resilience [7].

Pioneering research programmes such as the Ocean Sampling Day (OSD) [8], Malaspina circumnavigation expedition [9], and Tara Oceans [10], have been instrumental in collecting large series' of marine genomic samples from sites around the globe. The analysis of data resulting from these studies has greatly increased our understanding of the importance, the role, and the mechanisms governing microbial communities in some of the most common, sensitive or threatened marine environments [11, 12, 13]. EMO BON [14], a European Marine Biological Resource Centre (EMBRC-ERIC) initiative, is designed to continue and expand this effort by regular bimonthly microbial genomic biodiversity samplings at designated marine coastal stations around the European coastline. In the first two years of the EMO BON (2021-2022) it is expected that more than 540 shotgun metagenomic data sets from water column and sediment samples will be generated from 17 European sites.

The ultimate success of GOs depends on the development and adoption of standards for sampling, metadata collection, sequencing, and data analysis. The provision of metadata relating to the raw sequence data, data products, and their analysis methods, are of high importance for interpretation and interoperability, and need to be accessible in both human- and machine-readable formats. Legislative framework, such as the Nagoya Protocol for Access and Benefit Sharing (ABS) [15], and community written frameworks, such as those developed by the Genomic Standards Consortium (GSC) [16], as well as initiatives encouraging adherence to best practices, such as the Better Biomolecular Ocean Practices (BeBOP) project [17], have all been key to providing agreed-upon standard that aim to fulfil these needs. Standard operating procedures and standardised methods of analysis enable the comparison of results among sites, through time, and among projects, without which, much of the value of the data for environmental assessment is lost.

Effective analysis of shotgun metagenomic data is time-consuming, especially regarding computational steps such as sequence assembly and annotation [18]. Moreover, microbial community profiling and functional analyses are most useful when samples are maximally comparable in space and time, and have been thereby treated using the same analytical procedures. To address the challenges that arise when analyzing metagenomic data, numerous workflows and pipelines have been developed. Notable pipelines include metaWRAP [19], bioBakery [20], and nf-core [21], which provides a collection of pipelines such as nf-core/ampliseq [22] and nf-core/taxprofiler [21]. Recently, containerization approaches (e.g., Docker [23], Singularity [24] etc.), along with workflow managers (e.g., Nextflow [25], Snakemake [26] etc.), have been widely used to a) address the complexity of the analysis, b) facilitate execution and reproducibility and c) distribute and share software to a broader audience [27]. nf-core and ATLAS [28] shotgun metagenomic analysis pipelines are examples of the implementation of such approaches.

Additionally, there are (data analysis) resources like MG-RAST [29], MGnify [30], and IMG/M [31] that come with their own distinct advantages and disadvantages.

The computing requirements for the analysis of the EMO BON data may exceed the computing capacity that a single research institute and/or a regional High Performance Computing (HPC) (i.e., Tier 2) systems can support using the available workflows. Indicatively, for a single dataset, software tools related to the retrieval of taxonomic profiles require up to 160 CPU hours and up to 100 GB of RAM [32]. Computing requirements for the functional annotation of shotgun reads are even higher. Nevertheless, timely provision of data and data products from GOs is of paramount importance to facilitate long-term ecological studies, to accelerate policy-making, and to directly assess the impact of anthropogenic effects on the marine environment.

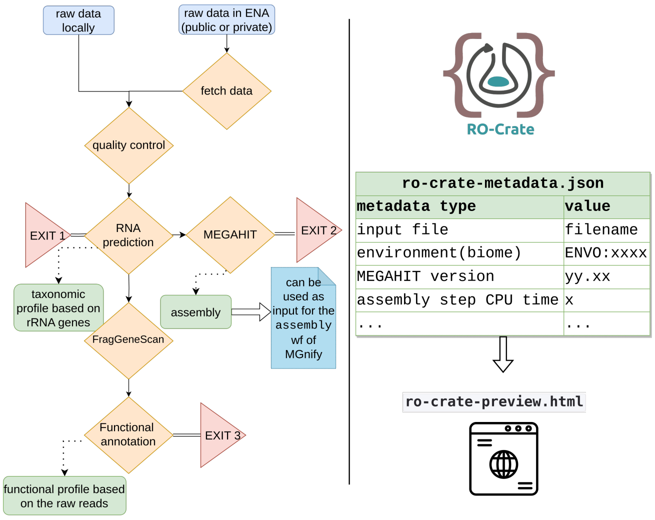

Figure 1. Schematic overview of metaGOflow, showing the main steps of the analysis along with their corresponding data products; the partial execution of the workflow is also shown by the potential exit points (left). Independent of the steps to be performed, once completed, an RO-Crate is built (right).

To address the challenges of analysing GO data in a timely and standardised framework we developed metaGOflow: a MGnify-based [30] computational workflow that implements the critical steps of a shotgun metagenomic bioinformatics analysis, and provides rich provenance metadata describing the data, data products, and workflow execution (Figure 1). The novel aspects of this workflow are mainly a) partial workflow execution; e.g. the user has the flexibility to choose whether to run the functional annotation sub-workflow or not, or even run it at a later point using the data products of the previous steps, b) the incorporation of an alternative assembler with a significantly lower computational cost as compared to the MGnfiy default one and c) the ultimate generation and verification of a Research Object (RO) crate ensuring the workflow's FAIRness. On top of that, several updates of the databases and tools invoked by MGnify have been performed.

metaGOflow consists of two basic concepts:

- an *analytical workflow* which provides taxonomic inventories and community gene function profiles of the samples as data products packaged in RO Crates [33],
- a *data provenance workflow* that generates extensive metadata and thereby provides compliance of the data, data products, and analytical procedures with FAIR data practices and the principles of Open Science, also packaged in the RO Crates [34, 17].

Implementation

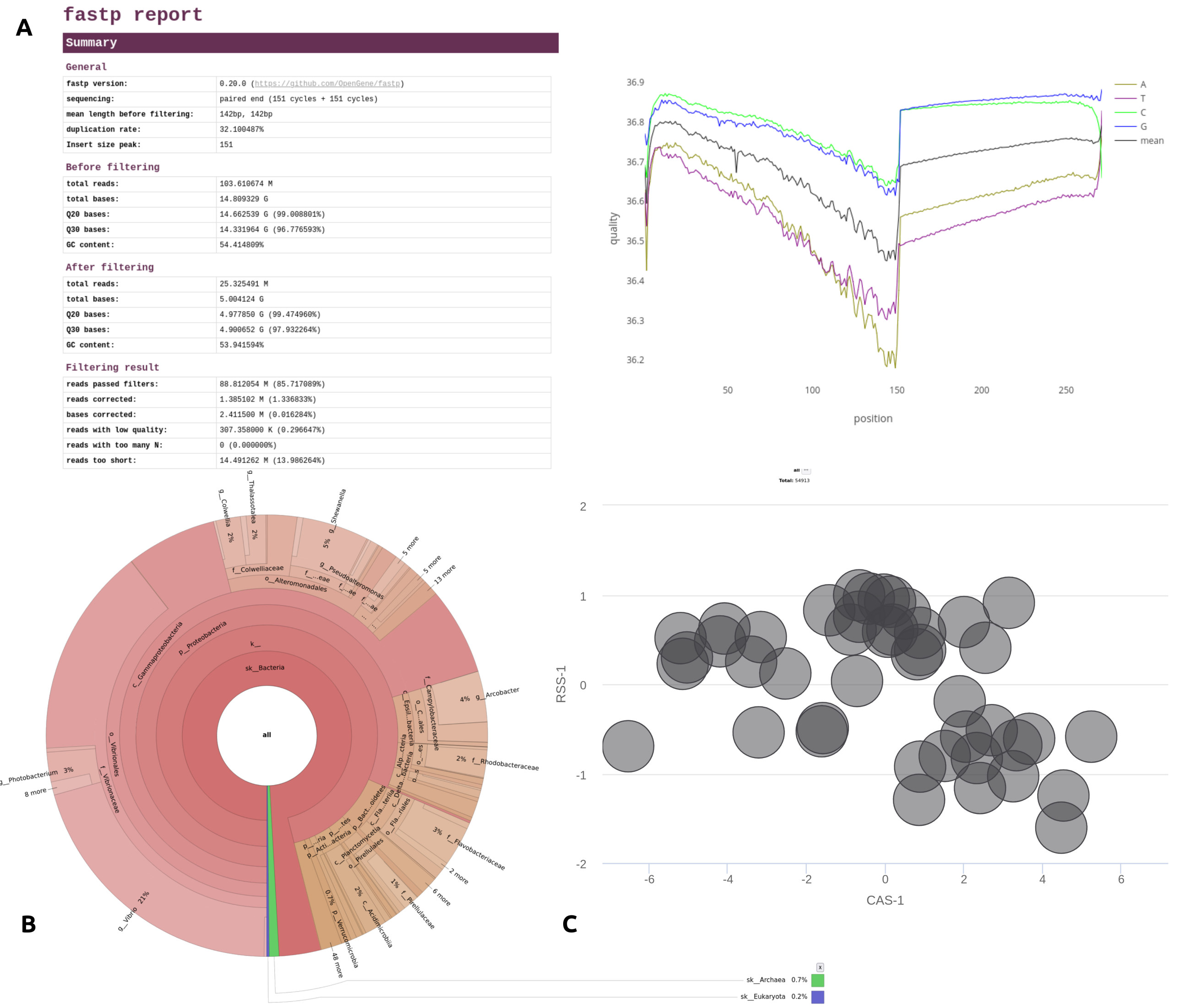

Figure 2. Visualisation of metaGOflow's main output. A. Raw data are first filtered and only high quality sequences are analysed further in the next steps. An .html file with the report of the merged reads is produced. Here, an excerpt of this report is shown: reads' statistics before and after filtering (left), ATGC chart with the quality of each base cycle-after-cycle for the merged reads (right) B. The taxonomy inventory step returns Molecular Operational Taxonomic Units (mOTUs) and the taxonomic composition based on the Large Sub Unit (LSU) and the Small Sub Unit (SSU) genes. Here, the taxonomic composition is represented by a Krona interactive visualization. C. The functional annotation step returns text files with the Gene Ontology (GO), KEGG, InterProScan, and Pfam terms retrieved. The retrieved GO terms are presented using Navigo [35], the Co-occurrence Association Score (CAS-1) and the Relevance Semantic Similarity (RSS-1). The Gene prediction step returns a `.ffn` and a `.faa` file while the assembly step a `.fasta` file including the contigs retrieved. The main output of the provenance feature is the `ro-crate-metedata.json` file.

Overview

The pillars around which metaGOflow has been built, namely containerisation technologies such as Docker [23] and Singularity [24], and the Common Workflow Language (CWL) [36], ensure the workflow's ability to perform in different HPC and cloud computing platforms, following the MGnify example.

metaGOflow inherits the architecture of MGnify pipeline-v5 and exploits several of the already containerized tools and the sub-workflows implemented in the MGnify pipeline. Several enhancements and upgrades allow metaGOflow to make use of the latest versions of the tools and databases invoked. metaGOflow makes extensive use of CWL *subworkflows* and *conditional* step execution to address the specific needs of the EMO BON project from a computing resources point of view.

For example, the user can run the workflow to only generate the taxonomic inventory of a sample. Then, at a later time and by using the output of the first analysis, the user can also generate the assembly of this sample's reads and/or their functional annotation. This flexibility in the workflow is essential as there are a considerable number of samples to be analysed (preferably in as short a period of time as possible), and the computing requirements, especially for the functional annotation step, can be substantial (see Table 1).

In its current version (v1.0.1), metaGOflow has 5 distinct steps. As in MGnify, metaGOflow analyses a single sample at a time (see Figure 1). The user may either provide locally stored raw data (`.fastq` files) or start the workflow by giving a European Nucleotide Archive (ENA)[37] run accession number. In the later case, metaGOflow invokes the `fetch_tool` [38] to retrieve the raw sequence files from ENA; if the data to be retrieved are held privately, the username and password of the associated ENA account are also requested. The user sets the steps of the workflow to be performed and provides values for certain tool parameters through a text-based configuration file (`config.yml`).

To enhance the FAIRness of the data products and of the bioinformatic analysis, metaGOflow data products are packages as RO-Crates: this allows the set of files to be semantically described, to be accompanied by the metadata that describe the precise steps of the

workflow execution, the tools and the parameters used, and to flag the specific input and output files. This description is provided in a JSON-LD file following a particular (user-generated) profile. Along with the data products, the RO-crate contains information describing the version of the workflow *per se*, including the software and database versions that it uses.

The development and testing of metaGOflow was performed in the IMBBC HCMR "Zorbas" HPC [27] and at the HPC facility of CCMAR. Further testing was performed on the Luxembourg national supercomputer MeluXina. The use case experiments (see Section 4) were performed in a "fat" node of the "Zorbas" HPC (2x Inter(R) Xeon(R) Gold 6230 CPU @ 2.10GHz 40 cores and 500 GB). A thorough description of how to install and use metaGOflow, as well as common errors that might occur during the analysis of a sample can be found at wiki page of the metaGOflow GitHub repository. A Continuous Integration/Continuous Deployment (CI/CD) workflow using GitHub Actions ensures the validity of the workflow's `cwl` main script and, therefore, of all its components. The databases to be installed before using metaGOflow, require 160GB of storage and as a rule of thumb, the user should allocate 1TB of storage to perform a metaGOflow analysis.

A comparison of the main features of metaGOflow with other commonly used pipelines for shotgun metagenomic analysis is given in Table 2.

Step 1: Sequence preprocessing

Sequences are filtered and merged using `fastp` (version 0.20.0) [39]. Short, low quality, and non-merging sequences are removed and a series of statistical tests describing the quality of the sequencing are performed.

Step 2: Taxonomy inventory

metaGOflow makes use of the `esl-sfetch` miniapp of the EASEL library (S.R. Eddy, unpublished) to index the filtered sequences and support fast sequence retrieval. Then `cmsearch`, an Infernal program [40], is performed using the ribosomal and the non-coding RNA (ncRNA) Rfam covariance models (CM) (version v13.0) against the filtered sequences. Eventually, this is followed by taxonomic classification using `MAPseq` (v1.2.3) [41] and the SILVA database (version 132) for the taxonomic classification of the SSU and the LSU sequences, while `mOTUs2` [42] quantifies both known and unknown taxa on the filtered sequences.

Step 3: Assembly

Shotgun metagenomic read assembly requires significant computing resources as discussed in Mitchell et. *al* [30] and in Vollmers et. *al* [43]. The extent of the computational "burden" depends heavily on the chosen algorithm. To be able to handle the vast amount of data produced by EMO BON in a timely manner, and since we aim more at unravelling biodiversity at the community, rather than at the individual (i.e. species), level, metaGOflow makes use of the MEGAHIT algorithm [44]. Longer contigs would be returned if e.g. metaSPAdes [45] was employed, but given metaGOflow's high pace data generation and analysis needs, the MEGAHIT algorithm seems a better match.

Step 4: Gene prediction on the reads

metaGOflow performs gene prediction using FragGeneScan (v1.20) [46] like MGnify. This step is a prerequisite for the functional annotation of the reads (Step 5). To partially run this step, the user needs to provide the merged filtered `.fasta` file, provided by the sequence preprocessing step.

Step 5: Functional annotation of the reads

metaGOflow focuses on the potential metabolic processes of the whole community rather than the processes of each individual species. Therefore, it performs functional annotation at the reads level. Using InterProScan (v.57-90) [47] metaGOflow annotates the reads with InterPro5 [48], PFam [49], TIGRFAM [50], ProSite patterns and profiles [51] and Gene Ontology (GO) [52] terms. A GO Slim oriented visualization (available at geneontology.org) is returned. EggNOG5 [53] annotation is also performed using to the eggnog-mapper (v2.1.8) [54]. Last, metaGOflow invokes the HMMER [55] tool along with the KOfam library [56] to get KEGG orthology annotations [57]. This step requires a significant amount of computing time.

Building RO-Crates

An RO-Crate is created automatically by the workflow to store the data products of the aforementioned steps, along with the MetaGOflow run associated metadata (including the user set parameters, the version and the source of the workflow used). To this end, the `rocrate` Python library [58, 59] is used. As mentioned, an RO-Crate object is accompanied by a JSON-LD file (called `ro-crate-metadata.json`), part of which is shown in Figure 3, which includes the descriptions of both input and output files.

Use case

To demonstrate metaGOflow and its key features, the analysis of a sediment and a water column sample from EMO BON was performed. As mentioned in the EMO BON handbook [60] and the EMO BON paper [14], DNA extraction, cleaning, library preparation and sequencing is performed at a centralised facility to minimize biases and maximize consistency in sequence quality. DNA extraction is performed using commercially available kits, to minimise deviations among samples. The samples were randomly chosen from two different stations but are considered to be representative EMO BON data. Moreover, an already publicly available marine metagenome sample from the Tara Oceans expedition [61], with size (in Gb) similar to those of the EMO BON data, was also analysed. All steps of metaGOflow were performed for each of these samples and the computational time (in hours), and the maximum memory (RAM, in GB) are reported in Table 1. Additionally, to demonstrate the applicability of metaGOflow for all types of shotgun metagenomic

Table 1. Computing requirements for the analysis of a sediment and a water column EMO BON sample as well as a Tara Oceans water sample, using metaGOflow in a "fat" node of the Zorba HPC.

workflow step(s)	computational time (hours)			memory (max RAM in Gb)		
	EB sediment	EB water	TO water	EB sediment	EB water	TO water
prepr. & taxon. invent. (Steps 1&2)	14.5	12.6	26.4	4.55	4.65	4.15
assembly (Step 3)	1.6	1.22	0.4	8.8	4.38	2.7
gene calling & funct. annot. (Steps 4&5)	98.7	92.4	84.2	205.1	188.6	155.4

EB: EMO BON, TO: Tara Oceans.

Table 2. Comparison of the main features and implementation of pipelines similar to metaGOflow.

Category	Feature	MetaWRAP	ATLAS	nf-core/taxprofiler	nf-core/funcscan	metaGOflow
	Quality control	fastqc	-	fastp, falco	-	fastp
Pre-processing	Filtering	Trim Galore	BBTools	porechop, fastp, bbduk, prinseq++, Filtlong	-	fastp
	Host-read removal	bmtagger	-	Bowtie2 for short reads and minimap2 for long reads	-	-
	Taxonomy assignment of rRNA genes	-	-	-	-	mOTUs, MAPseq
Taxonomy	Taxonomic assignment of reads and/or contigs	kraken, kraken2	-	Kraken2, DIAMOND, mOTUs, MetaPhlAn3, MALT	-	-
	Taxonomic assignment of bins	TAXATOR-TK	GTDB-tk	-	-	-
	Short read assembly	metaspades and/or MEGAHIT	MEGAHIT	-	-	MEGAHIT
Assembly	Hybrid assembly	-	Yes	-	-	-
	Group-wise co-assembly	Yes	Yes	-	-	-
	Genome binning	metaBAT2, MaxBin2, CONCOCT	metabat2, maxbin2	-	-	-
BINs-MAGs	Bin refinement	Binning-refiner	-	DAS Tool	-	-
	Gene prediction	-	-	prodigal	-	FragGeneScan
Annotation	Functional annotation	prokka (using the bins)	eggNOG	-	hAMRonization, AMPcombi, comBGC.py	InterProScan, eggNOG, hmmsearch
	Ontologies	-	eggNOG	-	-	KEGG, GO, pfam, eggNOG, InterPro
	keeping track of sample's metadata	-	-	-	-	Yes
FAIR-ness	output as RO-Crate	-	-	-	-	Yes
	workflow provided through containers	-	-	-	Yes	Yes
Architecture	workflow manager	-	snakemake	nextflow	nextflow	cwl

Table 3. metaGOflow results for the two EMO BON samples (marine sediment and a water column) and the Tara Oceans (seawater) sample.

product	EMO BON sediment	EMO BON water	Tara Oceans water
total reads (M)	51.8	44.0	36.5
filtered reads (M)	33.2	28.2	19.9
SSU	438	361	345
LSU	719	469	444
contigs	348,405	338,467	102,343
Reads with predicted CDS (M)	32.4	27.4	18.8
Pred. CDS* with IPS match (M)	9.9	9.4	5.2
Pred. CDS with GO match (M)	5.4	5.6	3.2
Pred. CDS with Pfam match (M)	9.3	8.9	4.9
Pred. CDS with KO match (M)	1.0	1.15	0.5

M: millions, *CDS: Coding Sequences

data, it was implemented for the analysis of a fish gut and a human gut metagenomic sample. All five samples were sequenced in different platforms: NovaSeq (EMO BON), HiSeq 2000 (Tara Oceans), BGISEQ-500 (fish gut), NextSeq 550 (human gut). The metaGOflow results for the gut samples are included in the zenodo repository and the respective statistics are given in Supplementary Table 1.

Raw sequences were preprocessed using 130 bp as the minimum length of the reads and at least 30 bp of overlap for the merging step for the 2 EMO BON samples. In case of the Tara Oceans sample, a minimum length of 108 bp was used as the sequences were shorter. The pre-processing and the taxonomic inventory step lasted about from 10 to 24 hours. By allocating a computing node similar to the one used for the use case, taxonomic inventories from at least 300 metagenomes could be produced per year, based on the results from the EMO BON samples.

For the assembly step, a minimum contig length of 200 bp was used for all the samples. The assembly of the reads using the MEGAHIT algorithm was performed in less than 2 hours, while the maximum memory required was less than 10Gb which is at least one order of magnitude less than what other software, e.g. metaSPAdes, would require. The large number of contigs returned suggests one could aim for a higher minimum contig length. For example, using a minimum contig length of 500 bp for the Tara Oceans sample, the number of contigs was decreased from 102, 343 (Table 1) to 34, 426 and the required time was about 30 minutes.

The gene calling and the functional annotation steps were those requiring the most computing resources, as expected. For each of the three samples, it took about 4 days to complete these steps, with the InterProScan part being the most computationally expensive with respect to both time and memory. In order for metaGOflow to exploit the available computing resources in an optimal way, the user is strongly advised to follow the "Improving performance" instructions of InterProScan and set the relative arguments accordingly.

A summary of the metaGOflow outputs and their respective size for this use case is shown in Table 3. A visual representation of the detailed results (quality control report, taxonomic inventories, functional annotations) of the workflow can be found through this GitHub page. An example of the complete data product of metaGOflow, packed in a RO-Crate, can be found through this Zenodo repo. For the EMO BON samples, the default configuration files `config.yml` were used; for the Tara Oceans sample, the `config.yml` is included in the respective RO-crate object, which is available in the Zenodo repository.

Based on the scientific questions to be addressed, several types of downstream statistical analysis using the metaGOflow data products might be performed. Most of these statistical approaches are not specific for the analysis of metagenomic datasets *per se* [62]. Contrary, they are well established in several research communities: microbial ecologists, microbiologists, medical scientists. However, the nature of the metagenomic data lead to several challenges, such as the "compositional effect" that need to be dealt to the best possible extent [63, 64].


```
{
    "@id": "results/functional-annotation/stats/interproscan.stats",
    "@type": "File",
    "encodingFormat": "text/plain",
    "name": "InterProScan summary statistics"
},
{
    "@id": "results/functional-annotation/stats/go.stats",
    "@type": "File",
    "encodingFormat": "text/plain",
    "name": "Geno Ontology summary statistics"
},
{
    "@id": "results/functional-annotation/stats/ko.stats",
    "@type": "File",
    "encodingFormat": "text/plain",
    "name": "Kegg Ontology summary statistics"
},
{
    "@id": "results/functional-annotation/stats/pfam.stats",
    "@type": "File",
    "encodingFormat": "text/plain",
    "name": "Pfam summary statistcs"
},
{
    "@id": "results/functional-annotation/stats/orf.stats",
    "@type": "File",
    "encodingFormat": "text/plain",
    "name": "ORF summary statistics"
},
{
    "@id": "https://www.apache.org/licenses/LICENSE-2.0",
    "@type": "CreativeWork",
    "identifier": "https://spdx.org/licenses/Apache-2.0.html",
    "name": "Apache License 2.0"
},
```


Figure 3. Part of the `ro-crate-metadata.json` file describing the metaGOflow output files.

Discussion & conclusions

Metagenomic applications include different procedures and require expertise in different topics, from field sampling, to lab analyses, to sequencing [65]. This inevitably leads to delays in raw data production, let alone usable scientific results. On top of that, metagenomic raw data are not directly usable as they require time-consuming and computationally-demanding processing as well as specialized bioinformatics expertise [65, 62]. For EMO BON and other GOs to produce applicable and fit-for-purpose data, it is of huge importance that quality-controlled and standardised data, as well as informative data products, are made rapidly available. The disentanglement of the analyses from technical expertise and extensive computing infrastructures will allow the direct generation of meaningful data products, even by non-experts. There is a paramount added value to the provision of preliminary results and data products (i.e. taxonomic inventories) from metagenomic GO samples as it can lead to the full exploitation of the data, including enhanced and timely decision-making and successful environmental quality monitoring of the marine environment.

metaGOflow was developed with the ultimate objective to build a distributed workflow for analyses of marine metagenomic data generated by GOs such as EMO BON. The modular notion of metaGOflow allows us to perform the steps related to the taxonomy inventories and at a later point investigate the functional potential of a sample. Taxonomic inventories, essential for the case of GOs, are retrieved in a few hours. The functional annotation, as implemented, is highly time consuming compared to any other step of the workflow. That is mostly because of the InterProScan implementation; the vast amount of sequences but also the `standalone` module with which the scan is performed, lead to long single threaded processes. However, once the `clustermode` will be as fault tolerant as the `standalone`, metaGOflow will adopt it. On top of that, optimisations on the implementation of the InterProScan step would decrease further the total time for the complete analysis. MEGAHIT provides an assembly of the reads that it can then be used with the corresponding MGnify workflow for further analysis. Ultimately, using the parallel option of the `cwltool` combined with HPC environments and its modular notion, metaGOflow enables the effective, on time and valid, analysis of GOs data.

metaGOflow packages all its output, the workflow's metadata as well as the user's settings, in RO-crates, which is a novel feature in metagenomics bioinformatics analysis pipelines, to the best of our knowledge and as mentioned in Table 2. This novelty in the workflow's implementation allows the EMO BON community to access all data products, along with details on the employed methods, in a machine-readable way, either directly (see Zenodo example) or through portals such as MGnify. Thus, it is now far easier for data and data products to be re-used for meta-analyses, but also to be exploited by data integration approaches [66, 67].

CWL, i.e. the language that the workflow is built on, has certain drawbacks. Among them, the requirement for explicit input-output declarations, the fact that the Javascript `ExpressionTools` may affect the portability of the workflow, and mainly being a data-driven "dataflow", means that handy control workflow patterns (e.g., loops) cannot be used [68]. However, some other features of the language, i.e. its modularity and its consistency when combined with containerization technologies, allowed us to build on top of the well-established MGnify environment; thus, metaGOflow enables the robust, standardized and fast-enough analysis of GO data. By all means, other workflow managers, such as Nextflow [25], may also support such community efforts. Toil [69] and similar technologies will be investigated for better exploitation of the provided computing resources, as well as cloud-based implementations of the workflow. The future integration of metaGOflow in e-infrastructures will be also considered.

metaGOflow adds to a list of similar approaches such as nf-core/mag [70], metaWRAP [19], MG-RAST [29], JGI-IMG [31], bioBakery 3 (MetaPhlan 3) [20]. metaGOflow highlights the potential that modern workflow managers and containerization technologies support for building workflows upon workflows. Regarding raw data deriving from GOs, metaGOflow facilitates data generation, and, subsequently, interpretation of times-series biodiversity data, thus granting valuable insights to the scientific community and building a solid foundation for long-term sustainable and high-value data outputs. Long-term sustainability is assured by the FAIRness of the outputs and the strategic support of the EMBRC-ERIC infrastructure. Moreover, even if it was initially developed to address the specific needs of a GO project such as EMO BON, metaGOflow is overall a user-friendly flexible workflow that can be broadly used for one-sample-at-a-time analysis of shotgun metagenomics data.

Availability of source code and requirements

- Project name: metaGOflow: A workflow for marine Genomic Observatories data analysis
- Project home page: https://github.com/emo-bon/MetaGOflow
- WorkflowHub: https://workflowhub.eu/workflows/384
- RRID: SCR_023674
- biotools id: metagoflow
- Operating system(s): Unix
- Programming language: Common Workflow Language (CWL)
- Other requirements: Docker or Singularity engines. Node.js is required in cases where Docker is not available.
- License: Apache License 2.0. For third-party components separate licenses apply. Any restrictions to use by non-academics: licence needed.

Availability of supporting data and materials

All the raw sequence files of this study are available at ENA [37]:

- EMO BON super study accession number PRJEB51688 (available at http://www.ebi.ac.uk/ena/data/view/PRJEB51688)
- EMO BON marine sediment sample: run accession number ERS14961254 (available at http://www.ebi.ac.uk/ena/data/view/ERS14961254), study accession number PRJEB51652 (available at http://www.ebi.ac.uk/ena/data/view/PRJEB51652)
- EMO BON water column sample: run accession number ERS14961281 (available at http://www.ebi.ac.uk/ena/data/view/ERS14961281), study accession number PRJEB51664 (available at http://www.ebi.ac.uk/ena/data/view/PRJEB51664)
- Tara Oceans sample: run accession number ERR599171 (available at https://www.ebi.ac.uk/ena/browser/view/ERR599171, study accession number PRJEB402 (available at https://www.ebi.ac.uk/ena/browser/view/PRJEB402)

Declarations

List of abbreviations

- CDS: Coding Sequences
- CWL: Common Workflow Language
- EMBRC: European Marine Biological Resource Centre
- EMO BON: European Marine Omics Biodiversity Observation Network
- ENA: European Nucleotide Archive
- GOs: Genomic Observatories
- HPC: High Performance Computing
- LSU: Large Sub Unit
- OSD: Ocean Sampling Day
- RO-Crate: Research Object Crate
- SSU: Small Sub Unit

Ethical Approval

Not applicable.

Consent for publication

Not applicable.

Competing Interests

M.B., L.R. and R.D.F. are members of the MGnify group that is part of the ELIXIR infrastructure. The authors declare that they have no other competing interests.

Funding

This project has received funding from the European Union's Horizon 2020 research and innovation programme under grant agreement No 824087, under the 1st EOSC-Life Digital Life Sciences Open Call (Project ID 14325) and by the European Marine Biological Resource Centre - European Research Infrastructure Consortium (EMBRC-ERIC), which is part of the European Strategy Forum on Research Infrastructures (ESFRI).

Author's Contributions

Conceptualization: C.J.C., R.D.F., C.P.; Project Administration: C.P., A.P., H.Z.; Investigation: H.Z., M.B., S.N., G.D.M., J.M.; Formal Analysis: H.Z.; Software: H.Z., M.B., S.N., J.M., C.J.C.; Methodology: H.Z., S.N., K.E., E.C.; Validation: H.Z., C.P., I.S.; Data Curation: I.S., K.E., C.P., H.Z.; Resources: I.S., R.D.F., L.R., C.J.C., E.P.; Funding Acquisition: C.P., G.K., C.J.C., H.Z., R.D.F.; Writing - Original Draft Preparation: H.Z., C.P.; Writing - Review & Editing: all; Visualization: H.Z.

Acknowledgements

This research was supported in part through computational resources provided by IMBBC (Institute of Marine Biology, Biotechnology and Aquaculture) of the HCMR (Hellenic Centre for Marine Research). Funding for establishing the IMBBC HPC has been received by the MARBIGEN (EU Regpot) project, LifeWatchGreece RI and the CMBR (Centre for the study and sustainable exploitation of Marine Biological Resources) RI. This study received Portuguese national funds from FCT - Foundation for Science and Technology through project UIDB/04326/2020, UIDP/04326/2020 and LA/P/0101/2020, and from the operational programmes CRESC Algarve 2020 and COMPETE 2020 through projects EMBRC.PT ALG-01-0145-FEDER-022121 and BIODATA.PT ALG-01-0145-FEDER-022231 to C.J.C and G.D.M. This work received Computational Time to HPC infrastructures and scientific and technical support from the high-level support team at NCC-Greece. The financial support from the EuroHPC-JU Project 101101903—EuroCC 2 project of the European Commission is acknowledged. Parts of the runs were performed on the MeluXina machine within the project with ID: EHPC-DEV-2022D10-062. The acquisition and operation of the EuroHPC supercomputer is funded jointly by the EuroHPC Joint Undertaking, through the European Union's Connecting Europe Facility and the Horizon 2020 research and innovation programme, as well as the Grand Duché du Luxembourg.

References

1. Louca S, Parfrey LW, Doebeli M. Decoupling function and taxonomy in the global ocean microbiome. Science 2016;353(6305):1272–1277.
2. Doney SC, Ruckelshaus M, Emmett Duffy J, Barry JP, Chan F, English CA, et al. Climate change impacts on marine ecosystems. Annual review of marine science 2012;4:11–37.
3. Chen J, McIlroy SE, Archana A, Baker DM, Panagiotou G. A pollution gradient contributes to the taxonomic, functional, and resistome diversity of microbial communities in marine sediments. Microbiome 2019;7(1):1–12.
4. Caruso G, La Ferla R, Azzaro M, Zoppini A, Marino G, Petochi T, et al. Microbial assemblages for environmental quality assessment: knowledge, gaps and usefulness in the European Marine Strategy Framework Directive. Critical reviews in microbiology 2016;42(6):883–904.
5. Caruso G, Azzaro M, Caroppo C, Decembrini F, Monticelli LS, Leonardi M, et al. Microbial community and its potential as descriptor of environmental status. ICES Journal of Marine Science 2016;73(9):2174–2177.
6. Liu X, Ashforth E, Ren B, Song F, Dai H, Liu M, et al. Bioprospecting microbial natural product libraries from the marine environment for drug discovery. The Journal of Antibiotics 2010;63(8):415–422.
7. Glasl B, Webster NS, Bourne DG. Microbial indicators as a diagnostic tool for assessing water quality and climate stress in coral reef ecosystems. Marine Biology 2017;164(4):1–18.
8. Kopf A, Bicak M, Kottmann R, Schnetzer J, Kostadinov I, Lehmann K, et al. The ocean sampling day consortium. Gigascience 2015;4(1):1–5.
9. Duarte CM. Seafaring in the 21st century: the Malaspina 2010 circumnavigation expedition. Limnology and Oceanography Bulletin 2015;.
10. Sunagawa S, Acinas SG, Bork P, Bowler C, Eveillard D, Gorsky G, et al. Tara Oceans: towards global ocean ecosystems biology. Nature Reviews Microbiology 2020;18(8):428–445.
11. Zayed AA, Wainaina JM, Dominguez-Huerta G, Pelletier E, Guo J, Mohssen M, et al. Cryptic and abundant marine viruses at the evolutionary origins of Earth's RNA virome. Science 2022;376(6589):156–162.
12. Sunagawa S, Coelho LP, Chaffron S, Kultima JR, Labadie K, Salazar G, et al. Structure and function of the global ocean microbiome. Science 2015;348(6237):1261359.
13. Yelton AP, Acinas SG, Sunagawa S, Bork P, Pedrós-Alió C, Chisholm SW. Global genetic capacity for mixotrophy in marine picocyanobacteria. The ISME journal 2016;10(12):2946–2957.
14. Santi I, Beluche O, Beraud M, Buttigieg P, Casotti R, Cox C, et al. European marine omics biodiversity observation network: a strategic outline for the implementation of omics approaches in ocean observation. Frontiers in Marine Science 2023;10:1118120.
15. Buck M, Hamilton C. The Nagoya Protocol on access to genetic resources and the fair and equitable sharing of benefits arising from their utilization to the Convention on Biological Diversity. Review of European Community & International Environmental Law 2011;20(1):47–61.
16. Kottmann R, Gray T, Murphy S, Kagan L, Kravitz S, Lombardot T, et al. A standard MIGS/MIMS compliant XML Schema: toward the development of the Genomic Contextual Data Markup Language (GCDML). Omics a journal of integrative biology 2008;12(2):115–121.
17. Samuel RM, Meyer R, Buttigieg PL, Davies N, Jeffery NW, Meyer C, et al. Toward a Global Public Repository of Community Protocols to Encourage Best Practices in Biomolecular Ocean Observing and Research. Frontiers in Marine Science 2021;p. 1488.
18. Tamames J, Cobo-Simón M, Puente-Sánchez F. Assessing the performance of different approaches for functional and taxonomic annotation of metagenomes. BMC genomics 2019;20(1):1–16.
19. Uritskiy GV, DiRuggiero J, Taylor J. MetaWRAP—a flexible pipeline for genome-resolved metagenomic data analysis. Microbiome 2018;6(1):1–13.
20. Beghini F, McIver LJ, Blanco-Míguez A, Dubois L, Asnicar F, Maharjan S, et al. Integrating taxonomic, functional, and strain-level profiling of diverse microbial communities with bioBakery 3. Elife 2021;10:e65088.
21. Ewels PA, Peltzer A, Fillinger S, Patel H, Alneberg J, Wilm A, et al. The nf-core framework for community-curated bioinformatics pipelines. Nature biotechnology 2020;38(3):276–278.
22. Straub D, Blackwell N, Langarica-Fuentes A, Peltzer A, Nahnsen S, Kleindienst S. Interpretations of environmental microbial community studies are biased by the selected 16S rRNA (gene) amplicon sequencing pipeline. Frontiers in Microbiology 2020;11:550420.
23. Merkel D. Docker: lightweight linux containers for consistent development and deployment. Linux journal 2014;2014(239):2.

24. Kurtzer GM, Sochat V, Bauer MW. Singularity: Scientific containers for mobility of compute. PloS one 2017;12(5):e0177459.
25. Di Tommaso P, Chatzou M, Floden EW, Barja PP, Palumbo E, Notredame C. Nextflow enables reproducible computational workflows. Nature biotechnology 2017;35(4):316–319.
26. Mölder F, Jablonski K, Letcher B, Hall M, Tomkins-Tinch C, Sochat V, et al. Sustainable data analysis with Snakemake [version 1; peer review: 1 approved, 1 approved with reservations]. F1000Research 2021;10(33).
27. Zafeiropoulos H, Gioti A, Ninidakis S, Potirakis A, Paragkamian S, Angelova N, et al. 0s and 1s in marine molecular research: a regional HPC perspective. GigaScience 2021;10(8):giab053.
28. ATLAS C, Yamamoto S, Shapiro M, Virzi J, Werner M, Venturi M, et al. The simulation principle and performance of the ATLAS fast calorimeter simulation FastCaloSim. ATL-COM-PHYS-2010-838; 2010.
29. Keegan KP, Glass EM, Meyer F. MG-RAST, a metagenomics service for analysis of microbial community structure and function. In: Microbial environmental genomics (MEG) Springer; 2016.p. 207–233.
30. Mitchell AL, Almeida A, Beracochea M, Boland M, Burgin J, Cochrane G, et al. MGnify: the microbiome analysis resource in 2020. Nucleic acids research 2020;48(D1):D570–D578.
31. Chen IMA, Chu K, Palaniappan K, Pillay M, Ratner A, Huang J, et al. IMG/M v. 5.0: an integrated data management and comparative analysis system for microbial genomes and microbiomes. Nucleic acids research 2019;47(D1):D666–D677.
32. Meyer F, Fritz A, Deng ZL, Koslicki D, Lesker TR, Gurevich A, et al. Critical assessment of metagenome interpretation: the second round of challenges. Nature methods 2022;19(4):429–440.
33. Soiland-Reyes S, Sefton P, Crosas M, Castro LJ, Coppens F, Fernández JM, et al. Packaging research artefacts with RO-Crate. Data Science 2021;(Preprint):1–42.
34. Wilkinson MD, Dumontier M, Aalbersberg IJ, Appleton G, Axton M, Baak A, et al. The FAIR Guiding Principles for scientific data management and stewardship. Scientific data 2016;3(1):1–9.
35. Wei Q, Khan IK, Ding Z, Yerneni S, Kihara D. NaviGO: interactive tool for visualization and functional similarity and coherence analysis with gene ontology. Bmc Bioinformatics 2017;18(1):1–13.
36. Amstutz P, Crusoe MR, Tijanić N, Chapman B, Chilton J, Heuer M, et al., Common workflow language, v1. 0. figshare; 2016.
37. Burgin J, Ahamed A, Cummins C, Devraj R, Gueye K, Gupta D, et al. The European Nucleotide Archive in 2022. Nucleic Acids Research 2023;51(D1):D121–D125.
38. Microbiome Informatics ENA fetch tool. MGnify; 2022. https://github.com/EBI-Metagenomics/fetch_tool, originaldate: 2018-09-06T15:38:50Z.
39. Chen S, Zhou Y, Chen Y, Gu J. fastp: an ultra-fast all-in-one FASTQ preprocessor. Bioinformatics 2018;34(17):i884–i890.
40. Nawrocki EP, Eddy SR. Infernal 1.1: 100-fold faster RNA homology searches. Bioinformatics 2013;29(22):2933–2935.
41. Matias Rodrigues JF, Schmidt TS, Tackmann J, von Mering C. MAPseq: highly efficient k-mer search with confidence estimates, for rRNA sequence analysis. Bioinformatics 2017;33(23):3808–3810.
42. Milanese A, Mende DR, Paoli L, Salazar G, Ruscheweyh HJ, Cuenca M, et al. Microbial abundance, activity and population genomic profiling with mOTUs2. Nature communications 2019;10(1):1–11.
43. Vollmers J, Wiegand S, Kaster AK. Comparing and evaluating metagenome assembly tools from a microbiologist's perspective-not only size matters! PloS one 2017;12(1):e0169662.
44. Li D, Liu CM, Luo R, Sadakane K, Lam TW. MEGAHIT: an ultra-fast single-node solution for large and complex metagenomics assembly via succinct de Bruijn graph. Bioinformatics 2015;31(10):1674–1676.
45. Nurk S, Meleshko D, Korobeynikov A, Pevzner PA. metaSPAdes: a new versatile metagenomic assembler. Genome Research 2017;27(5):824–834. http://genome.cshlp.org/content/27/5/824.abstract.
46. Rho M, Tang H, Ye Y. FragGeneScan: predicting genes in short and error-prone reads. Nucleic acids research 2010;38(20):e191–e191.
47. Jones P, Binns D, Chang HY, Fraser M, Li W, McAnulla C, et al. InterProScan 5: genome-scale protein function classification. Bioinformatics 2014;30(9):1236–1240.
48. Mitchell AL, Attwood TK, Babbitt PC, Blum M, Bork P, Bridge A, et al. InterPro in 2019: improving coverage, classification and access to protein sequence annotations. Nucleic acids research 2019;47(D1):D351–D360.
49. El-Gebali S, Mistry J, Bateman A, Eddy SR, Luciani A, Potter SC, et al. The Pfam protein families database in 2019. Nucleic acids research 2019;47(D1):D427–D432.
50. Haft DH, Selengut JD, Richter RA, Harkins D, Basu MK, Beck E. TIGRFAMs and genome properties in 2013. Nucleic acids research 2012;41(D1):D387–D395.
51. Sigrist CJ, De Castro E, Cerutti L, Cuche BA, Hulo N, Bridge A, et al. New and continuing developments at PROSITE. Nucleic acids research 2012;41(D1):D344–D347.
52. Ashburner M, Ball CA, Blake JA, Botstein D, Butler H, Cherry JM, et al. Gene ontology: tool for the unification of biology. Nature genetics 2000;25(1):25–29.
53. Huerta-Cepas J, Szklarczyk D, Heller D, Hernández-Plaza A, Forslund SK, Cook H, et al. eggNOG 5.0: a hierarchical, functionally and phylogenetically annotated orthology resource based on 5090 organisms and 2502 viruses. Nucleic acids research 2019;47(D1):D309–D314.
54. Cantalapiedra CP, Hernández-Plaza A, Letunic I, Bork P, Huerta-Cepas J. eggNOG-mapper v2: functional annotation, orthology assignments, and domain prediction at the metagenomic scale. Molecular biology and evolution 2021;38(12):5825–5829.
55. Eddy SR. Accelerated profile HMM searches. PLoS computational biology 2011;7(10):e1002195.
56. Aramaki T, Blanc-Mathieu R, Endo H, Ohkubo K, Kanehisa M, Goto S, et al. KofamKOALA: KEGG Ortholog assignment based on profile HMM and adaptive score threshold. Bioinformatics 2020;36(7):2251–2252.
57. Kanehisa M, Sato Y, Kawashima M, Furumichi M, Tanabe M. KEGG as a reference resource for gene and protein annotation. Nucleic acids research 2016;44(D1):D457–D462.
58. Soiland-Reyes S, Sefton P, Crosas M, Castro LJ, Coppens F, Fernández JM, et al. Packaging research artefacts with RO-Crate. Data Science 2022;5(2):97–138.
59. De Geest P, Droesbeke B, Eguinoa I, Gaignard A, Huber S, Leo S, et al., ro-crate-py. Zenodo; 2022. https://doi.org/10.5281/zenodo.6594974, cite as.
60. Santi I, Casotti R, Comtet T, Cunliffe M, Koulouri PY, Macheriotou L, et al. European Marine Omics Biodiversity Observation Network (EMO BON) Handbook (Version 1.0). EMBRC-ERIC; 2021.
61. Pesant S, Not F, Picheral M, Kandels-Lewis S, Le Bescot N, Gorsky G, et al. Open science resources for the discovery and analysis of Tara Oceans data. Scientific data 2015;2(1):1–16.
62. Quince C, Walker AW, Simpson JT, Loman NJ, Segata N. Shotgun metagenomics, from sampling to analysis. Nature biotechnology 2017;35(9):833–844.
63. Faust K, Sathirapongsasuti JF, Izard J, Segata N, Gevers D, Raes J, et al. Microbial co-occurrence relationships in the human microbiome. PLoS computational biology 2012;8(7):e1002606.
64. Friedman J, Alm EJ. Inferring correlation networks from genomic survey data. PLoS computational biology

2012;8(9):e1002687.
65. Bharti R, Grimm DG. Current challenges and best-practice protocols for microbiome analysis. Briefings in bioinformatics 2021;22(1):178–193.
66. Reimer LC, Sardà Carbasse J, Koblitz J, Ebeling C, Podstawka A, Overmann J. Bac Dive in 2022: the knowledge base for standardized bacterial and archaeal data. Nucleic Acids Research 2022;50(D1):D741–D746.
67. Zafeiropoulos H, Paragkamian S, Ninidakis S, Pavlopoulos GA, Jensen LJ, Pafilis E. PREGO: a literature and data-mining resource to associate microorganisms, biological processes, and environment types. Microorganisms 2022;10(2):293.
68. contributors TUoMUaB, Pitfalls and limitations · BioExcel Best Practice Guide: Creating workflows with Common Workflow Language; 2021. http://docs.bioexcel.eu/cwl-best-practice-guide/limitations.html.
69. Vivian J, Rao AA, Nothaft FA, Ketchum C, Armstrong J, Novak A, et al. Toil enables reproducible, open source, big biomedical data analyses. Nature biotechnology 2017;35(4):314–316.
70. Krakau S, Straub D, Gourlé H, Gabernet G, Nahnsen S. nf-core/mag: a best-practice pipeline for metagenome hybrid assembly and binning. NAR Genomics and Bioinformatics 2022;4(1):lqac007.

point by point response to reviewers Click here to access/download;Manuscript;metaGOflow point-by-point_ revision1.docx

GIGA-D-23-00127
metaGOflow: a workflow for the analysis of marine Genomic Observatories shotgun metagenomics data
Haris Zafeiropoulos; Martin Beracochea; Stelios Ninidakis; Katrina Exter; Antonis Potirakis; Gianluca De Moro; Lorna Richardson; Erwan Corre; João Machado; Evangelos Pafilis; Ioulia Santi; Georgios Kotoulas; Robert Daniel Finn; Cymon Cox; Christina Pavloudi
GigaScience

Dear Dr Zafeiropoulos,

Your manuscript "metaGOflow: a workflow for the analysis of marine Genomic Observatories shotgun metagenomics data" (GIGA-D-23-00127) has been assessed by our reviewers. Although it is of interest, we are unable to consider it for publication in its current form. The reviewers have raised a number of points which we believe would improve the manuscript and may allow a revised version to be published in GigaScience.

In particular, Reviewer #1 highlights provides some good feedback to make the workflow more portable - There is still significant overhead of additional dependencies required to be set up on the server running the workflow components, instead of having all but the CWL runner dockerized for maximum portability. The size of a database is another hurdle, however the fact it is all packaged into a single "installation" script helps ease its use. Scientific description of inputs, results and further use of the data is not included in the manuscript.

Their reports, together with any other comments, are below. Please also take a moment to check our website at https://www.editorialmanager.com/giga/ for any additional comments that were saved as attachments.

In addition, please register any new software application in the bio.tools and SciCrunch.org databases to receive RRID (Research Resource Identification Initiative ID) and biotoolsID identifiers, and include these in your manuscript. Computational workflows should be registered in workflowhub.eu and the DOIs cited in the relevant places in the manuscript. These will facilitate tracking, reproducibility and re-use of your tool.

If you are able to fully address these points, we would encourage you to submit a revised manuscript to GigaScience. Once you have made the necessary corrections, please submit online at:

https://www.editorialmanager.com/giga/

If you have forgotten your username or password please use the "Send Login Details" link to get your login information. For security reasons, your password will be reset.

Please include a point-by-point within the 'Response to Reviewers' box in the submission system. Please ensure you describe additional experiments that were carried out and include a detailed rebuttal of any criticisms or requested revisions that you disagreed with. Please also ensure that your revised manuscript conforms to the journal style, which can be found in the Instructions for Authors on the journal homepage. If the data and code has been modified in the revision process please be sure to update the public versions of this too.

The due date for submitting the revised version of your article is 08 Sep 2023.

I look forward to receiving your revised manuscript soon.

Best wishes,
Nicole Nogoy, Ph.D
GigaScience

Dear Editor,

We would like to kindly thank both you and the reviewers for the time you spent to thoroughly read our manuscript. We sincerely appreciate all accurate comments made by both reviewers, which helped the improvement of our manuscript.
In this revised version of our manuscript, we have addressed the reviewers' comments and suggestions and, where necessary, we have incorporated changes and made amendments and alterations to the manuscript and the source code of metaGOflow.
The changes and the new sections of the manuscript are written in blue. Below we cite our detailed answers (in blue) to the editor and reviewers' comments and suggestions (black).
Further, we have added metaGOflow in the resources mentioned and you may find an entry of it in bio.tools (biotools:metagoflow) and in SciCrunch (SCR_023674); both are now included in our revised manuscript.
Regarding the further portability of metaGOflow, we have now done our best to minimise the dependencies and we provide a thorough explanation in Issues 1.1 and 1.4 on why we still need a `conda` environment. We were able to decrease the database size at ~160GB. Last, with respect to the scientific description of input - output files, we have made several clarifications and added a part in this revised version of our manuscript.
In this revised version of our manuscript, there is also a table with the features of the pipelines which are more similar to metaGOflow as a qualitative comparison and a clarification regarding the input/output files description.

Reviewer reports:

Reviewer #1:
The submitted manuscript describes a bioinformatics workflow called metaGOflow. It is billed as a user-friendly flexible workflow that can be broadly used for one-sample-at-a-time analysis of shotgun metagenomics data. The pipeline is essentially a wrapper of existing metagenomic sequencing data tools packaged into a portable workflow and uses data standards such as RO-crates for organizing sample outputs. The resulting workflow is supposed to be portable and the analysis results comparable when it is run at different research centers. This is an important development: making pipelines portable and usable by others is important for the field.

Issue #1.1
Unfortunately, the current implementation of metaGOflow is not as portable as one we would have liked. There is still significant overhead of additional dependencies required to be set up on the server running the workflow components, instead of having all but the CWL runner dockerized for maximum portability.
In this revised version of our manuscript, we have limited the dependencies to the minimum ensuring that all dependencies metaGOflow are, in fact, dependencies of `cwl`. However, regarding portability, it should be noted that metaGOflow is not aimed for a desktop deployment. Rather, it requires computational resources that are only likely to be found in an HPC environment administered by professional systems administrators. So it is not meant to be "portable" in the sense of being available to use for all with the minimum amount of deployment or resources. As such, we can expect metaGOflow to be deployed to an infrastructure where requiring hundreds of GB of resources is not an issue, and nor is installing a `conda` environment through a YAML config. The issue of portability and containerization is also addressed below in response to Issue 1.4.

Issue #1.2

The size of a database is another hurdle, however the fact it is all packaged into a single "installation" script helps ease its use.
The size of the datasets required for the quality of analyses performed by metaGOflow are necessarily large, but unlikely to pose a problem for anyone with the computational means to conduct such analyses. In addition, researchers with access to high-performance computing (HPC) facilities should have sufficient computational and storage resources to handle the database requirements. Also, please see response to Issue #1.1.

Issue #1.3
Scientific description of inputs, results and further use of the data is not included in the manuscript.
Descriptions of input and output files were not included in the initial manuscript as we prefer having a shorter manuscript, keeping this information in other locations and providing links to it. Specifically, in the initial manuscript we indicated that the description of the output files are part of the RO-Crate built by the pipeline. An example of this was provided in the use case stored in the Zenodo repo, see `ro-crate-metadata.json` file. An example of this json file is also available through the metaGOflow website for the TARA OCEAN sample.
In this revised version, we have added Figure 3 with an excerpt of the `ro-crate-metadata.json` file to display this feature. Links were added to the manuscript pointing directly to these files and a sentence was added to make clear that the description with the input, output files are available through the `ro-crate-metadata.json` file.
In addition, since the manuscript is not a research article, we kept the scientific description of results to a minimum.

Issue #1.4
Overall, this manuscript is of interest. and if the pipeline is properly implemented it and the paper will be of great use for the scientific community.
However, the following issues need to be addressed for publication:
There is still too much overhead with respect to deployment for this workflow to be considered portable, so we suggest the following adjustments to increase ease of use towards greater adoption of the workflow:

- Remove any root access requirements for installation
- Dockerize all dependencies used by the tools so that all a user needs to install to run the workflow is the workflow runner (CWL) and docker. Currently, installation of the following tools is required:
    - conda and a supporting environment
    - cwlref-runner
    - cwltool[all]
    - Rdflib-jsonld
    - rocrate
    - pyyaml

Common Workflow Language (CWL) relies on a functional environment to execute the pipeline. In the case of metaGOFlow, we offer instructions on installing the minimum required software components for its execution. This includes a CWL run engine like Toil, as well as Docker or Singularity to run the workflow steps within containers. In terms of portability, this setup is close to the minimum requirements (Gruening et al 2019; doi: 10.12688/f1000research.15140.2). These container technologies are the most widely used in bioinformatics, with Docker being the one most often used in researchers' local development environments and Singularity in HPC clusters. They are widely used, and most HPC clusters use them; however, we mention them in our installation instructions to be thorough.
Other Workflow Management Systems have their own minimum run requirements. For instance, Nextflow typically requires Java, while Snakemake requires Python along with the use of a virtual environment or conda to manage the necessary dependencies.

For metaGOflow, we use `conda` that is a commonly used package manager and environment management system. More specifically, the `Rdflib-jsonld` and the `pyyaml` libraries are the only dependencies that need to be set besides the `cwltool` engine and the containerization technology to be used and they are both related to the `cwltool` performance. Building a `conda` environment for the system dependencies of the workflow, is a common practice in such approaches (see for example the recent study of Fellows Yates et al. 2021; doi: 10.7717/peerj.10947).
In terms of other libraries required for metaGOflow, such as rocrate, they are required to generate the RO-Crate data products. In order to improve the portability we have created a container for this step, and now it can be run using Docker or Singularity.
Regarding the root user privileges mentioned, we have edited our installation instructions so the `node.js` is set as part of the `conda` environment; this javascript runtime is required by the CWL execution engines.

Issue #1.5
Results from shotgun metagenomic samples from various environments should be added to the results tables in order to cover a broader range of abundance profiles.
To address the reviewer's suggestions in issues #1.5 and #1.6, we used metaGOflow for the analysis of two more samples: one coming from a fish (*Salmo salar*) gut (ERR4765907), sequenced with the BGISEQ-500 platform, and one from a human gut sample (SRR9654976; Rampelli et al. 2020; doi: 10.1128/msystems.00124-20), sequenced with the NextSeq 550 platform. We have added the metaGOflow results for those samples, i.e., their corresponding RO-Crates, in a new version of our Zenodo repo (https://zenodo.org/record/8046421).
In the following table, it is shown that metaGOflow is in line with the "low diversity" characterisation of the fish gut as mentioned in the original study where the sample ERR4765907 derived from (Rasmussen et al. 2021; doi: 10.1038/s42003-021-02105-1).

Data product	EMO BON sediment	EMO BON water	Tara Oceans water	fish gut	human gut
total reads (M)	51.8	44	36.5	4.3	12.7
filtered reads (M)	33.2	28.2	19.9	1.44	7.8
SSU	438	361	345	71	268
LSU	719	469	444	75	308
contigs	348405	338467	102343	2476	75624
Reads with predicted CDS (M)	32.4	27.4	18.8	1.3	7.7
Pred. CDS* with IPS match (M)	9.9	9.4	5.2	0.2	2.2
Pred. CDS with GO match (M)	5.4	5.6	3.2	0.14	1.26
Pred. CDS with Pfam match (M)	9.3	8.9	4.9	0.19	2.01
Pred. CDS with KO match (M)	1	1.15	0.5	0.01	0.22

In addition, from the taxonomy profiles returned for those 2 samples, it becomes clear what we discuss in #Issue 1.6 regarding the presence of non-microbial reads.

In our opinion, adding this information in the main manuscript would not further support our main argument that metaGOflow is capable of addressing the challenges of the EMO BON data. Additionally, it

could create confusion to the readers, given the clear marine scope that the manuscript has been built on. Therefore, we provide it as supplementary material (Table S1).

Issue #1.6

Discussion of applicability of the pipeline to other non-marine data types would be useful. For example, can it be used for human microbiome data or soil data? Does anything (e.g. the database) need to be changed for this?

There is no limitation at all in using metaGOflow directly for the analysis of samples from other environments than marine (see response to Issue #1.5). Both the software and the databases used are resources aiming at the global microbial diversity. However, in cases such as of host microbiomes, the user should make sure that the host-related reads are removed from the raw sequencing data. For example, in the analyses performed in this revised version of a fish and a human gut samples, taxonomic hits of the host species themselves were recorded in the SSU and LSU tables, as well as other eukaryotic taxa. Accordingly, reads from such taxa definitely affect the functional annotation. In the case of the fish gut sample, the reads that were uploaded to ENA are named as "host_removed" by the submitters, although no such methodology is mentioned in the actual manuscript. However, host reads are still found in the results of metaGOflow. Thus, it is of high importance to remove contaminants/non-target reads from the samples, e.g., reads belonging to the host organism in host-associated samples, if the scientific interest is only in its microbiota,. In general, the user needs to always keep in mind what is the question to be addressed and what biological entities the data reflect. These might lead to study-specific bioinformatics analysis. In this revised version of our manuscript we have added a paragraph to make clear the potential use of metaGOflow for non-marine samples.

Issue #1.7

Need to have at least some comparison with the alternative pipelines for processing this data on both speed, logic, and scientific outputs.

In this revised version, a table has been added (Table 2) with the qualitative features of state-of-the-art shotgun metagenomics pipelines. However, we do not consider fair arguing that metaGOflow is faster than other workflows. It is our belief that a workflow is only as fast as the tools it invokes; besides that, speed is affected by the workflow manager that is employed. Our main argument about metaGOflow is that it is capable of dealing with the EMO BON (and similar) data and in our opinion, this is shown by the stats on Table 1. Please see also response to Issue #1.5.

Issue #1.8

A brief discussion of input data from a molecular biology standpoint will be useful: how are the libraries for sequencing prepared? What kit? Does it matter?

Library preparation and sequencing platforms are not really an issue for the effectiveness and applicability of the workflow. The EMO BON libraries are sequenced using NovaSeq, but as you can see in Issue #1.5, metaGOflow can also analyse data produced by other platforms. Regarding library preparation for the EMO BON samples, as mentioned in the EMO BON handbook (Santi et al. 2021; doi: 10.25607/OBP-1653) and the EMO BON paper (Santi et al. 2023; doi: 10.3389/fmars.2023.1118120), DNA extraction, cleaning, library preparation and sequencing is performed at a centralised facility (Genoscope) to reduce biases and ensure consistency in the high-quality of sequencing. DNA extraction is performed using commercially available kits, to minimise extraction deviations among samples. This information has also been added to the manuscript.

Issue #1.9

The description of outputs from the scientific standpoint is absent in the manuscript. It is not clear what results are produced, how to tell if the quality of data is good, what files are produced by the pipeline, and how to interpret the results. This is also not present in the wiki: just including the directory structure of the outputs without any description is not conducive to the use of the pipeline by anybody other than the developers.
As mentioned in response to #Issue 1.3, the description of the output files are included in the `ro-crate-metadata.json` file (see the edit-ro-crate.py which is used to create the RO-crate object). We have also added these descriptions in the workflow's repository wiki. In addition, the thorough description and the continuous improvement of the input/output files descriptions are under a continuous integration from the VLIZ Data Centre division.
However, with our manuscript we are not aiming to describe what metagenomics is, or how it's used, or how it's interpreted. Rather, we are expecting those who would be interested in using metaGOflow to be already conversant in metagenomics, at least at a certain level.

Issue #1.10
It would be nice to produce some kind of standardized report with key results e.g. in the form of html that can be interpreted by biologists.
The RO-Crate includes an HTML file with visualisations that are well-suited for visually exploring the data. More specifically, the output of the sequence pre-processing is provided as an .html report (`fastp.html`); the same applies for the taxonomic profiles based on the Large Sub Unit (`LSU > krona.html`) and the Small Sub Unit (`SSU > krona.html`). For the functional annotation step, a great number of software is nowadays available for their visualisation; most of those are also available as web services (e.g., ShinyGO, DICE, KOBAS-i and more). To facilitate the interpretation of the results, we also provide a summarised version of Gene Ontology, for visualisation via a specialised GO Slim developed for metagenomic data (available at http://www.geneontology.org/ontology/subsets/goslim_metagenomics.obo) like in all the MGnify pipeline versions so far.

Issue #1.11
It is assumed that processing individual samples with metaGOflow is supposed to be followed by differential analysis. No script or even discussion for this is provided but are needed to make the pipeline useful.
If by differential analysis the reviewer implies something like LefSe or DESeq, there is no reason why they cannot be performed with the final outputs of metaGOflow. However, the downstream analysis of samples analysed with metaGOflow is out of scope for this study. The final outputs of metaGOflow are available to the researchers and they can process them as they see fit. There is a great number of studies describing how data products such as those returned by metaGOflow should be analysed further. We briefly described such analyses in a new paragraph in this revised version of our manuscript.

Minor comments:

Issue #1.12
Comparison of results is not addressed and is perhaps out of scope for this publication, however we suggest commenting on how metaGOflow output can interface with potential downstream applications such as differential abundance analyses.
As mentioned in response to Issue #1.11, the downstream analysis and the comparison of several samples analysed with metaGOflow is not in the main scope of this manuscript. However, in this revised version of our manuscript, we do have added a few comments on how this can be performed.

Issue #1.13

The following remark in the Introduction is perhaps a bit overblown. Analyzing 540 microbiome samples should not exceed the computing capacity of a research institute.
"The computing requirements for the analysis of the EMO BON data exceed the computing capacity that a single research institute or even a regional High Performance Computing (HPC) system can support using the available workflows."
We agree with the reviewer that this sentence was an overstatement. We have rephrased the sentence to make our argument clear.

Issue #1.14
Spelling: Implementation > Overview > second paragraph > "metaGOlfow"
Corrected as mentioned.

Issue #1.15
Spelling: Implementation > Step 5: Functional annotation of the reads > "ProSite patterns and prorfiles"
Corrected as mentioned.

As part of your review, please consider the following:
Does the manuscript adhere to the journal's guidelines on minimum standards of reporting?
Key questions we ask reviewers to carefully check are:

1) Are the methods appropriate to the aims of the study, are they well described, and are necessary controls included?

The aim of the study is to present a standardized workflow for shotgun metagenomic data, which is indeed well described for single samples. However, the only sample results presented in the study are a comparison of computational resources at various steps in the workflow. The tools used in the workflow are simply cited, and their accuracy not explicitly assessed or even addressed, though that is likely out of this study's scope. It would be beneficial to see the results produced by shotgun metagenomic samples from various environments that cover a range of abundance profiles (https://www.sigmaaldrich.com/US/en/technical-documents/technical-article/genomics/next-gen-sequencing/metagenomic-research-into-microbiome-communities) in order to better assess metaGOflow for applications outside of marine environments.
We would like to thank the reviewer for their remark. It is a very interesting topic the validation of the tools invoked for the different steps of the analysis. However, as mentioned by the reviewer, this is out of scope for our study. The tools were selected based on their establishment/usage in the community; in most cases, they are tools built specifically for the resources they exploit, like for example InterProScan. As mentioned in our reply in response to Issue #1.5 and response to Issue #1.7, in this revised version of our manuscript, the analysis of 2 extra samples has been included (see Supplementary Table 1) along with their comparison to their initial analysis.

2) Are the conclusions adequately supported by the data shown?

Yes, their conclusion that their workflow is well suited to support EMO BOM environmental studies is supported by the results in Table 1, assuming that these samples are representative. Additionally, they rightly claim that modern workflow managers are a useful tool for large genomic studies that require rigorous standardization of analysis protocols.
We would like to thank the reviewer for this and declare that the two EMO BON samples used in our initial manuscript, and also displayed in the revised one, are data products of EMO BON and they were selected as representative ones. The third sample was chosen as a representative sample from Tara Oceans,

which was a similar initiative to EMO BON, and since it is a publicly available sample, its metaGOflow results can also be publicly available through our Zenodo repository.

3) Please indicate the quality of language in the manuscript. Does it require a heavy editing for language and clarity?

The quality of language in the manuscript does not require heavy editing, and is suitable for publication.

4) Are you able to assess all statistics in the manuscript, including the appropriateness of statistical tests used?

There are no statistics used in the manuscript.

Reviewer #2: The paper reads quite well language-wise, and provides a thorough discussion of the challenges in the field of metagenomics, discusses alternative approaches, and describes the approach taken here, with a suitable level of detail.

We would like to thank the reviewer for the kind words and the useful comments.

Main comments
============

There are two things that in my view need editing though.

Issue #2.1
Firstly, the paper contains a number of sentences that appear very unclear and need editing to make sure statements are clear, concrete and backed up with examples where appropriate.
In this revised version, we have edited the manuscript in a series of cases such as those mentioned by the reviewer, in order to make our statements clear.

Issue #2.2
Secondly but closely related is that the discussion of the novel features provided by metaGOflow is quite thin. The novel features are only listed briefly in the Introductions section, and the discussion of what improvements metaGOflow brought is to me very unclear from the discussion.
For example, I think sentences like these: "metaGOflow adds on a list of similar approaches such as nf-core/mag [64], metaWRAP [19], MG-RAST [23], JGI-IMG [25], bioBakery 3 (MetaPhlan 3) [20]. metaGOflow highlights the potential that modern workflow managers and containerization technologies support for building workflows upon workflows" ... don't say very much at all, and need to be made much more concrete. For example, in what concrete way did workflows and containerization help improve the analysis?
We would like to thank the reviewer for pointing out this weak point. First, to address this issue, in this revised version of our manuscript we have added a table (Table 2) that contains a qualitative comparison of the features of several published workflows and pipelines that perform similar tasks to those of metaGOflow.
As mentioned in the original version of the manuscript, our workflow's novelty compared to the original MGnify version is: a) the possibility for partial execution and b) the incorporation of an alternative assembler. On top of that, our approach ends with the generation and verification of a Research Object (RO) crate ensuring the workflow's FAIRness and making all data products, along with details on the

employed methods, accessible in a machine-readable way; it also links the raw data's metadata to the final analysis outputs. To the best of our knowledge and as mentioned in Table 2, this is a novel feature in metagenomics bioinformatics analysis pipelines.
In addition, we have added an extra paragraph in the Discussion session to discuss Table 2 and clarify the points raised in this issue.

Issue #2.3
The rest of this last paragraph provides more information, but needs to be expanded with more concrete examples.

For example in this sentence:
"Regarding raw data deriving from GOs, metaGOflow facilitates data generation for the interpretation of biodiversity changes over time [...]"
... this sounds OK...
"[...] thus granting valuable insights to the scientific community and building a solid foundation for long-term sustainable and high-value data outputs."
Here I have questions though. In what way does interpretation over time make data products long-term sustainable? I might be missing something here as a non-expert in metagenomics, but I get the feeling that some of these statements are not connected. Perhaps I'm wrong, but I think at the very least it needs to be made more concrete.
We would like to thank the reviewer for pointing to us arguments that would not be clear to a broader audience. It is not the "interpretation over time" that makes data products "long-term sustainable". In this revised version of our manuscript, we have clarified our arguments.

Issue #2.4
Again, as said, I think the paper needs editing to make sure all sentences are clear, concrete and backed up by examples where appropriate.
We would like to thank the reviewer for pointing out points on our manuscript that needed to be more clear.

Further comments
==============

Issue #2.5
1. The figure legend for Figure 1 is way too short and needs to explain with a few sentences what is seen in the image.
Done as suggested.

Issue #2.6
2. The CDS abbreviation is not explained in Table 2.
Done as suggested.

Issue #2.7
3. CDS is missing from the abbreviations list.
The abbreviation was added as mentioned. Also, the SSU and LSU abbreviations were not included in the first version of our manuscript and have been added in the revised version.

Issue #2.8
4. It seems Node.js is a requirement that should be listed, at least as an alternative to Docker/Singularity?

Node.js is indeed required in cases where Docker is not installed; something quite common in HPC systems where Singularity is preferred. This is the case as the CWL execution engines need the node.js runtime for workflow execution, to evaluate dynamic expressions in CWL. If Docker is installed then the CWL execution engine will download and use a node.js container, otherwise the user needs to install it.
Node.js ensures the valid performance of the `InlineJavascriptRequirement` for the case of the most commonly used CWL engines, i.e. `cwltool` and `toil-cwl-runner`. Node.js is now included in the "Dependencies" section on our GitHub repository. We have now included it in the "Other requirements" entry of the "Availability of source code and requirements" in the main manuscript too.
Also, please see response to Issue #1.4.

Language fixes and typos
====================
Issue #2.9
1. Regarding this sentence on page 2:
"The computing requirements for the analysis of the EMO BON data exceed the computing capacity that a single research institute or even a regional High Performance Computing (HPC) system can support using the available workflows". It would be fitting with a rough estimate of at least the order of magnitude of computational resources needed here. TBs of RAM? hundreds of CPU hours?
As different workflows and different setups of a workflow would require different computational resources it would be hard to come up with any specific statistics. However, in our attempt to address this issue, we came across a great recent study from Meyer et al. 2022 (doi: 10.1038/s41592-022-01431-4). Based on that, some indicative range values have been added to this revised version in the relative section.

Issue #2.10
2. Typo at the end of page 2 (last paragraph):
metaGOlfow -> metaGOflow
Corrected as mentioned.

Issue #2.11
3. Sentence bug in second paragraph on page 3:
"preferably in as short a period of time possible" -->
"preferably in as short a period of time as possible"
Corrected as mentioned.

Issue #2.12
4. Sentence bug on page 3, 4th paragraph:
"metaGOflow data products are packages RO-Crates:" -->
"metaGOflow data products are packaged as RO-Crates"
Corrected as mentioned.

Issue #2.13
5. Sentence bug on page 4:
"support for fast sequence retrieval" -->
"support fast sequence retrieval"
Corrected as mentioned.

Issue #2.14
6. Sentence bug on page 5, last paragraph:

"metaGOflow adds on a list" -->
"metaGOflow adds to a list"
Corrected as mentioned.

Figure 1

Click here to access/download;Figure;eosc-life-marine-gos-pipeline.drawio.png

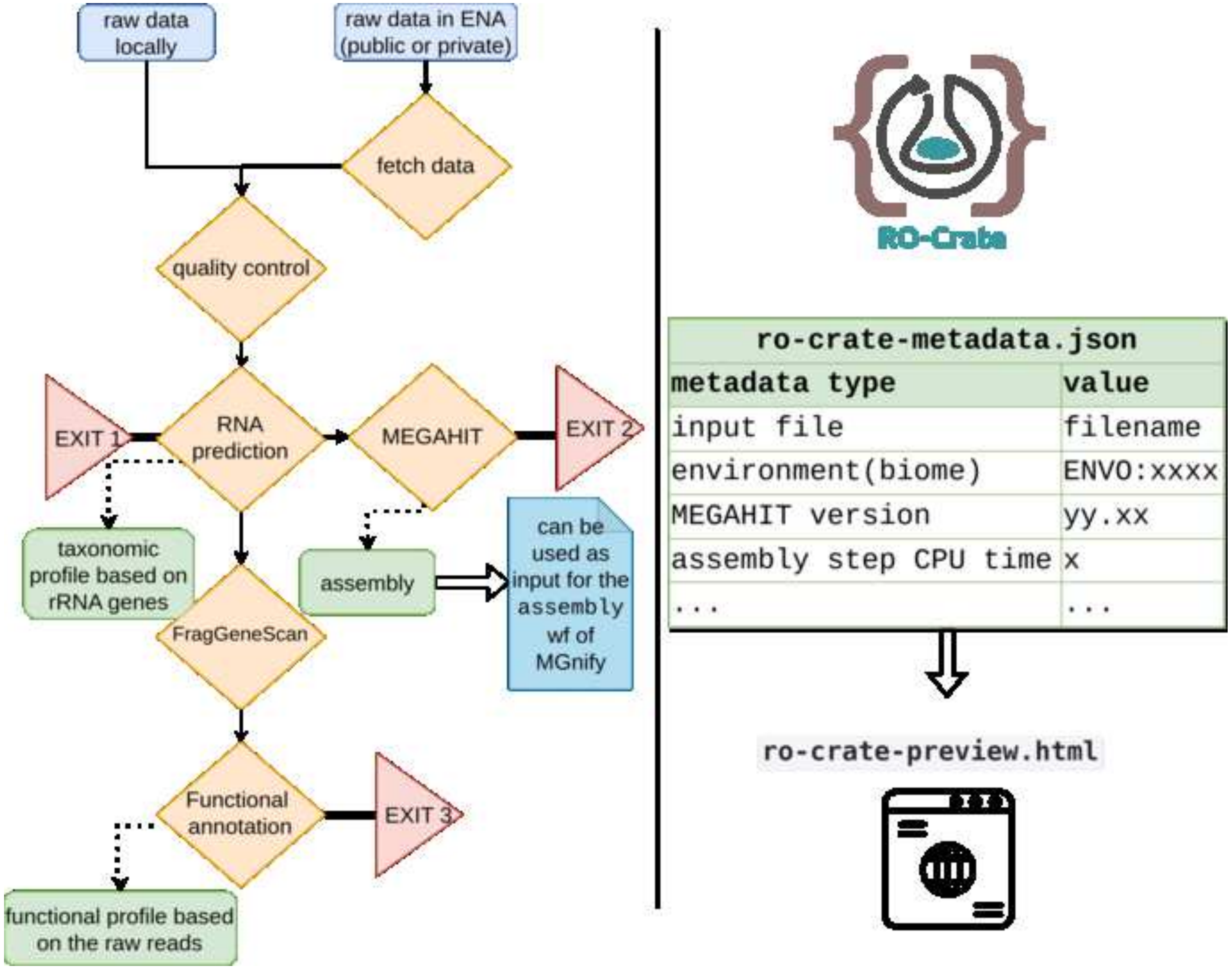

Figure 2

Click here to access/download;Figure;metagoflow-results.drawio.png

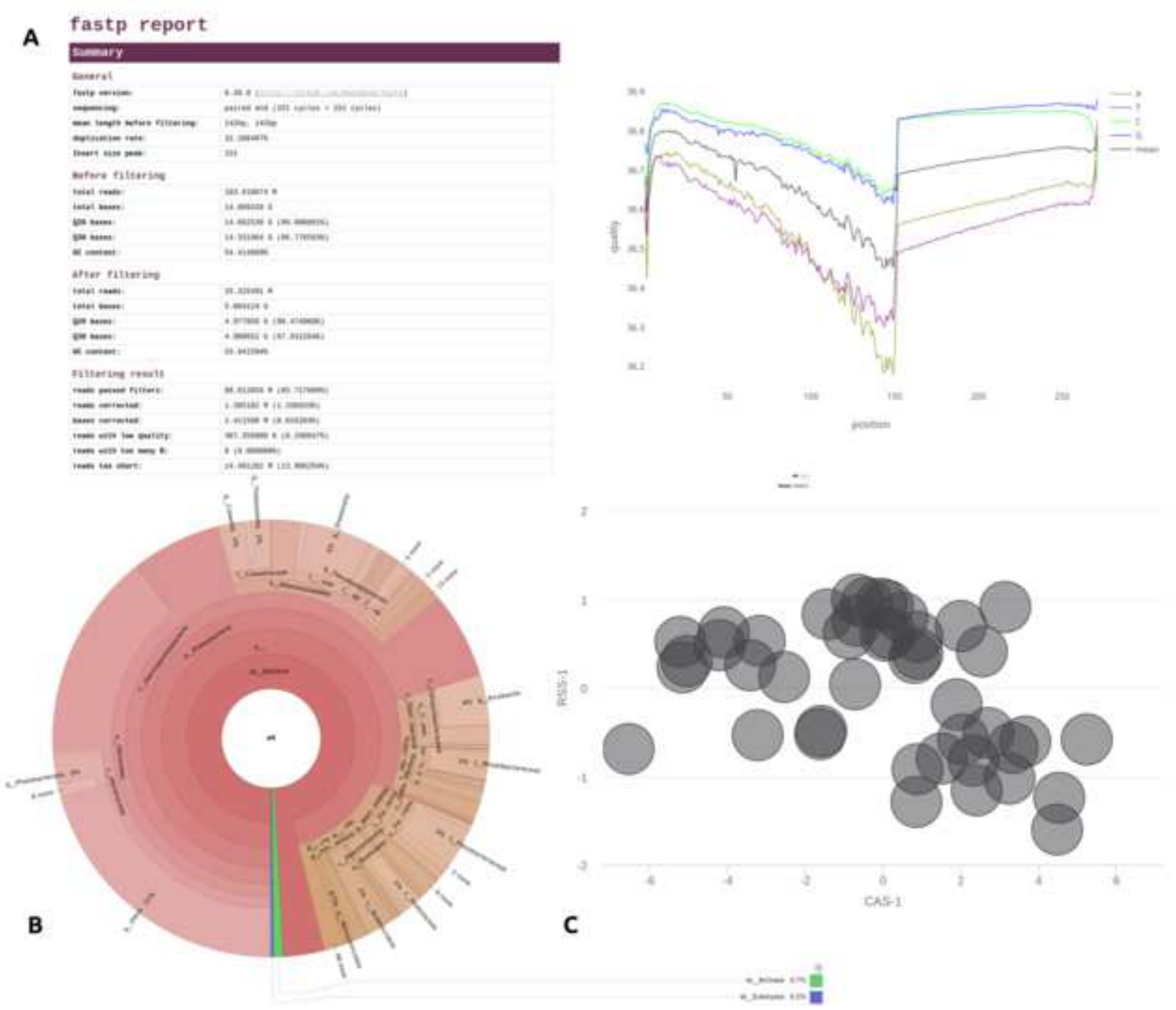

Click here to access/download;Figure;ro-crate-part-metadata.png


```
...
{
    "@id": "results/functional-annotation/stats/interproscan.stats",
    "@type": "File",
    "encodingFormat": "text/plain",
    "name": "InterProScan summary statistics"
},
{
    "@id": "results/functional-annotation/stats/go.stats",
    "@type": "File",
    "encodingFormat": "text/plain",
    "name": "Geno Ontology summary statistics"
},
{
    "@id": "results/functional-annotation/stats/ko.stats",
    "@type": "File",
    "encodingFormat": "text/plain",
    "name": "Kegg Ontology summary statistics"
},
{
    "@id": "results/functional-annotation/stats/pfam.stats",
    "@type": "File",
    "encodingFormat": "text/plain",
    "name": "Pfam summary statistcs"
},
{
    "@id": "results/functional-annotation/stats/orf.stats",
    "@type": "File",
    "encodingFormat": "text/plain",
    "name": "ORF summary statistics"
},
{
    "@id": "https://www.apache.org/licenses/LICENSE-2.0",
    "@type": "CreativeWork",
    "identifier": "https://spdx.org/licenses/Apache-2.0.html",
    "name": "Apache License 2.0"
},
```

Supplementary Table 1

Click here to access/download
Supplementary Material
metaGOflow_Supplementary-Table-1.docx
