# Supplementary material for: metaGOflow: a workflow for the analysis of marine Genomic Observatories shotgun metagenomics data
Source: Gigascience. 2023 Oct 18;12:giad078. doi: 10.1093/gigascience/giad078 (PMC10583283; doi:10.1093/gigascience/giad078)
Supplement: giad078_GIGA-D-23-00127_Revision_2 [file giad078_giga-d-23-00127_revision_2.pdf]

GigaScience

metaGOflow: a workflow for the analysis of marine Genomic Observatories shotgun metagenomics data

--Manuscript Draft--

**Manuscript Number:**	GIGA-D-23-00127R2
**Full Title:**	metaGOflow: a workflow for the analysis of marine Genomic Observatories shotgun metagenomics data
**Article Type:**	Technical Note
**Funding Information:**	HORIZON EUROPE European Research Council (824087) — Not applicable<br>European Marine Biological Resource Centre — Not applicable
**Abstract:**	Background: Genomic Observatories (GOs) are sites of long-term scientific study that undertake regular assessments of the genomic biodiversity. The European Marine Omics Biodiversity Observation Network (EMO BON) is a network of GOs that conduct regular biological community samplings to generate environmental and metagenomic data of microbial communities from designated marine stations around Europe. The development of an effective workflow is essential for the analysis of the EMO BON metagenomic data in a timely and reproducible manner.<br>Findings: Based on the established MGnify resource we developed metaGOflow; metaGOflow supports the fast inference of taxonomic profiles from GO-derived data based on rRNA genes and their functional annotation using the raw reads. Thanks to the Research Object Crate (RO-Crate) packaging, relevant metadata about the sample under study, and the details of the bioinformatics analysis it has been subjected to, are inherited to the data product while its modular implementation allows running the workflow partially. The analysis of two EMO BON and one Tara Oceans samples was performed as a use case.<br>Conclusions: metaGOflow is an efficient and robust workflow that scales to the needs of projects producing big metagenomic data such as EMO BON. It highlights how containerization technologies along with modern workflow languages and metadata package approaches can support the needs of researchers when dealing with ever-increasing volumes of biological data. Despite being initially oriented to address the needs of EMO BON, metaGOflow is a flexible and easy-to-use workflow that can be broadly used for one-sample-at-a-time analysis of shotgun metagenomics data.
**Corresponding Author:**	Haris Zafeiropoulos<br>KU Leuven Rega Institute for Medical Research.: Katholieke Universiteit Leuven Rega Institute for Medical Research<br>Leuven, BELGIUM
**Corresponding Author Secondary Information:**	
**Corresponding Author's Institution:**	KU Leuven Rega Institute for Medical Research.: Katholieke Universiteit Leuven Rega Institute for Medical Research
**Corresponding Author's Secondary Institution:**	
**First Author:**	Haris Zafeiropoulos
**First Author Secondary Information:**	
**Order of Authors:**	Haris Zafeiropoulos

Powered by Editorial Manager® and ProduXion Manager® from Aries Systems Corporation

	Martin Beracochea
	Stelios Ninidakis
	Katrina Exter
	Antonis Potirakis
	Gianluca De Moro
	Lorna Richardson
	Erwan Corre
	João Machado
	Evangelos Pafilis
	Ioulia Santi
	Georgios Kotoulas
	Robert Daniel Finn
	Cymon Cox
	Christina Pavloudi
**Order of Authors Secondary Information:**	
**Response to Reviewers:**	Please see the attached file "metaGOflow point-by-point_ revision2.docx".
**Additional Information:**	
**Question**	**Response**
Are you submitting this manuscript to a special series or article collection?	No
**Experimental design and statistics**<br><br>Full details of the experimental design and statistical methods used should be given in the Methods section, as detailed in our Minimum Standards Reporting Checklist. Information essential to interpreting the data presented should be made available in the figure legends.<br><br>Have you included all the information requested in your manuscript?	Yes
**Resources**<br><br>A description of all resources used, including antibodies, cell lines, animals and software tools, with enough information to allow them to be uniquely identified, should be included in the Methods section. Authors are strongly encouraged to cite Research Resource	Yes

Identifiers (RRIDs) for antibodies, model organisms and tools, where possible.<br><br>Have you included the information requested as detailed in our Minimum Standards Reporting Checklist?	
**Availability of data and materials**<br><br>All datasets and code on which the conclusions of the paper rely must be either included in your submission or deposited in publicly available repositories (where available and ethically appropriate), referencing such data using a unique identifier in the references and in the “Availability of Data and Materials” section of your manuscript.<br><br>Have you have met the above requirement as detailed in our Minimum Standards Reporting Checklist?	Yes

Manuscript

Click here to access/download;Manuscript;marine_GOs_CWL_workflow_rev

OXFORD (GIGA)n SCIENCE

GigaScience, 2022, 1–10

doi: xx.xxxx/xxxx
Manuscript in Preparation
Technical Note

TECHNICAL NOTE

metaGOflow: a workflow for the analysis of marine Genomic Observatories shotgun metagenomics data

Haris Zafeiropoulos[1,2 *], Martin Beracochea[3 *], Stelios Ninidakis[1], Katrina Exter[4], Antonis Potirakis[1], Gianluca De Moro[5], Lorna Richardson[3], Erwan Corre[6], João Machado[5], Evangelos Pafilis[1], Georgios Kotoulas[1], Ioulia Santi[7,1], Robert D. Finn[3], Cymon J. Cox[5] and Christina Pavloudi[1,8 †]

[1] Institute of Marine Biology, Biotechnology and Aquaculture (IMBBC), Hellenic Centre for Marine Research (HCMR), Former U.S. Base of Gournes P.O. Box 2214, 71003, Heraklion, Crete, Greece and [2] KU Leuven, Department of Microbiology, Immunology and Transplantation, Rega Institute for Medical Research, Laboratory of Molecular Bacteriology, 3000 Leuven, Belgium and [3] European Molecular Biology Laboratory, European Bioinformatics Institute (EMBL-EBI), Wellcome Genome Campus, Hinxton, Cambridge CB10 1SD, UK and [4] Flanders Marine Institute (VLIZ), Oostende, Belgium and [5] Centro de Ciências do Mar (CCMAR), Universidade do Algarve, Campus de Gambelas, 8005-139, Faro, Portugal and [6] CNRS, FR 2424, ABiMS Platform, Station Biologique de Roscoff (SBR), Roscoff, France and [7] European Marine Biological Resource Centre (EMBRC-ERIC), Paris, France and [8] Department of Biological Sciences, The George Washington University, District of Columbia, USA

[*] Corresponding authors: haris.zafeiropoulos@kuleuven.be & mbc@ebi.ac.uk
[†] Current affiliation: PSL Research University: EPHE-UPVD-CNRS, UAR CNRS 3278 Centre de Recherche Insulaire et Observatoire de l'Environnement (CRIOBE), France & Laboratoire d'Excellence "CORAIL", Centre de Recherche Insulaire et Observatoire de l'Environnement (CRIOBE), French Polynesia

Abstract

Background: Genomic Observatories (GOs) are sites of long-term scientific study that undertake regular assessments of the genomic biodiversity . The European Marine Omics Biodiversity Observation Network (EMO BON) is a network of GOs that conduct regular biological community samplings to generate environmental and metagenomic data of microbial communities from designated marine stations around Europe. The development of an effective workflow is essential for the analysis of the EMO BON metagenomic data in a timely and reproducible manner.
Findings: Based on the established MGnify resource we developed metaGOflow; metaGOflow supports the fast inference of taxonomic profiles from GO-derived data based on rRNA genes and their functional annotation using the raw reads. Thanks to the Research Object Crate (RO-Crate) packaging, relevant metadata about the sample under study, and the details of the bioinformatics analysis it has been subjected to, are inherited to the data product while its modular implementation allows running the workflow partially. The analysis of two EMO BON and one Tara Oceans samples was performed as a use case.
Conclusions: metaGOflow is an efficient and robust workflow that scales to the needs of projects producing big metagenomic data such as EMO BON. It highlights how containerization technologies along with modern workflow languages and metadata package approaches can support the needs of researchers when dealing with ever-increasing volumes of biological data. Despite being initially oriented to address the needs of EMO BON, metaGOflow is a flexible and easy-to-use workflow that can be broadly used for one-sample-at-a-time analysis of shotgun metagenomics data.

Key words: shotgun metagenomics; MGnify; Common Workflow Language (CWL); containers; provenance; RO-Crate

Compiled on: August 18, 2023.
Draft manuscript prepared by the author.

Introduction

It is well established that microbial assemblages support multiple ecosystem services and that microbial community profiling using metagenomics methods can help elucidate the mechanisms that govern the structure of these communities and their interactions with the environment [1]. The community composition and structure of marine microbiome is directly correlated with environmental quality [2, 3]. Indeed, the quality of a marine microbial environment (e.g. a marine sediment) can impact the food chain [4] through the physical and chemical effects of secondary metabolites [5]. In addition, secondary metabolites produced by microorganisms may also become targets for bio-prospecting in medicine and industry [6]. Monitoring the changes in microbial community composition and function due to climate change-related stressors, such as ocean acidification or increases in temperature and UV absorption, can provide insights on ecosystem function, health, and resilience [7].

Pioneering research programmes such as the Ocean Sampling Day (OSD) [8], Malaspina circumnavigation expedition [9], and Tara Oceans [10], have been instrumental in collecting large series' of marine genomic samples from sites around the globe. The analysis of data resulting from these studies has greatly increased our understanding of the importance, the role, and the mechanisms governing microbial communities in some of the most common, sensitive or threatened marine environments [11, 12, 13]. EMO BON [14], a European Marine Biological Resource Centre (EMBRC-ERIC) initiative, is designed to continue and expand this effort by regular bimonthly microbial genomic biodiversity samplings at designated marine coastal stations around the European coastline. In the first two years of the EMO BON (2021-2022) it is expected that more than 540 shotgun metagenomic data sets from water column and sediment samples will be generated from 17 European sites.

The ultimate success of GOs depends on the development and adoption of standards for sampling, metadata collection, sequencing, and data analysis. The provision of metadata relating to the raw sequence data, data products, and their analysis methods, are of high importance for interpretation and interoperability, and need to be accessible in both human- and machine-readable formats. Legislative framework, such as the Nagoya Protocol for Access and Benefit Sharing (ABS) [15], and community written frameworks, such as those developed by the Genomic Standards Consortium (GSC) [16], as well as initiatives encouraging adherence to best practices, such as the Better Biomolecular Ocean Practices (BeBOP) project [17], have all been key to providing agreed-upon standard that aim to fulfil these needs. Standard operating procedures and standardised methods of analysis enable the comparison of results among sites, through time, and among projects, without which, much of the value of the data for environmental assessment is lost.

Effective analysis of shotgun metagenomic data is time-consuming, especially regarding computational steps such as sequence assembly and annotation [18]. Moreover, microbial community profiling and functional analyses are most useful when samples are maximally comparable in space and time, and have been thereby treated using the same analytical procedures. To address the challenges that arise when analyzing metagenomic data, numerous workflows and pipelines have been developed. Notable pipelines include metaWRAP [19], bioBakery [20], and nf-core [21], which provides a collection of pipelines such as nf-core/ampliseq [22] and nf-core/taxprofiler [21]. Recently, containerization approaches (e.g., Docker [23], Singularity [24] etc.), along with workflow managers (e.g., Nextflow [25], Snakemake [26] etc.), have been widely used to a) address the complexity of the analysis, b) facilitate execution and reproducibility and c) distribute and share software to a broader audience [27]. nf-core and ATLAS [28] shotgun metagenomic analysis pipelines are examples of the implementation of such approaches.

Additionally, there are (data analysis) resources like MG-RAST [29], MGnify [30], and IMG/M [31] that come with their own distinct advantages and disadvantages.

The computing requirements for the analysis of the EMO BON data may exceed the computing capacity that a single research institute and/or a regional High Performance Computing (HPC) (i.e., Tier 2) systems can support using the available workflows. Indicatively, for a single dataset, software tools related to the retrieval of taxonomic profiles require up to 160 CPU hours and up to 100 GB of RAM [32]. Computing requirements for the functional annotation of shotgun reads are even higher. Nevertheless, timely provision of data and data products from GOs is of paramount importance to facilitate long-term ecological studies, to accelerate policy-making, and to directly assess the impact of anthropogenic effects on the marine environment.

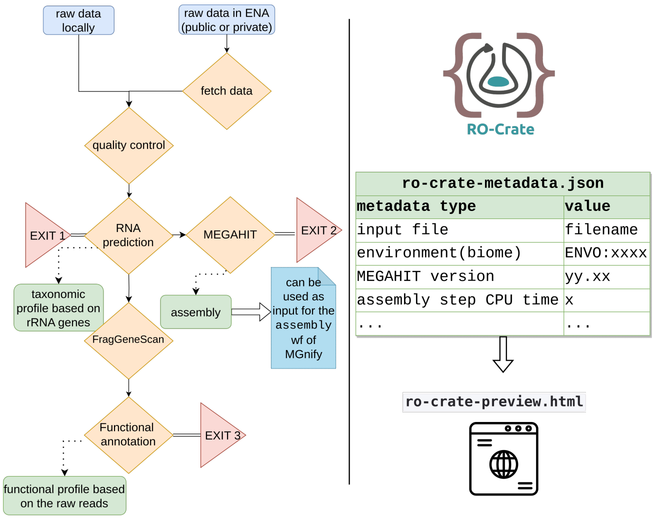

Figure 1. Schematic overview of metaGOflow, showing the main steps of the analysis along with their corresponding data products; the partial execution of the workflow is also shown by the potential exit points (left). Independent of the steps to be performed, once completed, an RO-Crate is built (right).

To address the challenges of analysing GO data in a timely and standardised framework we developed metaGOflow: a MGnify-based [30] computational workflow that implements the critical steps of a shotgun metagenomic bioinformatics analysis, and provides rich provenance metadata describing the data, data products, and workflow execution (Figure 1). The novel aspects of this workflow are mainly a) partial workflow execution; e.g. the user has the flexibility to choose whether to run the functional annotation sub-workflow or not, or even run it at a later point using the data products of the previous steps, b) the incorporation of an alternative assembler with a significantly lower computational cost as compared to the MGnfiy default one and c) the ultimate generation and verification of a Research Object (RO) crate ensuring the workflow's FAIRness. On top of that, several updates of the databases and tools invoked by MGnify have been performed.

metaGOflow consists of two basic concepts:

- an *analytical workflow* which provides taxonomic inventories and community gene function profiles of the samples as data products packaged in RO Crates [33],
- a *data provenance workflow* that generates extensive metadata and thereby provides compliance of the data, data products, and analytical procedures with FAIR data practices and the principles of Open Science, also packaged in the RO Crates [34, 17].

Implementation

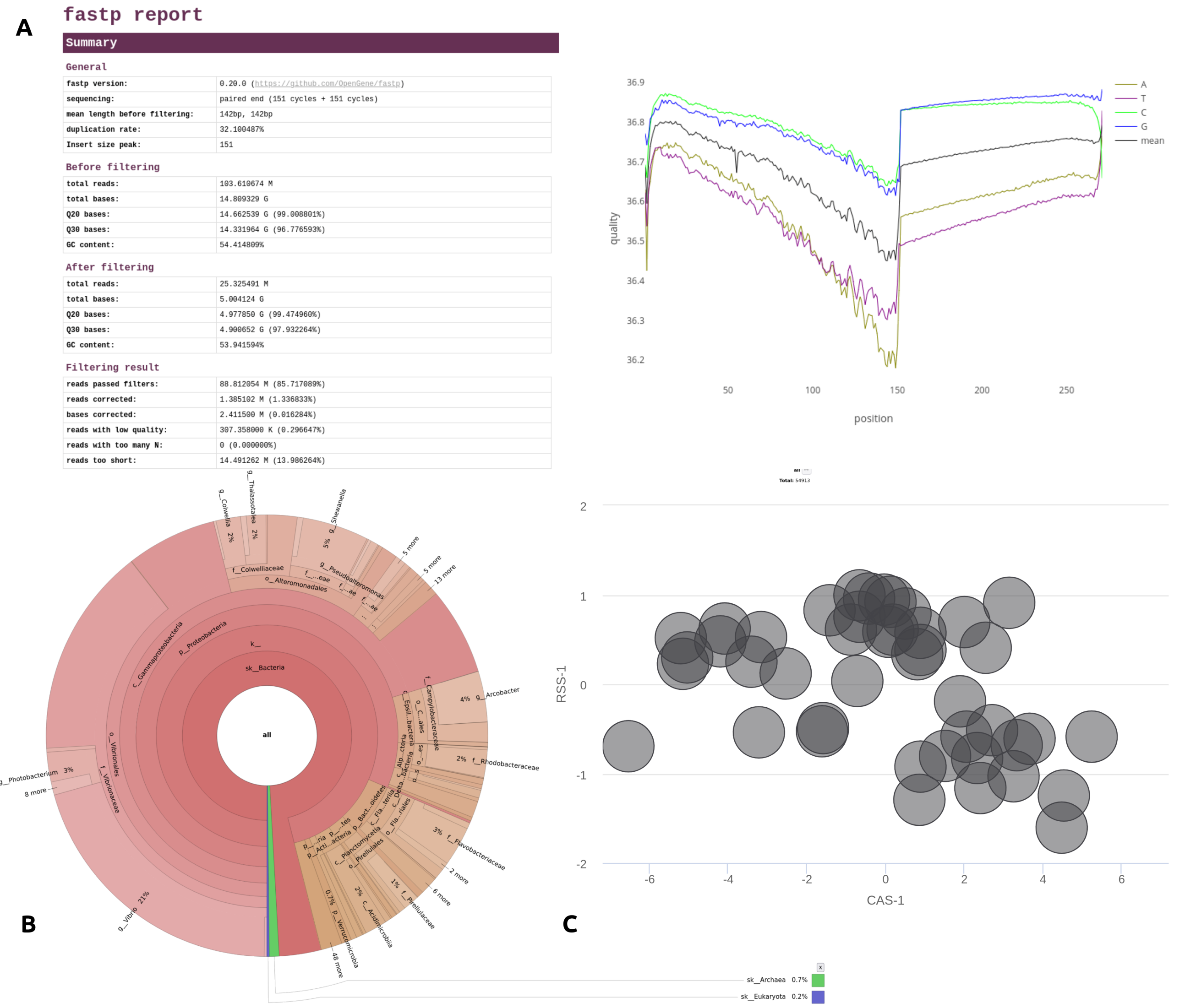

Figure 2. Visualisation of metaGOflow's main output. A. Raw data are first filtered and only high quality sequences are analysed further in the next steps. An .html file with the report of the merged reads is produced. Here, an excerpt of this report is shown: reads' statistics before and after filtering (left), ATGC chart with the quality of each base cycle-after-cycle for the merged reads (right) B. The taxonomy inventory step returns Molecular Operational Taxonomic Units (mOTUs) and the taxonomic composition based on the Large Sub Unit (LSU) and the Small Sub Unit (SSU) genes. Here, the taxonomic composition is represented by a Krona interactive visualization. C. The functional annotation step returns text files with the Gene Ontology (GO), KEGG, InterProScan, and Pfam terms retrieved. The retrieved GO terms are presented using Navigo [35], the Co-occurrence Association Score (CAS-1) and the Relevance Semantic Similarity (RSS-1). The Gene prediction step returns a `.ffn` and a `.faa` file while the assembly step a `.fasta` file including the contigs retrieved. The main output of the provenance feature is the `ro-crate-metedata.json` file.

Overview

The pillars around which metaGOflow has been built, namely containerisation technologies such as Docker [23] and Singularity [24], and the Common Workflow Language (CWL) [36], ensure the workflow's ability to perform in different HPC and cloud computing platforms, following the MGnify example.

metaGOflow inherits the architecture of MGnify pipeline-v5 and exploits several of the already containerized tools and the subworkflows implemented in the MGnify pipeline. Several enhancements and upgrades allow metaGOflow to make use of the latest versions of the tools and databases invoked. metaGOflow makes extensive use of CWL *subworkflows* and *conditional* step execution to address the specific needs of the EMO BON project from a computing resources point of view.

For example, the user can run the workflow to only generate the taxonomic inventory of a sample. Then, at a later time and by using the output of the first analysis, the user can also generate the assembly of this sample's reads and/or their functional annotation. This flexibility in the workflow is essential as there are a considerable number of samples to be analysed (preferably in as short a period of time as possible), and the computing requirements, especially for the functional annotation step, can be substantial (see Table 1).

In its current version (v.1.0.1), metaGOflow has 5 distinct steps. As in MGnify, metaGOflow analyses a single sample at a time (see Figure 1). The user may either provide locally stored raw data (`.fastq` files) or start the workflow by giving a European Nucleotide Archive (ENA)[37] run accession number. In the later case, metaGOflow invokes the `fetch_tool` [38] to retrieve the raw sequence files from ENA; if the data to be retrieved are held privately, the username and password of the associated ENA account are also requested. The user sets the steps of the workflow to be performed and provides values for certain tool parameters through a text-based configuration file (`config.yml`).

To enhance the FAIRness of the data products and of the bioinformatic analysis, metaGOflow data products are packages as RO-Crates: this allows the set of files to be semantically described, to be accompanied by the metadata that describe the precise steps of the

workflow execution, the tools and the parameters used, and to flag the specific input and output files. This description is provided in a JSON-LD file following a particular (user-generated) profile. Along with the data products, the RO-crate contains information describing the version of the workflow *per se*, including the software and database versions that it uses.

A comparison of the main features of metaGOflow with other commonly used pipelines for shotgun metagenomic analysis is given in Table 2.

metaGOflow is available on GitHub. A Continuous Integration/Continuous Deployment (CI/CD) workflow using GitHub Actions ensures the validity of the workflow's `cwl` main script and, therefore, of all its components. A thorough description of how to install and use metaGOflow, as well as common errors that might occur during the analysis of a sample can be found at its wiki page, as well as on its main documentation page. The databases to be installed before using metaGOflow, require 160GB of storage and as a rule of thumb, the user should allocate 1TB of storage to perform a metaGOflow analysis.

The development and testing of metaGOflow was performed in the IMBBC HCMR "Zorbas" HPC [27] and at the HPC facility of CCMAR. Further testing was performed on the Luxembourg national supercomputer MeluXina. The use case experiments (see Section 6) were performed in a "fat" node of the "Zorbas" HPC (2x Inter(R) Xeon(R) Gold 6230 CPU @ 2.10GHz 40 cores and 500 GB).

Step 1: Sequence preprocessing

Sequences are filtered and merged using `fastp` (version 0.20.0) [39]. Short, low quality, and non-merging sequences are removed and a series of statistical tests describing the quality of the sequencing are performed. An `.html` file returned by the `fastp` tool, provides visualizations of these statistics (see Figure 2A). The filtered sequences and the merged filtered sequences are returned as `.fasta` files.

Step 2: Taxonomy inventory

metaGOflow makes use of the `esl-sfetch` miniapp of the EASEL library (S.R. Eddy, unpublished) to index the filtered sequences and support fast sequence retrieval. Then `cmsearch`, an Infernal program [40], is performed using the ribosomal and the non-coding RNA (ncRNA) Rfam covariance models (CM) (version v13.0) against the filtered sequences. Eventually, this is followed by taxonomic classification using `MAPseq` (v1.2.3) [41] and the SILVA database (version 132) for the taxonomic classification of the SSU and the LSU sequences, while `mOTUs2` [42] quantifies both known and unknown taxa on the filtered sequences. metaGOflow automatically returns Krona plots (an interactive visualization approach of hierarchical data as multi-layered pie charts [43]) using the taxonomic assignments made for the SSU and LSU genes (see Figure 2B).

Step 3: Assembly

Shotgun metagenomic read assembly requires significant computing resources as discussed in Mitchell et. *al* [30] and in Vollmers et. *al* [44]. The extent of the computational "burden" depends heavily on the chosen algorithm. To be able to handle the vast amount of data produced by EMO BON in a timely manner, and since we aim more at unravelling biodiversity at the community, rather than at the individual (i.e. species), level, metaGOflow makes use of the MEGAHIT algorithm [45]. Longer contigs would be returned if e.g. metaSPAdes [46] was employed, but given metaGOflow's high pace data generation and analysis needs, the MEGAHIT algorithm seems a better match.

Step 4: Gene prediction on the reads

metaGOflow performs gene prediction using FragGeneScan (v1.20) [47] like MGnify. This step is a prerequisite for the functional annotation of the reads (Step). To partially run this step, the user needs to provide the merged filtered `.fasta` file, provided by the sequence preprocessing step.

Step 5: Functional annotation of the reads

metaGOflow focuses on the potential metabolic processes of the whole community rather than the processes of each individual species. Therefore, it performs functional annotation at the reads level. Using InterProScan (v.57-90) [48] metaGOflow annotates the reads with InterPro5 [49], PFam [50], TIGRFAM [51], ProSite patterns and profiles [52] and Gene Ontology (GO) [53] terms. Functional annotations are returned as text files. Both GO and GO Slim (available at geneontology.org) annotations are returned. EggNOG5 [54] annotation is also performed using to the eggnog-mapper (v2.1.8) [55]. Last, metaGOflow invokes the HMMER [56] tool along with the KOfam library [57] to get KEGG orthology annotations [58]. This step requires a significant amount of computing time.

For the visualization of each annotation type there is a great number of software; indicatively, in Figure 2C, the Co-occurrence Association Score (CAS) scores of the GO terms found in the sample are plotted against their Relevance Semantic Similarity (RSS) scores, which quantify the frequency of co-occurring GO terms within the gene annotations in the GOA database, as described in Navigo [35].

Building RO-Crates

An RO-Crate is created automatically by the workflow to store the data products of the aforementioned steps, along with the MetaGOflow run associated metadata (including the user set parameters, the version and the source of the workflow used). To this end, the `rocrate` Python library [59, 60] is used. As mentioned, an RO-Crate object is accompanied by a JSON-LD file (called `ro-crate-metadata.json`), part of which is shown in Figure 3, which includes the descriptions of both input and output files.

A thorough list of the metaGOflow's data products along with their descriptions can be found in the *Description of metaGOflow's data products* page of the manual. Supporting documentation, related to some of the software tools invoked by metaGOflow, is also

Table 1. Computing requirements for the analysis of a sediment and a water column EMO BON sample as well as a Tara Oceans water sample, using metaGOflow in a "fat" node of the Zorba HPC.

workflow step(s)	computational time (hours)			memory (max RAM in Gb)		
	EB sediment	EB water	TO water	EB sediment	EB water	TO water
prepr. & taxon. invent. (Steps 1&2)	14.5	12.6	26.4	4.55	4.65	4.15
assembly (Step 3)	1.6	1.22	0.4	8.8	4.38	2.7
gene calling & funct. annot. (Steps 4&5)	98.7	92.4	84.2	205.1	188.6	155.4

EB: EMO BON, TO: Tara Oceans.

Table 2. Comparison of the main features and implementation of pipelines similar to metaGOflow.

Category	Feature	MetaWRAP	ATLAS	nf-core/taxprofiler	nf-core/funcscan	metaGOflow
	Quality control	fastqc	-	fastp, falco	-	fastp
Pre-processing	Filtering	Trim Galore	BBTools	porechop, fastp, bbduk, prinseq++, Filtlong	-	fastp
	Host-read removal	bmtagger	-	Bowtie2 for short reads and minimap2 for long reads	-	-
	Taxonomy assignment of rRNA genes	-	-	-	-	mOTUs, MAPseq
Taxonomy	Taxonomic assignment of reads and/or contigs	kraken, kraken2	-	Kraken2, DIAMOND, mOTUs, MetaPhlAn3, MALT	-	-
	Taxonomic assignment of bins	TAXATOR-TK	GTDB-tk	-	-	-
	Short read assembly	metaspades and/or MEGAHIT	MEGAHIT	-	-	MEGAHIT
Assembly	Hybrid assembly	-	Yes	-	-	-
	Group-wise co-assembly	Yes	Yes	-	-	-
	Genome binning	metaBAT2, MaxBin2, CONCOCT	metabat2, maxbin2	-	-	-
BINs-MAGs	Bin refinement	Binning-refiner	-	DAS Tool	-	-
	Gene prediction	-	-	prodigal	-	FragGeneScan
Annotation	Functional annotation	prokka (using the bins)	eggNOG	-	hAMRonization, AMPcombi, comBGC.py	InterProScan, eggNOG, hmmsearch
	Ontologies	-	eggNOG	-	-	KEGG, GO, pfam, eggNOG, InterPro
	keeping track of sample's metadata	-	-	-	-	Yes
FAIR-ness	output as RO-Crate	-	-	-	-	Yes
	workflow provided through containers	-	-	-	Yes	Yes
Architecture	workflow manager	-	snakemake	nextflow	nextflow	cwl

provided to support the interpretation of the data products.

Parameters tuning

The config.yml file is the interface between the user and the pipeline. Through this file, the user sets which steps to perform, a number of parameters related to the idiosyncrasy of each experiment, as well as parameters that may affect the time efficiency of metaGOflow to a great extent (i.e., number of chunks). Further, metaGOflow supports inline arguments describing technical aspects of how to run, e.g. which containerization technology should be used. A thorough description of these parameters, as well as best practices and rules-of-thumb, are available at metaGOflow's manual on the *Arguments and parameters* page.

Use case

To demonstrate metaGOflow and its key features, the analysis of a sediment and a water column sample from EMO BON was performed. As mentioned in the EMO BON handbook [61] and the EMO BON paper [14], DNA extraction, cleaning, library preparation and sequencing is performed at a centralised facility to minimize biases and maximize consistency in sequence quality. DNA extraction is performed using commercially available kits, to minimise deviations among samples. The samples were randomly chosen from two different stations but are considered to be representative EMO BON data. Moreover, an already publicly available marine metagenome sample from the Tara Oceans expedition [62], with size (in Gb) similar to those of the EMO BON data, was also analysed. All steps of metaGOflow were performed for each of these samples and the computational time (in hours), and the maximum memory (RAM, in GB) are reported in Table 1. Additionally, to demonstrate the applicability of metaGOflow for all types of shotgun metagenomic data, it was implemented for the analysis of a fish gut and a human gut metagenomic sample. All five samples were sequenced in different platforms: NovaSeq (EMO BON), HiSeq 2000 (Tara Oceans), BGISEQ-500 (fish gut), NextSeq 550 (human gut). The metaGOflow results for the gut samples are included in the zenodo repository and the respective statistics are given in Supplementary Table 1.

Raw sequences were preprocessed using 130 bp as the minimum length of the reads and at least 30 bp of overlap for the merging step for the 2 EMO BON samples. In case of the Tara Oceans sample, a minimum length of 108 bp was used as the sequences were shorter. The pre-processing and the taxonomic inventory step lasted about from 10 to 24 hours. By allocating a computing node similar to the one used for the use case, taxonomic inventories from at least 300 metagenomes could be produced per year, based on the results from the EMO BON samples.

For the assembly step, a minimum contig length of 200 bp was used for all the samples. The assembly of the reads using the MEGAHIT algorithm was performed in less than 2 hours, while the maximum memory required was less than 10Gb which is at least one order of magnitude less than what other software, e.g. metaSPAdes, would require. The large number of contigs returned suggests one could aim for a higher minimum contig length. For example, using a minimum contig length of 500 bp for the Tara Oceans sample, the number of contigs was decreased from 102, 343 (Table 1) to 34, 426 and the required time was about 30 minutes.

The gene calling and the functional annotation steps were those requiring the most computing resources, as expected. For each of the three samples, it took about 4 days to complete these steps, with the InterProScan part being the most computationally expensive with respect to both time and memory. In order for metaGOflow to exploit the available computing resources in an optimal way, the user is strongly advised to follow the "Improving performance" instructions of InterProScan and set the relative arguments accordingly.

A summary of the metaGOflow outputs and their respective size for this use case is shown in Table 3. A visual representation of the detailed results (quality control report, taxonomic inventories, functional annotations) of the workflow can be found through this GitHub page. An example of the complete data product of metaGOflow, packed in a RO-Crate, can be found through this Zenodo repo. For the EMO BON samples, the default configuration files config.yml were used; for the Tara Oceans sample, the config.yml is included in the respective RO-crate object, which is available in the Zenodo repository.

Based on the scientific questions to be addressed, several types of downstream statistical analysis using the metaGOflow data products might be performed. Most of these statistical approaches are not specific for the analysis of metagenomic datasets *per se* [63]. Contrary, they are well established in several research communities: microbial ecologists, microbiologists, medical scientists. However, the nature of the metagenomic data lead to several challenges, such as the "compositional effect" that need to be dealt to the best possible extent [64, 65].


```
{
    "@id": "results/functional-annotation/stats/interproscan.stats",
    "@type": "File",
    "encodingFormat": "text/plain",
    "name": "InterProScan summary statistics"
},
{
    "@id": "results/functional-annotation/stats/go.stats",
    "@type": "File",
    "encodingFormat": "text/plain",
    "name": "Geno Ontology summary statistics"
},
{
    "@id": "results/functional-annotation/stats/ko.stats",
    "@type": "File",
    "encodingFormat": "text/plain",
    "name": "Kegg Ontology summary statistics"
},
{
    "@id": "results/functional-annotation/stats/pfam.stats",
    "@type": "File",
    "encodingFormat": "text/plain",
    "name": "Pfam summary statistcs"
},
{
    "@id": "results/functional-annotation/stats/orf.stats",
    "@type": "File",
    "encodingFormat": "text/plain",
    "name": "ORF summary statistics"
},
{
    "@id": "https://www.apache.org/licenses/LICENSE-2.0",
    "@type": "CreativeWork",
    "identifier": "https://spdx.org/licenses/Apache-2.0.html",
    "name": "Apache License 2.0"
},
```


Figure 3. Part of the ro-crate-metadata.json file describing the metaGOflow output files.

Discussion & conclusions

Metagenomic applications include different procedures and require expertise in different topics, from field sampling, to lab analyses, to sequencing [66]. This inevitably leads to delays in raw data production, let alone usable scientific results. On top of that, metagenomic raw data are not directly usable as they require time-consuming and computationally-demanding processing as well as specialized bioinformatics expertise [66, 63]. For EMO BON and other GOs to produce applicable and fit-for-purpose data, it is of huge importance that quality-controlled and standardised data, as well as informative data products, are made rapidly available. The disentanglement of the analyses from technical expertise and extensive computing infrastructures will allow the direct generation of meaningful data products, even by non-experts. There is a paramount added value to the provision of preliminary results and data prod-

Table 3. metaGOflow results for the two EMO BON samples (marine sediment and a water column) and the Tara Oceans (seawater) sample.

product	EMO BON sediment	EMO BON water	Tara Oceans water
total reads (M)	51.8	44.0	36.5
filtered reads (M)	33.2	28.2	19.9
SSU	438	361	345
LSU	719	469	444
contigs	348,405	338,467	102,343
Reads with predicted CDS (M)	32.4	27.4	18.8
Pred. CDS* with IPS match (M)	9.9	9.4	5.2
Pred. CDS with GO match (M)	5.4	5.6	3.2
Pred. CDS with Pfam match (M)	9.3	8.9	4.9
Pred. CDS with KO match (M)	1.0	1.15	0.5

M: millions, *CDS: Coding Sequences

ucts (i.e. taxonomic inventories) from metagenomic GO samples as it can lead to the full exploitation of the data, including enhanced and timely decision-making and successful environmental quality monitoring of the marine environment.

metaGOflow was developed with the ultimate objective to build a distributed workflow for analyses of marine metagenomic data generated by GOs such as EMO BON. The modular notion of metaGOflow allows us to perform the steps related to the taxonomy inventories and at a later point investigate the functional potential of a sample. Taxonomic inventories, essential for the case of GOs, are retrieved in a few hours. The functional annotation, as implemented, is highly time consuming compared to any other step of the workflow. That is mostly because of the InterProScan implementation; the vast amount of sequences but also the `standalone` module with which the scan is performed, lead to long single threaded processes. However, once the `clustermode` will be as fault tolerant as the `standalone`, metaGOflow will adopt it. On top of that, optimisations on the implementation of the InterProScan step would decrease further the total time for the complete analysis. MEGAHIT provides an assembly of the reads that it can then be used with the corresponding MGnify workflow for further analysis. Ultimately, using the parallel option of the `cwltool` combined with HPC environments and its modular notion, metaGOflow enables the effective, on time and valid, analysis of GOs data.

metaGOflow packages all its output, the workflow's metadata as well as the user's settings, in RO-crates, which is a novel feature in metagenomics bioinformatics analysis pipelines, to the best of our knowledge and as mentioned in Table 2. This novelty in the workflow's implementation allows the EMO BON community to access all data products, along with details on the employed methods, in a machine-readable way, either directly (see Zenodo example) or through portals such as MGnify. Thus, it is now far easier for data and data products to be re-used for meta-analyses, but also to be exploited by data integration approaches [67, 68].

CWL, i.e. the language that the workflow is built on, has certain drawbacks. Among them, the requirement for explicit input-output declarations, the fact that the Javascript `ExpressionTools` may affect the portability of the workflow, and mainly being a data-driven "dataflow", means that handy control workflow patterns (e.g., loops) cannot be used [69]. However, some other features of the language, i.e. its modularity and its consistency when combined with containerization technologies, allowed us to build on top of the well-established MGnify environment; thus, metaGOflow enables the robust, standardized and fast-enough analysis of GO data. By all means, other workflow managers, such as Nextflow [25], may also support such community efforts. Toil [70] and similar technologies will be investigated for better exploitation of the provided computing resources, as well as cloud-based implementations of the workflow. The future integration of metaGOflow in e-infrastructures will be also considered.

The need for different approaches in the analysis of the shotgun metagenomics raw data has been well established [63]. metaGOflow's data products, like the output of any bioinformatics analysis of shotgun metagenomics data [71], may be explored in various ways through a great range of downstream analysis. Questions about key taxa in a sample or in a group of samples, about essential metabolic pathways that characterize a sample or a group of samples compared with others and so on, they can now be addressed using the findings of shotgun metagenomics analysis as input. In [72], Liu et al. distinguish the possible downstream analysis in *"overall"*, exploring differences in alpha/beta- diversity and taxonomic composition in a feature table, and *"details analysis"*, identifying biomarkers via comparison (using correlation and/or network analysis, machine learning etc.).

metaGOflow adds to a list of similar approaches such as nf-core/mag [73], metaWRAP [19], MG-RAST [29], JGI-IMG [31], bioBakery 3 (MetaPhlan 3) [20]. metaGOflow highlights the potential that modern workflow managers and containerization technologies support for building workflows upon workflows. Regarding raw data deriving from GOs, metaGOflow facilitates data generation , and, subsequently, interpretation of times-series biodiversity data, thus granting valuable insights to the scientific community and building a solid foundation for long-term sustainable and high-value data outputs. Long-term sustainability is assured by the FAIRness of the outputs and the strategic support of the EMBRC-ERIC infrastructure. Moreover, even if it was initially developed to address the specific needs of a GO project such as EMO BON, metaGOflow is overall a user-friendly flexible workflow that can be broadly used for one-sample-at-a-time analysis of shotgun metagenomics data.

Availability of source code and requirements

- Project name: metaGOflow: A workflow for marine Genomic Observatories data analysis
- Project home page: https://github.com/emo-bon/MetaGOflow
- Manual page: https://metagoflow.readthedocs.io
- WorkflowHub: https://workflowhub.eu/workflows/384
- RRID: SCR_023674
- biotools id: metagoflow
- Operating system(s): Unix
- Programming language: Common Workflow Language (CWL)
- Other requirements: Docker or Singularity engines. Node.js is required in cases where Docker is not available.
- License: Apache License 2.0. For third-party components separate licenses apply. Any restrictions to use by non-academics: licence needed.

Availability of supporting data and materials

All the raw sequence files of this study are available at ENA [37]:

- EMO BON super study accession number PRJEB51688 (available at http://www.ebi.ac.uk/ena/data/view/PRJEB51688)

- EMO BON marine sediment sample: run accession number ERS14961254 (available at http://www.ebi.ac.uk/ena/data/view/ERS14961254), study accession number PRJEB51652 (available at http://www.ebi.ac.uk/ena/data/view/PRJEB51652)
- EMO BON water column sample: run accssion number ERS14961281 (available at http://www.ebi.ac.uk/ena/data/view/ERS14961281), study accession number PRJEB51664 (available at http://www.ebi.ac.uk/ena/data/view/PRJEB51664)
- Tara Oceans sample: run accession number ERR599171 (available at https://www.ebi.ac.uk/ena/browser/view/ERR599171, study accession number PRJEB402 (available at https://www.ebi.ac.uk/ena/browser/view/PRJEB402)

Declarations

List of abbreviations

- CDS: Coding Sequences
- CWL: Common Workflow Language
- EMBRC: European Marine Biological Resource Centre
- EMO BON: European Marine Omics Biodiversity Observation Network
- ENA: European Nucleotide Archive
- GO terms: Gene Ontology terms
- GOs: Genomic Observatories
- HPC: High Performance Computing
- LSU: Large Sub Unit
- OSD: Ocean Sampling Day
- RO-Crate: Research Object Crate
- SSU: Small Sub Unit

Ethical Approval

Not applicable.

Consent for publication

Not applicable.

Competing Interests

M.B., L.R. and R.D.F. are members of the MGnify group that is part of the ELIXIR infrastructure. The authors declare that they have no other competing interests.

Funding

This project has received funding from the European Union's Horizon 2020 research and innovation programme under grant agreement No 824087, under the 1st EOSC-Life Digital Life Sciences Open Call (Project ID 14325) and by the European Marine Biological Resource Centre - European Research Infrastructure Consortium (EMBRC-ERIC), which is part of the European Strategy Forum on Research Infrastructures (ESFRI).

Author's Contributions

Conceptualization: C.J.C., R.D.F., C.P.; Project Administration: C.P., A.P., H.Z.; Investigation: H.Z., M.B., S.N., G.D.M., J.M.; Formal Analysis: H.Z.; Software: H.Z., M.B., S.N., J.M., C.J.C.; Methodology: H.Z., S.N., K.E., E.C.; Validation: H.Z., C.P., I.S.; Data Curation: I.S., K.E., C.P., H.Z.; Resources: I.S., R.D.F., L.R., C.J.C., E.P.; Funding Acquisition: C.P., G.K., C.J.C., H.Z., R.D.F.; Writing - Original Draft Preparation: H.Z., C.P.; Writing - Review & Editing: all; Visualization: H.Z.

Acknowledgements

This research was supported in part through computational resources provided by IMBBC (Institute of Marine Biology, Biotechnology and Aquaculture) of the HCMR (Hellenic Centre for Marine Research). Funding for establishing the IMBBC HPC has been received by the MARBIGEN (EU Regpot) project, LifeWatchGreece RI and the CMBR (Centre for the study and sustainable exploitation of Marine Biological Resources) RI. This study received Portuguese national funds from FCT - Foundation for Science and Technology through project UIDB/04326/2020, UIDP/04326/2020 and LA/P/0101/2020, and from the operational programmes CRESC Algarve 2020 and COMPETE 2020 through projects EMBRC.PT ALG-01-0145-FEDER-022121 and BIODATA.PT ALG-01-0145-FEDER-022231 to C.J.C and G.D.M. This work received Computational Time to HPC infrastructures and scientific and technical support from the high-level support team at NCC-Greece. The financial support from the EuroHPC-JU Project 101101903—EuroCC 2 project of the European Commission is acknowledged. Parts of the runs were performed on the MeluXina machine within the project with ID: EHPC-DEV-2022D10-062. The acquisition and operation of the EuroHPC supercomputer is funded jointly by the EuroHPC Joint Undertaking, through the European Union's Connecting Europe Facility and the Horizon 2020 research and innovation programme, as well as the Grand Duché du Luxembourg.

References

1. Louca S, Parfrey LW, Doebeli M. Decoupling function and taxonomy in the global ocean microbiome. Science 2016;353(6305):1272–1277.
2. Doney SC, Ruckelshaus M, Emmett Duffy J, Barry JP, Chan F, English CA, et al. Climate change impacts on marine ecosystems. Annual review of marine science 2012;4:11–37.
3. Chen J, McIlroy SE, Archana A, Baker DM, Panagiotou G. A pollution gradient contributes to the taxonomic, functional, and resistome diversity of microbial communities in marine sediments. Microbiome 2019;7(1):1–12.
4. Caruso G, La Ferla R, Azzaro M, Zoppini A, Marino G, Petochi T, et al. Microbial assemblages for environmental quality assessment: knowledge, gaps and usefulness in the European Marine Strategy Framework Directive. Critical reviews in microbiology 2016;42(6):883–904.
5. Caruso G, Azzaro M, Caroppo C, Decembrini F, Monticelli LS, Leonardi M, et al. Microbial community and its potential as descriptor of environmental status. ICES Journal of Marine Science 2016;73(9):2174–2177.
6. Liu X, Ashforth E, Ren B, Song F, Dai H, Liu M, et al. Bioprospecting microbial natural product libraries from the marine environment for drug discovery. The Journal of Antibiotics 2010;63(8):415–422.
7. Glasl B, Webster NS, Bourne DG. Microbial indicators as a diagnostic tool for assessing water quality and climate stress in coral reef ecosystems. Marine Biology 2017;164(4):1–18.
8. Kopf A, Bicak M, Kottmann R, Schnetzer J, Kostadinov I, Lehmann K, et al. The ocean sampling day consortium. Gigascience 2015;4(1):1–5.
9. Duarte CM. Seafaring in the 21st century: the Malaspina 2010 circumnavigation expedition. Limnology and Oceanography Bulletin 2015;.
10. Sunagawa S, Acinas SG, Bork P, Bowler C, Eveillard D, Gorsky G, et al. Tara Oceans: towards global ocean ecosystems biology.

Nature Reviews Microbiology 2020;18(8):428–445.
11. Zayed AA, Wainaina JM, Dominguez-Huerta G, Pelletier E, Guo J, Mohssen M, et al. Cryptic and abundant marine viruses at the evolutionary origins of Earth's RNA virome. Science 2022;376(6589):156–162.
12. Sunagawa S, Coelho LP, Chaffron S, Kultima JR, Labadie K, Salazar G, et al. Structure and function of the global ocean microbiome. Science 2015;348(6237):1261359.
13. Yelton AP, Acinas SG, Sunagawa S, Bork P, Pedrós-Alió C, Chisholm SW. Global genetic capacity for mixotrophy in marine picocyanobacteria. The ISME journal 2016;10(12):2946–2957.
14. Santi I, Beluche O, Beraud M, Buttigieg P, Casotti R, Cox C, et al. European marine omics biodiversity observation network: a strategic outline for the implementation of omics approaches in ocean observation. Frontiers in Marine Science 2023;10:1118120.
15. Buck M, Hamilton C. The Nagoya Protocol on access to genetic resources and the fair and equitable sharing of benefits arising from their utilization to the Convention on Biological Diversity. Review of European Community & International Environmental Law 2011;20(1):47–61.
16. Kottmann R, Gray T, Murphy S, Kagan L, Kravitz S, Lombardot T, et al. A standard MIGS/MIMS compliant XML Schema: toward the development of the Genomic Contextual Data Markup Language (GCDML). Omics a journal of integrative biology 2008;12(2):115–121.
17. Samuel RM, Meyer R, Buttigieg PL, Davies N, Jeffery NW, Meyer C, et al. Toward a Global Public Repository of Community Protocols to Encourage Best Practices in Biomolecular Ocean Observing and Research. Frontiers in Marine Science 2021;p. 1488.
18. Tamames J, Cobo-Simón M, Puente-Sánchez F. Assessing the performance of different approaches for functional and taxonomic annotation of metagenomes. BMC genomics 2019;20(1):1–16.
19. Uritskiy GV, DiRuggiero J, Taylor J. MetaWRAP—a flexible pipeline for genome-resolved metagenomic data analysis. Microbiome 2018;6(1):1–13.
20. Beghini F, McIver LJ, Blanco-Míguez A, Dubois L, Asnicar F, Maharjan S, et al. Integrating taxonomic, functional, and strain-level profiling of diverse microbial communities with bioBakery 3. Elife 2021;10:e65088.
21. Ewels PA, Peltzer A, Fillinger S, Patel H, Alneberg J, Wilm A, et al. The nf-core framework for community-curated bioinformatics pipelines. Nature biotechnology 2020;38(3):276–278.
22. Straub D, Blackwell N, Langarica-Fuentes A, Peltzer A, Nahnsen S, Kleindienst S. Interpretations of environmental microbial community studies are biased by the selected 16S rRNA (gene) amplicon sequencing pipeline. Frontiers in Microbiology 2020;11:550420.
23. Merkel D. Docker: lightweight linux containers for consistent development and deployment. Linux journal 2014;2014(239):2.
24. Kurtzer GM, Sochat V, Bauer MW. Singularity: Scientific containers for mobility of compute. PloS one 2017;12(5):e0177459.
25. Di Tommaso P, Chatzou M, Floden EW, Barja PP, Palumbo E, Notredame C. Nextflow enables reproducible computational workflows. Nature biotechnology 2017;35(4):316–319.
26. Mölder F, Jablonski K, Letcher B, Hall M, Tomkins-Tinch C, Sochat V, et al. Sustainable data analysis with Snakemake [version 1; peer review: 1 approved, 1 approved with reservations]. F1000Research 2021;10(33).
27. Zafeiropoulos H, Gioti A, Ninidakis S, Potirakis A, Paragkamian S, Angelova N, et al. 0s and 1s in marine molecular research: a regional HPC perspective. GigaScience 2021;10(8):giab053.
28. ATLAS C, Yamamoto S, Shapiro M, Virzi J, Werner M, Venturi M, et al. The simulation principle and performance of the ATLAS fast calorimeter simulation FastCaloSim. ATL-COM-PHYS-2010-838; 2010.
29. Keegan KP, Glass EM, Meyer F. MG-RAST, a metagenomics service for analysis of microbial community structure and function. In: Microbial environmental genomics (MEG) Springer; 2016.p. 207–233.
30. Mitchell AL, Almeida A, Beracochea M, Boland M, Burgin J, Cochrane G, et al. MGnify: the microbiome analysis resource in 2020. Nucleic acids research 2020;48(D1):D570–D578.
31. Chen IMA, Chu K, Palaniappan K, Pillay M, Ratner A, Huang J, et al. IMG/M v. 5.0: an integrated data management and comparative analysis system for microbial genomes and microbiomes. Nucleic acids research 2019;47(D1):D666–D677.
32. Meyer F, Fritz A, Deng ZL, Koslicki D, Lesker TR, Gurevich A, et al. Critical assessment of metagenome interpretation: the second round of challenges. Nature methods 2022;19(4):429–440.
33. Soiland-Reyes S, Sefton P, Crosas M, Castro LJ, Coppens F, Fernández JM, et al. Packaging research artefacts with RO-Crate. Data Science 2021;(Preprint):1–42.
34. Wilkinson MD, Dumontier M, Aalbersberg IJ, Appleton G, Axton M, Baak A, et al. The FAIR Guiding Principles for scientific data management and stewardship. Scientific data 2016;3(1):1–9.
35. Wei Q, Khan IK, Ding Z, Yerneni S, Kihara D. NaviGO: interactive tool for visualization and functional similarity and coherence analysis with gene ontology. Bmc Bioinformatics 2017;18(1):1–13.
36. Amstutz P, Crusoe MR, Tijanić N, Chapman B, Chilton J, Heuer M, et al., Common workflow language, v1. 0. figshare; 2016.
37. Burgin J, Ahamed A, Cummins C, Devraj R, Gueye K, Gupta D, et al. The European Nucleotide Archive in 2022. Nucleic Acids Research 2023;51(D1):D121–D125.
38. Microbiome Informatics ENA fetch tool. MGnify; 2022. https://github.com/EBI-Metagenomics/fetch_tool, original-date: 2018-09-06T15:38:50Z.
39. Chen S, Zhou Y, Chen Y, Gu J. fastp: an ultra-fast all-in-one FASTQ preprocessor. Bioinformatics 2018;34(17):i884–i890.
40. Nawrocki EP, Eddy SR. Infernal 1.1: 100-fold faster RNA homology searches. Bioinformatics 2013;29(22):2933–2935.
41. Matias Rodrigues JF, Schmidt TS, Tackmann J, von Mering C. MAPseq: highly efficient k-mer search with confidence estimates, for rRNA sequence analysis. Bioinformatics 2017;33(23):3808–3810.
42. Milanese A, Mende DR, Paoli L, Salazar G, Ruscheweyh HJ, Cuenca M, et al. Microbial abundance, activity and population genomic profiling with mOTUs2. Nature communications 2019;10(1):1–11.
43. Ondov BD, Bergman NH, Phillippy AM. Interactive metagenomic visualization in a Web browser. BMC bioinformatics 2011;12(1):1–10.
44. Vollmers J, Wiegand S, Kaster AK. Comparing and evaluating metagenome assembly tools from a microbiologist's perspective-not only size matters! PloS one 2017;12(1):e0169662.
45. Li D, Liu CM, Luo R, Sadakane K, Lam TW. MEGAHIT: an ultra-fast single-node solution for large and complex metagenomics assembly via succinct de Bruijn graph. Bioinformatics 2015;31(10):1674–1676.
46. Nurk S, Meleshko D, Korobeynikov A, Pevzner PA. metaSPAdes: a new versatile metagenomic assembler. Genome Research 2017;27(5):824–834. http://genome.cshlp.org/content/27/5/824.abstract.
47. Rho M, Tang H, Ye Y. FragGeneScan: predicting genes in short and error-prone reads. Nucleic acids research 2010;38(20):e191–e191.
48. Jones P, Binns D, Chang HY, Fraser M, Li W, McAnulla C, et al. InterProScan 5: genome-scale protein function classification. Bioinformatics 2014;30(9):1236–1240.
49. Mitchell AL, Attwood TK, Babbitt PC, Blum M, Bork P, Bridge A, et al. InterPro in 2019: improving coverage, classification and access to protein sequence annotations. Nucleic acids research

2019;47(D1):D351–D360.
50. El-Gebali S, Mistry J, Bateman A, Eddy SR, Luciani A, Potter SC, et al. The Pfam protein families database in 2019. Nucleic acids research 2019;47(D1):D427–D432.
51. Haft DH, Selengut JD, Richter RA, Harkins D, Basu MK, Beck E. TIGRFAMs and genome properties in 2013. Nucleic acids research 2012;41(D1):D387–D395.
52. Sigrist CJ, De Castro E, Cerutti L, Cuche BA, Hulo N, Bridge A, et al. New and continuing developments at PROSITE. Nucleic acids research 2012;41(D1):D344–D347.
53. Ashburner M, Ball CA, Blake JA, Botstein D, Butler H, Cherry JM, et al. Gene ontology: tool for the unification of biology. Nature genetics 2000;25(1):25–29.
54. Huerta-Cepas J, Szklarczyk D, Heller D, Hernández-Plaza A, Forslund SK, Cook H, et al. eggNOG 5.0: a hierarchical, functionally and phylogenetically annotated orthology resource based on 5090 organisms and 2502 viruses. Nucleic acids research 2019;47(D1):D309–D314.
55. Cantalapiedra CP, Hernández-Plaza A, Letunic I, Bork P, Huerta-Cepas J. eggNOG-mapper v2: functional annotation, orthology assignments, and domain prediction at the metagenomic scale. Molecular biology and evolution 2021;38(12):5825–5829.
56. Eddy SR. Accelerated profile HMM searches. PLoS computational biology 2011;7(10):e1002195.
57. Aramaki T, Blanc-Mathieu R, Endo H, Ohkubo K, Kanehisa M, Goto S, et al. KofamKOALA: KEGG Ortholog assignment based on profile HMM and adaptive score threshold. Bioinformatics 2020;36(7):2251–2252.
58. Kanehisa M, Sato Y, Kawashima M, Furumichi M, Tanabe M. KEGG as a reference resource for gene and protein annotation. Nucleic acids research 2016;44(D1):D457–D462.
59. Soiland-Reyes S, Sefton P, Crosas M, Castro LJ, Coppens F, Fernández JM, et al. Packaging research artefacts with RO-Crate. Data Science 2022;5(2):97–138.
60. De Geest P, Droesbeke B, Eguinoa I, Gaignard A, Huber S, Leo S, et al., ro-crate-py. Zenodo; 2022. https://doi.org/10.5281/zenodo.6594974, cite as.
61. Santi I, Casotti R, Comtet T, Cunliffe M, Koulouri PY, Macheriotou L, et al. European Marine Omics Biodiversity Observation Network (EMO BON) Handbook (Version 1.0). EMBRC-ERIC; 2021.
62. Pesant S, Not F, Picheral M, Kandels-Lewis S, Le Bescot N, Gorsky G, et al. Open science resources for the discovery and analysis of Tara Oceans data. Scientific data 2015;2(1):1–16.
63. Quince C, Walker AW, Simpson JT, Loman NJ, Segata N. Shotgun metagenomics, from sampling to analysis. Nature biotechnology 2017;35(9):833–844.
64. Faust K, Sathirapongsasuti JF, Izard J, Segata N, Gevers D, Raes J, et al. Microbial co-occurrence relationships in the human microbiome. PLoS computational biology 2012;8(7):e1002606.
65. Friedman J, Alm EJ. Inferring correlation networks from genomic survey data. PLoS computational biology 2012;8(9):e1002687.
66. Bharti R, Grimm DG. Current challenges and best-practice protocols for microbiome analysis. Briefings in bioinformatics 2021;22(1):178–193.
67. Reimer LC, Sardà Carbasse J, Koblitz J, Ebeling C, Podstawka A, Overmann J. Bac Dive in 2022: the knowledge base for standardized bacterial and archaeal data. Nucleic Acids Research 2022;50(D1):D741–D746.
68. Zafeiropoulos H, Paragkamian S, Ninidakis S, Pavlopoulos GA, Jensen LJ, Pafilis E. PREGO: a literature and data-mining resource to associate microorganisms, biological processes, and environment types. Microorganisms 2022;10(2):293.
69. contributors TUoMUaB, Pitfalls and limitations · BioExcel Best Practice Guide: Creating workflows with Common Workflow Language; 2021. http://docs.bioexcel.eu/cwl-best-practice-guide/limitations.html.
70. Vivian J, Rao AA, Nothaft FA, Ketchum C, Armstrong J, Novak A, et al. Toil enables reproducible, open source, big biomedical data analyses. Nature biotechnology 2017;35(4):314–316.
71. Sharpton TJ. An introduction to the analysis of shotgun metagenomic data. Frontiers in plant science 2014;5:209.
72. Liu YX, Qin Y, Chen T, Lu M, Qian X, Guo X, et al. A practical guide to amplicon and metagenomic analysis of microbiome data. Protein & cell 2021;12(5):315–330.
73. Krakau S, Straub D, Gourlé H, Gabernet G, Nahnsen S. nf-core/mag: a best-practice pipeline for metagenome hybrid assembly and binning. NAR Genomics and Bioinformatics 2022;4(1):lqac007.

Point-by-point response to the reviewers
Click here to access/download;Manuscript;metaGOflow point-by-point_ revision2.docx

GIGA-D-23-00127R1
metaGOflow: a workflow for the analysis of marine Genomic Observatories shotgun metagenomics data
Haris Zafeiropoulos; Martin Beracochea; Stelios Ninidakis; Katrina Exter; Antonis Potirakis; Gianluca De Moro; Lorna Richardson; Erwan Corre; João Machado; Evangelos Pafilis; Ioulia Santi; Georgios Kotoulas; Robert Daniel Finn; Cymon Cox; Christina Pavloudi
GigaScience

Dear Dr Zafeiropoulos,

Your manuscript "metaGOflow: a workflow for the analysis of marine Genomic Observatories shotgun metagenomics data" (GIGA-D-23-00127R1) has been assessed by our reviewers. Based on these reports, and my own assessment as Editor, I am pleased to inform you that it is potentially acceptable for publication in GigaScience, once you have carried out some essential revisions suggested by our reviewers.

Reviewer #1 strongly recommends a proper description and manual for ease of use by the broader community, as they feel that without it, this will not be easy to reuse. So please provide this.

Their reports, together with any other comments, are below. Please also take a moment to check our website at https://www.editorialmanager.com/giga/ for any additional comments that were saved as attachments.

In addition, please register any new software application in the bio.tools and SciCrunch.org databases to receive RRID (Research Resource Identification Initiative ID) and biotoolsID identifiers, and include these in your manuscript. Computational workflows should be registered in workflowhub.eu and the DOIs cited in the relevant places in the manuscript. These will facilitate tracking, reproducibility and re-use of your tool.

Please include a point-by-point within the 'Response to Reviewers' box in the submission system. Please ensure you describe additional experiments that were carried out and include a detailed rebuttal of any criticisms or requested revisions that you disagreed with. Please also ensure that your revised manuscript conforms to the journal style, which can be found in the Instructions for Authors on the journal homepage. If the data and code has been modified in the revision process please be sure to update the public versions of this too.

The due date for submitting the revised version of your article is 15 Oct 2023.

We look forward to receiving your revised manuscript soon.

Best wishes,

Nicole Nogoy, Ph.D
GigaScience
www.gigasciencejournal.com

Dear Editor,

We would like to kindly thank both you and the reviewer for the time you spent to thoroughly review our manuscript for a second time.
In this revised version of our manuscript, we have addressed the reviewer’s comments and suggestions and, where necessary, we have incorporated changes and made amendments and alterations to the manuscript.
Our revision focuses mostly on your suggestion for a thorough description and manual for ease of use by the broader community.
This is now available through https://metagoflow.readthedocs.io/.

Reviewer 1

While, in this reviewer's opinion, the pipeline is a very useful contribution to the field, the authors are shooting themselves in the foot by not providing sufficient explanations in either manuscript or manual/wiki to make it usable by the community. The revised manuscript does show improvement in several areas, however most of my concerns were not addressed. As such, I cannot endorse the publication at this stage but will be happy to re-review one more time if the authors decide to address the comments and re-submit.
We would like to thank the reviewer for his comment on the value of our workflow.
The phrase “shooting ourselves in the foot” seems a little inappropriate, as we addressed all of the reviewer’s concerns in the revised version of the manuscript.

In general, the authors need to decide whether MetaGOflow is an internal EMO BON routine or is widely applicable. If it is an internal routine, then it does not need to be published in GigaScience and can be reported in the EMO BON handbook. If the authors still think that MetaGOflow is widely applicable and of interest to the wider audience of GigaScience, they need to provide a much better scientific description of their pipeline, inputs and scientific outputs/applications than what is currently presented in either the paper or the manual/wiki. Not having this information will make it extremely difficult for other researchers to use the pipeline and to compare it with other available tools. Current paper and wiki/manual seem to be written for people who routinely use the pipeline within the EMO BON project, not for the wider audience.
We do not need to decide on whether metaGOflow is an internal EMO BON routine or not. We have already shown that it is indeed widely applicable. We have analysed shotgun metagenomics samples from different sequencing platforms and different sample types (water, sediment, human gut, fish gut), so we don’t understand why the reviewer still thinks that metaGOflow is not widely applicable. The metagenomics community is now an established field with several reviews, tutorials, online material so it is our strong belief that we do not need to define and repeat its basic concepts. On top of that, when we provide the description of a metaGOflow data product (e.g. “Krona summary of LSU taxonomic inventory”) we consider it is common knowledge among the community and definitely not an EMO BON internal term. The same applies for all the description files we had included in our first revision under the following link: https://github.com/emo-bon/MetaGOflow/wiki/metaGOflow-overview.
metaGOflow couldn’t have been characterised solely as an internal EMO BON routine. It is built on the MGnify pipeline which is a pipeline used ambiguously and for several different sample types. In our revised version of the manuscript, we have also included a ReadTheDocs manual for metaGOflow at https://metagoflow.readthedocs.io.
As we realise there is an inadequate understanding of the extent of the description of the input/output files necessary, in the ReadTheDocs page, we have added extended descriptions so even a non-familiar to the metagenomics field user may benefit from metaGOflow. There is also an extended description on how to install and run metaGOflow; each parameter and argument is discussed separately and hints are provided in cases they need attention (e.g. how to pick the chunk sizes). There is also a Frequent Asked Questions (FAQs) section which we intend to update from time to time with questions coming from the users.

Please try to imagine your reader as a researcher who sent the sample for metagenomic sequencing and received the fastq files that need to be analyzed. Such reader would want to go through your manuscript and wiki and quickly understand:
Can I use metaGOflow with my read data?
Do I need to adjust my data and if so, how?
What results will I get?
How can I use these results?

What scientific conclusions will I be able to make?
How are these results different from whatever I can obtain from other pipelines?
From our experience as users, when we want to run a workflow/pipeline/tool, we inspect the tool's github page and not the manuscript. This is why we created the wiki page, which answers the questions "Can I use metaGOflow with my read data?", "Do I need to adjust my data and if so, how?", "What results will I get?". In this revised version, we have also created a manual to assist potential users as suggested by the reviewer. Scientific conclusions are a matter of the researchers analysing their data and not something that we can decide upon and, also, thoroughly describe. The question "How are these results different from whatever I can obtain from other pipelines?" can be answered in a benchmarking study comparing different pipelines, which is not the scope of our manuscript.
The ReadTheDocs manual page we provide in this revised version, addresses these questions to some extent.

The authors argue that in the "manuscript we are not aiming to describe what metagenomics is, or how it's used, or how it's interpreted." The authors may want to reconsider.Many creators of other tools go well out of their way to very plainly explain how their software is used, what it produces, and how to interpret the results using varied examples.
Explaining how the software is used is not the same thing as explaining what metagenomics is.
It is not in the scope of our manuscript to explain a term and a methodology that has been applied extensively for over two decades. There are several great review papers that deal with explaining what shotgun metagenomics is; in our revised version of the manuscript we have added such papers as citations and prompted the reader to access them for more information (see *Specific questions Issue 1.3 and 1.9*).
Also, our target audience is not researchers who have no idea what shotgun metagenomics is. We are presenting a workflow for the analysis of shotgun metagenomics data, so our target audience is researchers who have already obtained data so they are already aware of the methodology.

Specific questions:

Issue 1.3 and 1.9: This is far from sufficient description. The new figure 3 shows the json structure but does not provide any usable information. I understand that the authors are trying to keep the manuscript short, but they can include more information in the manual. The manual needs to include the scientific description of each input and output file and its contents (What are the rows and columns?). How the data are supposed to be interpreted: e.g., are the base quality plots shown in Fig. 1b good or bad?
In the revised version of our manuscript, we have included a manual for metaGOflow. The description of each output file can be found at the *Description of metaGOflow's data products* page. In the revised manuscript we have added links to the manual. On top of that, being a pipeline, metaGOflow is strongly related to the original tools it invokes. Thus, it inherits all their documentation, tutorials etc and metaGOflow users are always able to take them into consideration. For example, the answer to whether the base quality plots in Fig. 1b are good or bad is available on this video, which we now point to at the manual page.
Last, we have added a new section in the main manuscript called "Parameters tuning" discussing the parameters and the arguments the user needs to set to run metaGOflow; a specific section is also available in the ReadTheDocs manual for this (*Arguments and parameters*).

Issue 1.4: Thank you for addressing some of these issues, we understand it can be challenging to create packages that work "out of the box" so to speak that do not require at least some kind of environment setup. However, if the intention of metaGOflow is to be used by "professional systems administrators" of larger computing infrastructure, this should be explicitly stated or at least remove mentions of the software being "easy-to-use".
"Easy-to-use" and "professional systems administrators" are not terms that exclude one another.

Issue 1.5: The use of metaGOflow on fish and human gut samples illustrates a wider applicability of the pipeline. I appreciate the inclusion of these results in the manuscript. Regarding the host sequences: would it be possible to include an optional step for their removal? This will make the pipeline easier to use for researchers.

Unfortunately, it is not possible to include an optional step for host removal. This can be done by the researchers before (or after) they analyse their data, as it is study-specific.

Issue 1.7: I appreciate inclusion of the table listing the pipeline steps. In addition, a similar table comparing the scientific results produced by each pipeline needs to be included.
This would be the result of a benchmarking study, which is not the scope of our manuscript.

Issue 1.8: The readers should not be forced to browse the Handbook to figure out what kind of data can be analyzed with the pipeline. Specifically, the question before the user is whether the pipeline is dependent on what adapters are used for library construction.
We have shown that metaGOflow can analyse data from a variety of sequencing platforms, hence we have shown its robustness. We are not prompting the user to browse through the EMO BON Handbook to figure out what kind of data can be analysed with the pipeline.

Issue 1.10. The authors mention that some of the data can be visualized with tools like "ShinyGO, DICE, KOBAS-i and more. Please provide brief instructions for doing this together with the scientific description of results as discussed in 1.3.
This was mentioned in our first point-by-point response to highlight the great number of tools and available options for a researcher to create visualisations based on the annotation type and the questions of interest. Describing how to use each of those tools (that are not part of metaGOflow) is out of scope of this manuscript.

Issue 1.11 The explanation of the further use of data included in the latest version is insufficient. How are the outputs of the pipeline used at EMO BON? The authors need to significantly expand on this topic in the manual/wiki if not in the manuscript itself. Specifically, this discussion should include what tools can be used (e.g. LefSE), what files can be used as input (i.e. extracted from the RO-crate), do they need to be modified, example scripts.
The metaGOflow manuscript is not aiming to answer the "How are the outputs of the pipeline used at EMO BON?". This is something that is decided by the EMO BON community; if we had to guess, it would not be in a single thing as different scientific questions will require different downstream analysis starting from the metaGOflow data products. What subsequent tools are going to be used and which scientific questions are going to be addressed is not related to the scope of this manuscript. We cannot explain how researchers can proceed with their analyses after they retrieve the metaGOflow outputs; there are plenty of scientific questions that can be addressed with these outputs and, thus, plenty of subsequent types of analyses. If we suggest one type of analysis (e.g. LefSe), then we would need to suggest all the potential types which, again, is not something that is in the scope of our manuscript.
Throughout our manuscript, we have made clear that metaGOflow is a pipeline that gets shotgun metagenomics sequences as raw data and returns taxonomic and functional profiles at the sample level. We do not argue about supporting any further analysis. Thus, it is beyond the scope of this manuscript to discuss these topics. Again, a great number of studies have been published discussing how to address the various scientific questions one might address using shotgun metagenomics; in this revised version of our manuscript we have added only a few of them indicatively (see Discussion and Conclusions section) .

Figure 1

Click here to access/download;Figure;eosc-life-marine-gos-pipeline.drawio.png

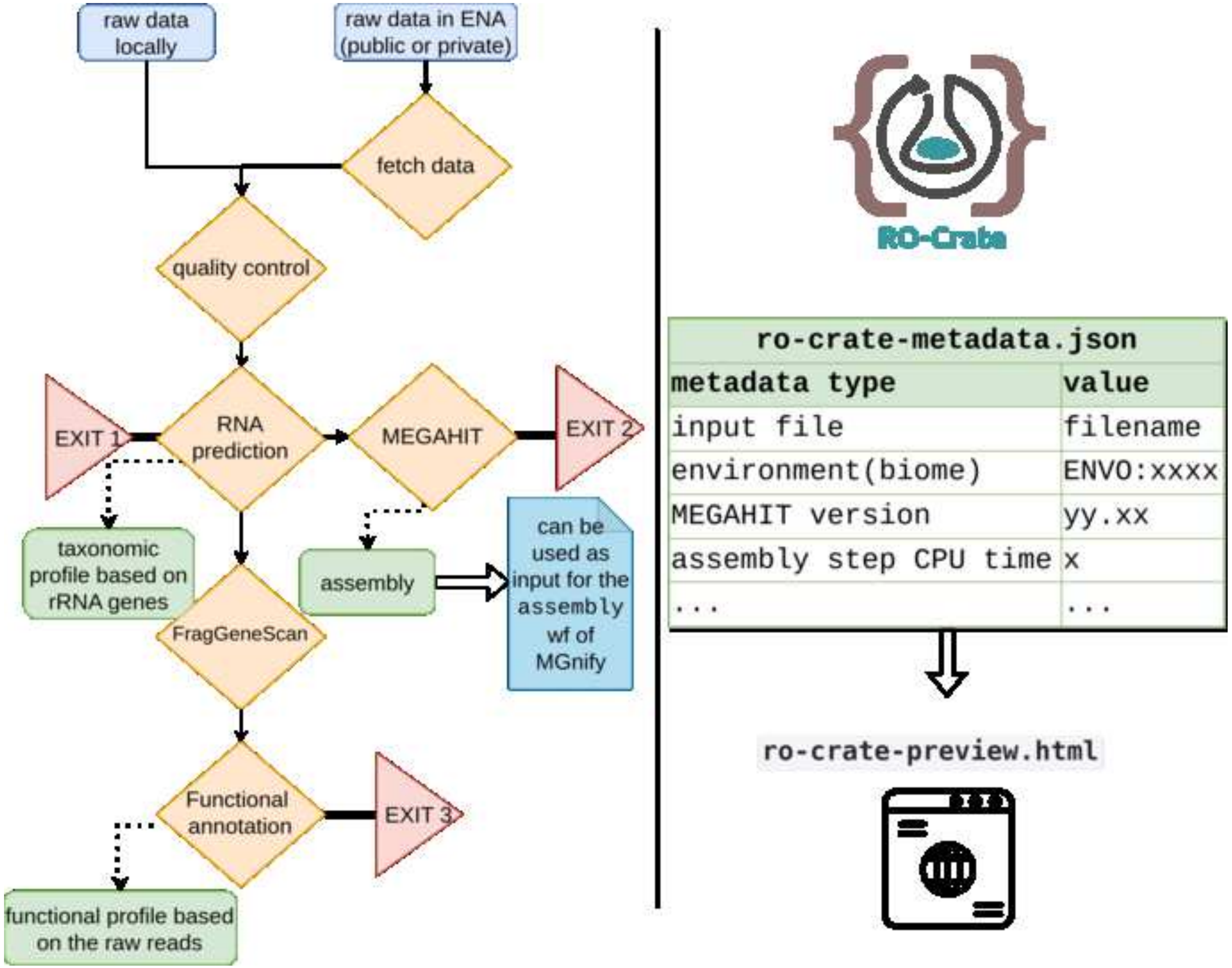

Figure 2

Click here to access/download;Figure;metagoflow-results.drawio.png

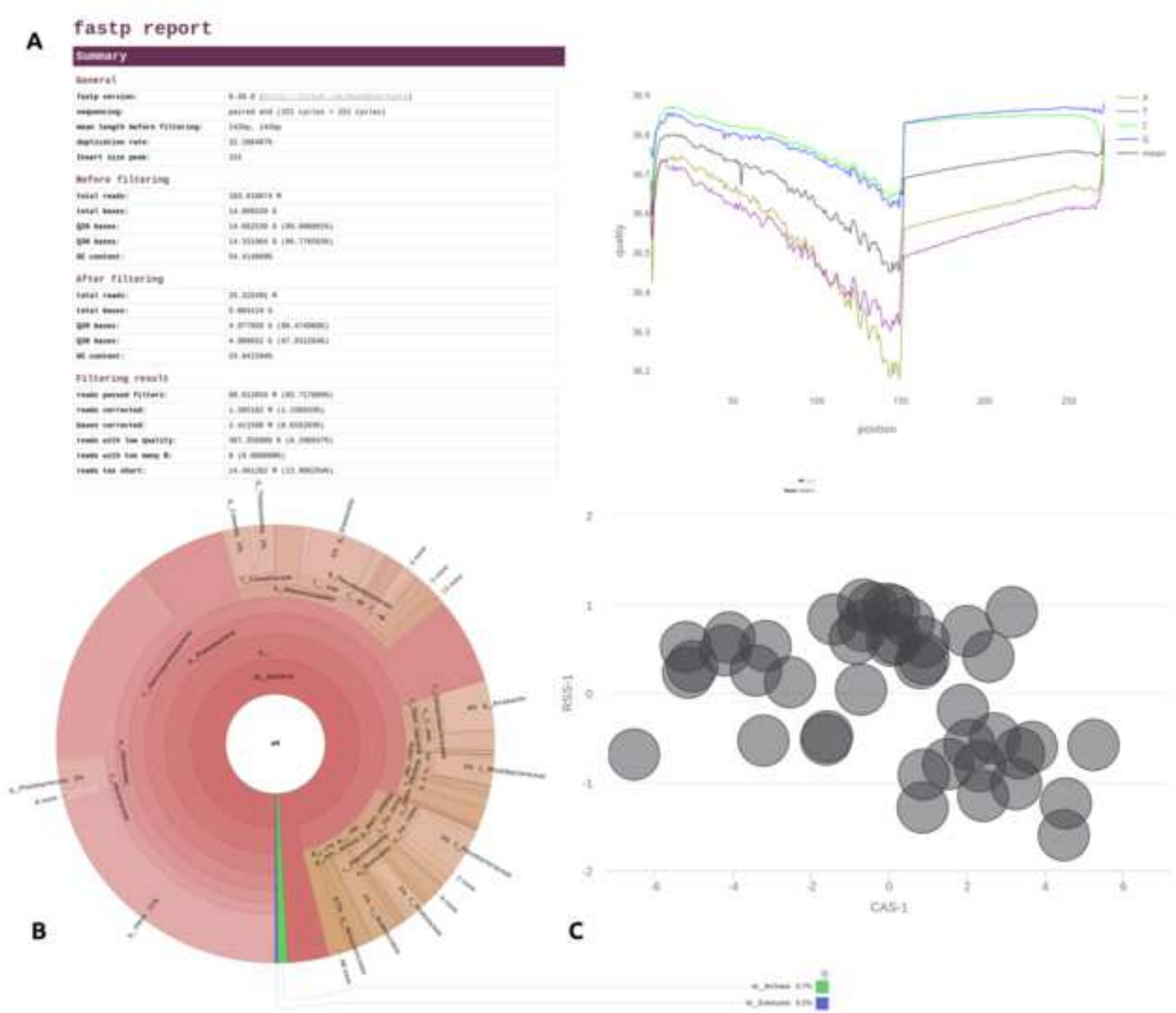

```
{
    "@id": "results/functional-annotation/stats/interproscan.stats",
    "@type": "File",
    "encodingFormat": "text/plain",
    "name": "InterProScan summary statistics"
},
{
    "@id": "results/functional-annotation/stats/go.stats",
    "@type": "File",
    "encodingFormat": "text/plain",
    "name": "Geno Ontology summary statistics"
},
{
    "@id": "results/functional-annotation/stats/ko.stats",
    "@type": "File",
    "encodingFormat": "text/plain",
    "name": "Kegg Ontology summary statistics"
},
{
    "@id": "results/functional-annotation/stats/pfam.stats",
    "@type": "File",
    "encodingFormat": "text/plain",
    "name": "Pfam summary statistcs"
},
{
    "@id": "results/functional-annotation/stats/orf.stats",
    "@type": "File",
    "encodingFormat": "text/plain",
    "name": "ORF summary statistics"
},
{
    "@id": "https://www.apache.org/licenses/LICENSE-2.0",
    "@type": "CreativeWork",
    "identifier": "https://spdx.org/licenses/Apache-2.0.html",
    "name": "Apache License 2.0"
},
```

Supplementary Table 1

Click here to access/download
Supplementary Material
metaGOflow_Supplementary-Table-1.docx
