# Supplementary material for: metaGOflow: a workflow for the analysis of marine Genomic Observatories shotgun metagenomics data
Source: Gigascience. 2023 Oct 18;12:giad078. doi: 10.1093/gigascience/giad078 (PMC10583283; doi:10.1093/gigascience/giad078)
Supplement: giad078_GIGA-D-23-00127_Revision_4 [file giad078_giga-d-23-00127_revision_4.pdf]

GigaScience

metaGOflow: a workflow for the analysis of marine Genomic Observatories shotgun metagenomics data

--Manuscript Draft--

**Manuscript Number:**	GIGA-D-23-00127R4
**Full Title:**	metaGOflow: a workflow for the analysis of marine Genomic Observatories shotgun metagenomics data
**Article Type:**	Technical Note
**Funding Information:**	HORIZON EUROPE European Research Council (824087) — Not applicable<br>European Marine Biological Resource Centre — Not applicable
**Abstract:**	Background: Genomic Observatories (GOs) are sites of long-term scientific study that undertake regular assessments of the genomic biodiversity. The European Marine Omics Biodiversity Observation Network (EMO BON) is a network of GOs that conduct regular biological community samplings to generate environmental and metagenomic data of microbial communities from designated marine stations around Europe. The development of an effective workflow is essential for the analysis of the EMO BON metagenomic data in a timely and reproducible manner.<br>Findings: Based on the established MGnify resource we developed metaGOflow; metaGOflow supports the fast inference of taxonomic profiles from GO-derived data based on rRNA genes and their functional annotation using the raw reads. Thanks to the Research Object Crate (RO-Crate) packaging, relevant metadata about the sample under study, and the details of the bioinformatics analysis it has been subjected to, are inherited to the data product while its modular implementation allows running the workflow partially. The analysis of two EMO BON and one Tara Oceans samples was performed as a use case.<br>Conclusions: metaGOflow is an efficient and robust workflow that scales to the needs of projects producing big metagenomic data such as EMO BON. It highlights how containerization technologies along with modern workflow languages and metadata package approaches can support the needs of researchers when dealing with ever-increasing volumes of biological data. Despite being initially oriented to address the needs of EMO BON, metaGOflow is a flexible and easy-to-use workflow that can be broadly used for one-sample-at-a-time analysis of shotgun metagenomics data.
**Corresponding Author:**	Haris Zafeiropoulos<br>KU Leuven Rega Institute for Medical Research.: Katholieke Universiteit Leuven Rega Institute for Medical Research<br>Leuven, BELGIUM
**Corresponding Author Secondary Information:**	
**Corresponding Author's Institution:**	KU Leuven Rega Institute for Medical Research.: Katholieke Universiteit Leuven Rega Institute for Medical Research
**Corresponding Author's Secondary Institution:**	
**First Author:**	Haris Zafeiropoulos
**First Author Secondary Information:**	
**Order of Authors:**	Haris Zafeiropoulos

Powered by Editorial Manager® and ProduXion Manager® from Aries Systems Corporation

	Martin Beracochea
	Stelios Ninidakis
	Katrina Exter
	Antonis Potirakis
	Gianluca De Moro
	Lorna Richardson
	Erwan Corre
	João Machado
	Evangelos Pafilis
	Ioulia Santi
	Georgios Kotoulas
	Robert Daniel Finn
	Cymon Cox
	Christina Pavloudi
**Order of Authors Secondary Information:**	
**Response to Reviewers:**	URLs have been now fixed.
**Additional Information:**	
**Question**	**Response**
Are you submitting this manuscript to a special series or article collection?	No
**Experimental design and statistics**<br><br>Full details of the experimental design and statistical methods used should be given in the Methods section, as detailed in our Minimum Standards Reporting Checklist. Information essential to interpreting the data presented should be made available in the figure legends.<br><br>Have you included all the information requested in your manuscript?	Yes
**Resources**<br><br>A description of all resources used, including antibodies, cell lines, animals and software tools, with enough information to allow them to be uniquely identified, should be included in the Methods section. Authors are strongly encouraged to cite Research Resource	Yes

Identifiers (RRIDs) for antibodies, model organisms and tools, where possible.<br><br>Have you included the information requested as detailed in our Minimum Standards Reporting Checklist?	
**Availability of data and materials**<br><br>All datasets and code on which the conclusions of the paper rely must be either included in your submission or deposited in publicly available repositories (where available and ethically appropriate), referencing such data using a unique identifier in the references and in the “Availability of Data and Materials” section of your manuscript.<br><br>Have you have met the above requirement as detailed in our Minimum Standards Reporting Checklist?	Yes

LaTeX - Main Document (TeX file) Click here to access/download;LaTeX - Main Document (TeX file);main.tex

OXFORD (GIGA)^n SCIENCE

GigaScience, 2022, 1–10

doi: xx.xxxx/xxxx
Manuscript in Preparation
Technical Note

TECHNICAL NOTE

metaGOflow: a workflow for the analysis of marine Genomic Observatories shotgun metagenomics data

Haris Zafeiropoulos[1,2] *, Martin Beracochea[3] *, Stelios Ninidakis[1], Katrina Exter[4], Antonis Potirakis[1], Gianluca De Moro[5], Lorna Richardson[3], Erwan Corre[6], João Machado[5], Evangelos Pafilis[1], Georgios Kotoulas[1], Ioulia Santi[7,1], Robert D. Finn[3], Cymon J. Cox[5] and Christina Pavloudi[1,8] †

[1] Institute of Marine Biology, Biotechnology and Aquaculture (IMBBC), Hellenic Centre for Marine Research (HCMR), Former U.S. Base of Gournes P.O. Box 2214, 71003, Heraklion, Crete, Greece and [2] KU Leuven, Department of Microbiology, Immunology and Transplantation, Rega Institute for Medical Research, Laboratory of Molecular Bacteriology, 3000 Leuven, Belgium and [3] European Molecular Biology Laboratory, European Bioinformatics Institute (EMBL-EBI), Wellcome Genome Campus, Hinxton, Cambridge CB10 1SD, UK and [4] Flanders Marine Institute (VLIZ), Oostende, Belgium and [5] Centro de Ciências do Mar (CCMAR), Universidade do Algarve, Campus de Gambelas, 8005-139, Faro, Portugal and [6] CNRS, FR 2424, ABiMS Platform, Station Biologique de Roscoff (SBR), Roscoff, France and [7] European Marine Biological Resource Centre (EMBRC-ERIC), Paris, France and [8] Department of Biological Sciences, The George Washington University, District of Columbia, USA

* Corresponding authors: haris.zafeiropoulos@kuleuven.be & mbc@ebi.ac.uk
† Current affiliation: PSL Research University: EPHE-UPVD-CNRS, UAR CNRS 3278 Centre de Recherche Insulaire et Observatoire de l'Environnement (CRIOBE), France & Laboratoire d'Excellence "CORAIL", Centre de Recherche Insulaire et Observatoire de l'Environnement (CRIOBE), French Polynesia

Abstract

Background: Genomic Observatories (GOs) are sites of long-term scientific study that undertake regular assessments of the genomic biodiversity . The European Marine Omics Biodiversity Observation Network (EMO BON) is a network of GOs that conduct regular biological community samplings to generate environmental and metagenomic data of microbial communities from designated marine stations around Europe. The development of an effective workflow is essential for the analysis of the EMO BON metagenomic data in a timely and reproducible manner.
Findings: Based on the established MGnify resource we developed metaGOflow. metaGOflow supports the fast inference of taxonomic profiles from GO-derived data based on rRNA genes and their functional annotation using the raw reads. Thanks to the Research Object Crate (RO-Crate) packaging, relevant metadata about the sample under study, and the details of the bioinformatics analysis it has been subjected to, are inherited to the data product while its modular implementation allows running the workflow partially. The analysis of two EMO BON and one Tara Oceans samples was performed as a use case.
Conclusions: metaGOflow is an efficient and robust workflow that scales to the needs of projects producing big metagenomic data such as EMO BON. It highlights how containerization technologies along with modern workflow languages and metadata package approaches can support the needs of researchers when dealing with ever-increasing volumes of biological data. Despite being initially oriented to address the needs of EMO BON, metaGOflow is a flexible and easy-to-use workflow that can be broadly used for one-sample-at-a-time analysis of shotgun metagenomics data.

Key words: shotgun metagenomics; MGnify; Common Workflow Language (CWL); containers; provenance; RO-Crate

Compiled on: September 8, 2023.
Draft manuscript prepared by the author.

Introduction

It is well established that microbial assemblages support multiple ecosystem services and that microbial community profiling using metagenomics methods can help elucidate the mechanisms that govern the structure of these communities and their interactions with the environment [1]. The community composition and structure of marine microbiome is directly correlated with environmental quality [2, 3]. Indeed, the quality of a marine microbial environment (e.g. a marine sediment) can impact the food chain [4] through the physical and chemical effects of secondary metabolites [5]. In addition, secondary metabolites produced by microorganisms may also become targets for bio-prospecting in medicine and industry [6]. Monitoring the changes in microbial community composition and function due to climate change-related stressors, such as ocean acidification or increases in temperature and UV absorption, can provide insights on ecosystem function, health, and resilience [7].

Pioneering research programmes such as the Ocean Sampling Day (OSD) [8], Malaspina circumnavigation expedition [9], and Tara Oceans [10], have been instrumental in collecting large series' of marine genomic samples from sites around the globe. The analysis of data resulting from these studies has greatly increased our understanding of the importance, the role, and the mechanisms governing microbial communities in some of the most common, sensitive or threatened marine environments [11, 12, 13]. EMO BON [14], a European Marine Biological Resource Centre (EMBRC-ERIC) [15] initiative, is designed to continue and expand this effort by regular bimonthly microbial genomic biodiversity samplings at designated marine coastal stations around the European coastline. In the first two years of the EMO BON (2021-2022) it is expected that more than 540 shotgun metagenomic data sets from water column and sediment samples will be generated from 17 European sites.

The ultimate success of GOs depends on the development and adoption of standards for sampling, metadata collection, sequencing, and data analysis. The provision of metadata relating to the raw sequence data, data products, and their analysis methods, are of high importance for interpretation and interoperability, and need to be accessible in both human- and machine-readable formats. Legislative framework, such as the Nagoya Protocol for Access and Benefit Sharing (ABS) [16], and community written frameworks, such as those developed by the Genomic Standards Consortium (GSC) [17, 18], as well as initiatives encouraging adherence to best practices, such as the Better Biomolecular Ocean Practices (BeBOP) project [19, 20], have all been key to providing agreed-upon standard that aim to fulfil these needs. Standard operating procedures and standardised methods of analysis enable the comparison of results among sites, through time, and among projects, without which, much of the value of the data for environmental assessment is lost.

Effective analysis of shotgun metagenomic data is time-consuming, especially regarding computational steps such as sequence assembly and annotation [21]. Moreover, microbial community profiling and functional analyses are most useful when samples are maximally comparable in space and time, and have been thereby treated using the same analytical procedures. To address the challenges that arise when analyzing metagenomic data, numerous workflows and pipelines have been developed. Notable pipelines include metaWRAP [22], bioBakery [23], and nf-core [24], which provides a collection of pipelines such as nf-core/ampliseq [25] and nf-core/taxprofiler [24]. Recently, containerization approaches (e.g., Docker [26], Singularity [27] etc.), along with workflow managers (e.g., Nextflow [28], Snakemake [29] etc.), have been widely used to a) address the complexity of the analysis, b) facilitate execution and reproducibility and c) distribute and share software to a broader audience [30]. nf-core and ATLAS [31] shotgun metagenomic analysis pipelines are examples of the implementation of such approaches.

Additionally, there are (data analysis) resources like MG-RAST [32], MGnify [33], and IMG/M [34] that come with their own distinct advantages and disadvantages.

The computing requirements for the analysis of the EMO BON data may exceed the computing capacity that a single research institute and/or a regional High Performance Computing (HPC) (i.e., Tier 2) systems can support using the available workflows. Indicatively, for a single dataset, software tools related to the retrieval of taxonomic profiles require up to 160 CPU hours and up to 100 GB of RAM [35]. Computing requirements for the functional annotation of shotgun reads are even higher. Nevertheless, timely provision of data and data products from GOs is of paramount importance to facilitate long-term ecological studies, to accelerate policy-making, and to directly assess the impact of anthropogenic effects on the marine environment.

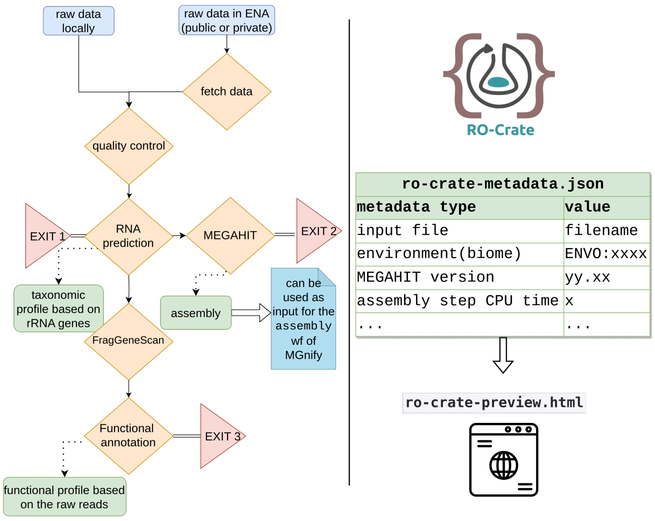

Figure 1. Schematic overview of metaGOflow, showing the main steps of the analysis along with their corresponding data products; the partial execution of the workflow is also shown by the potential exit points (left). Independent of the steps to be performed, once completed, an RO-Crate is built (right).

To address the challenges of analysing GO data in a timely and standardised framework we developed metaGOflow: a MGnify-based [33] computational workflow that implements the critical steps of a shotgun metagenomic bioinformatics analysis, and provides rich provenance metadata describing the data, data products, and workflow execution (Figure 1). The novel aspects of this workflow are mainly a) partial workflow execution; e.g. the user has the flexibility to choose whether to run the functional annotation sub-workflow or not, or even run it at a later point using the data products of the previous steps, b) the incorporation of an alternative assembler with a significantly lower computational cost as compared to the MGnfiy default one and c) the ultimate generation and verification of a Research Object (RO) crate ensuring the workflow's FAIRness. On top of that, several updates of the databases and tools invoked by MGnify have been performed.

metaGOflow consists of two basic concepts:

- an *analytical workflow* which provides taxonomic inventories and community gene function profiles of the samples as data products packaged in RO Crates [36],
- a *data provenance workflow* that generates extensive metadata and thereby provides compliance of the data, data products, and analytical procedures with FAIR data practices and the principles of Open Science, also packaged in the RO Crates [37, 19].

Implementation

Overview

The pillars around which metaGOflow [SCR_023674] has been built, namely containerisation technologies such as Docker [26] and Singularity [27], and the Common Workflow Language (CWL) [39, 40], ensure the workflow's ability to perform in different HPC and cloud computing platforms, following the MGnify example.

metaGOflow inherits the architecture of MGnify pipeline-v5 [41] and exploits several of the already containerized tools and the sub-workflows implemented in the MGnify pipeline. Several enhancements and upgrades allow metaGOflow to make use of the latest versions of the tools and databases invoked. metaGOflow makes extensive use of CWL *subworkflows* and *conditional* step execution to address the specific needs of the EMO BON project from a computing resources point of view.

For example, the user can run the workflow to only generate the taxonomic inventory of a sample. Then, at a later time and by using the output of the first analysis, the user can also generate the assembly of this sample's reads and/or their functional annotation. This flexibility in the workflow is essential as there are a considerable number of samples to be analysed (preferably in as short a period of time as possible), and the computing requirements, especially for the functional annotation step, can be substantial (see Table 2).

In its current version (v.1.0.1[42]), metaGOflow has 5 distinct steps. As in MGnify, metaGOflow analyses a single sample at a time (see Figure 1). The user may either provide locally stored raw data (`.fastq` files) or start the workflow by giving a European Nucleotide Archive (ENA)[43] run accession number. In the later case, metaGOflow invokes the `fetch_tool` [44] to retrieve the raw sequence files from ENA; if the data to be retrieved are held privately, the username and password of the associated ENA account are also requested. The user sets the steps of the workflow to be performed and provides values for certain tool parameters through a text-based configuration file (`config.yml`).

To enhance the FAIRness of the data products and of the bioinformatic analysis, metaGOflow data products are packages as RO-Crates: this allows the set of files to be semantically described, to be accompanied by the metadata that describe the precise steps of the workflow execution, the tools and the parameters used, and to flag the specific input and output files. This description is provided in a `JSON-LD` file following a particular (user-generated) profile. Along with the data products, the RO-crate contains information describing the version of the workflow *per se*, including the software and database versions that it uses.

A comparison of the main features of metaGOflow with other commonly used pipelines for shotgun metagenomic analysis is given in Table 1.

metaGOflow is available on GitHub [45]. A Continuous Integration/Continuous Deployment (CI/CD) workflow using GitHub Actions ensures the validity of the workflow's `cwl` main script and, therefore, of all its components. A thorough description of how to install and use metaGOflow, as well as common errors that might occur during the analysis of a sample can be found at its wiki page [46], as well as on its main documentation page [47]. The databases to be installed before using metaGOflow, require 160GB of storage and as a rule of thumb, the user should allocate 1TB of storage to perform a metaGOflow analysis.

The development and testing of metaGOflow was performed in the IMBBC HCMR "Zorbas" HPC [30] and at the HPC facility of CCMAR. Further testing was performed on the Luxembourg national supercomputer MeluXina [48]. The use case experiments (see Section 6) were performed in a "fat" node of the "Zorbas" HPC (2x Inter(R) Xeon(R) Gold 6230 CPU @ 2.10GHz 40 cores and 500 GB).

Step 1: Sequence preprocessing

Sequences are filtered and merged using `fastp` (version 0.20.0) [49]. Short, low quality, and non-merging sequences are removed and a series of statistical tests describing the quality of the sequencing are performed. An `.html` file returned by the `fastp` tool, provides visualizations of these statistics (see Figure 2A). The filtered sequences and the merged filtered sequences are returned as `.fasta` files.

Step 2: Taxonomy inventory

metaGOflow makes use of the `esl-sfetch` miniapp of the EASEL library (S.R. Eddy, unpublished) to index the filtered sequences and support fast sequence retrieval. Then `cmsearch`, an Infernal program [50, 51], is performed using the ribosomal and the non-coding RNA (ncRNA) Rfam covariance models (CM) (version v13.0) against the filtered sequences. Eventually, this is followed by taxonomic classification using `MAPseq` (v 1.2.3) [52] and the SILVA database (version 132) for the taxonomic classification of the SSU and the LSU sequences, while `mOTUs2` [53] quantifies both known and unknown taxa on the filtered sequences. metaGOflow automatically returns Krona plots (an interactive visualization approach of hierarchical data as multi-layered pie charts [54]) using the taxonomic assignments made for the SSU and LSU genes (see Figure 2B).

Step 3: Assembly

Shotgun metagenomic read assembly requires significant computing resources as discussed in Mitchell et. *al* [33] and in Vollmers et. *al* [55]. The extent of the computational "burden" depends heavily on the chosen algorithm. To be able to handle the vast amount of data produced by EMO BON in a timely manner, and since we aim more at unravelling biodiversity at the community, rather than at the individual (i.e. species), level, metaGOflow makes use of the MEGAHIT algorithm [56]. Longer contigs would be returned if e.g. metaSPAdes [57] was employed, but given metaGOflow's high pace data generation and analysis needs, the MEGAHIT algorithm seems a better match.

Step 4: Gene prediction on the reads

metaGOflow performs gene prediction using FragGeneScan (v1.20) [58] like MGnify. This step is a prerequisite for the functional annotation of the reads (Step). To partially run this step, the user needs to provide the merged filtered `.fasta` file, provided by the sequence preprocessing step.

Step 5: Functional annotation of the reads

metaGOflow focuses on the potential metabolic processes of the whole community rather than the processes of each individual species. Therefore, it performs functional annotation at the reads level. Using InterProScan (v.57-90) [59] metaGOflow annotates the reads with InterPro5 [60], PFam [61], TIGRFAM [62], ProSite patterns and profiles [63] and Gene Ontology (GO) [64] terms. Functional annotations are returned as text files. Both GO and GO Slim (available at geneontology.org) annotations are returned. EggNOG5 [65] annotation is also performed using to the eggnog-mapper (v2.1.8) [66]. Last, metaGOflow invokes the HMMER [67] tool along with the KOfam library [68] to get KEGG orthology annotations [69]. This step requires a significant amount of computing time.

To partially run this step, the user needs to provide the merged filtered `.fasta` file, provided by the sequence preprocessing step (Step 1) as well as the output of the gene prediction step (Step 4).

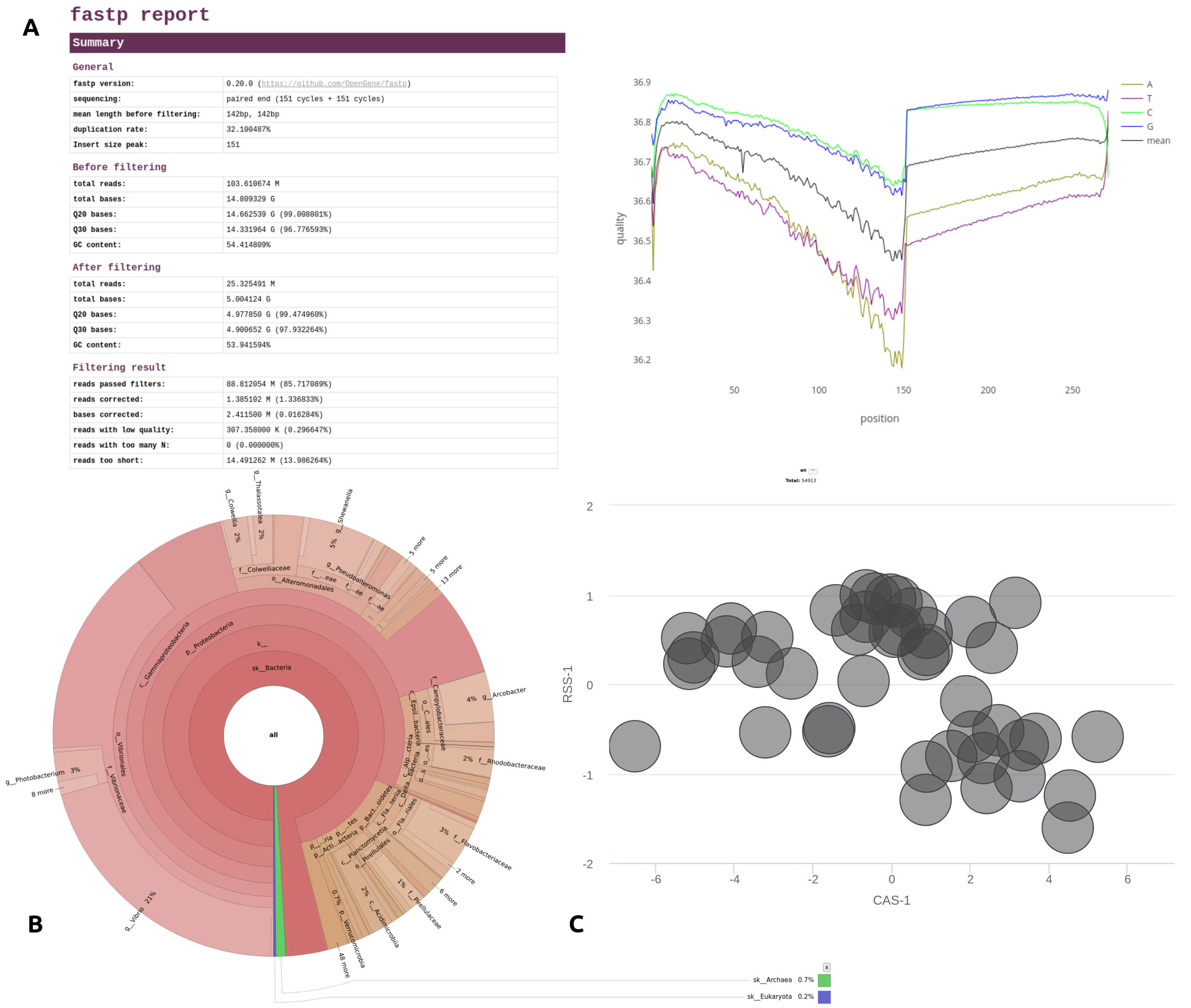

Figure 2. Visualisation of metaGOflow's main output. A. Raw data are first filtered and only high quality sequences are analysed further in the next steps. An `.html` file with the report of the merged reads is produced. Here, an excerpt of this report is shown: reads' statistics before and after filtering (left), ATGC chart with the quality of each base cycle-after-cycle for the merged reads (right) B. The taxonomic inventory step returns Molecular Operational Taxonomic Units (mOTUs) and the taxonomic composition based on the Large Sub Unit (LSU) and the Small Sub Unit (SSU) genes. Here, the taxonomic composition is represented by a Krona interactive visualization. C. The functional annotation step returns text files with the Gene Ontology (GO), KEGG, InterProScan, and Pfam terms retrieved. The retrieved GO terms are presented using Navigo [38], the Co-occurrence Association Score (CAS-1) and the Relevance Semantic Similarity (RSS-1). The Gene prediction step returns a `.ffn` and a `.faa` file while the assembly step a `.fasta` file including the contigs retrieved. The main output of the provenance feature is the `ro-crate-metedata.json` file.

For the visualization of each annotation type there is a great number of software; indicatively, in Figure 2C, the Co-occurrence Association Score (CAS) scores of the GO terms found in the sample are plotted against their Relevance Semantic Similarity (RSS) scores, which quantify the frequency of co-occurring GO terms within the gene annotations in the GOA database, as described in Navigo [38].

Building RO-Crates

An RO-Crate is created automatically by the workflow to store the data products of the aforementioned steps, along with the MetaGOflow run associated metadata (including the user set parameters, the version and the source of the workflow used). To this end, the `rocrate` Python library [70, 71] is used. As mentioned, an RO-Crate object is accompanied by a `JSON-LD` file (called `ro-crate-metadata.json`), part of which is shown in Figure 3, which includes the descriptions of both input and output files.
A thorough list of the metaGOflow's data products along with their descriptions can be found in the *Description of metaGOflow's data products* page of the manual. Supporting documentation, related to some of the software tools invoked by metaGOflow, is also provided to support the interpretation of the data products.

Parameters tuning

The `config.yml` file is the interface between the user and the pipeline. Through this file, the user sets which steps to perform, a number of parameters related to the idiosyncrasy of each experiment, as well as parameters that may affect the time efficiency of metaGOflow to a great extent (i.e., number of chunks). Further, metaGOflow supports inline arguments describing technical aspects of how to run, e.g. which containerization technology should be used. A thorough description of these parameters, as well as best practices and rules-of-thumb, are available at metaGOflow's manual on the *Arguments and parameters* page.

Table 1. Comparison of the main features and implementation of pipelines similar to metaGOflow.

Category	Feature	MetaWRAP	ATLAS	nf-core/taxprofiler	nf-core/funcscan	metaGOflow
Pre-processing	Quality control	fastqc	-	fastp, falco	-	fastp
	Filtering	Trim Galore	BBTools	porechop, fastp, bbduk, prinseq++, Filtlong	-	fastp
Taxonomy	Host-read removal	bmtagger	-	Bowtie2 for short reads and minimap2 for long reads	-	-
	Taxonomy assignment of rRNA genes	-	-	-	-	mOTUs, MAPseq
	Taxonomic assignment of reads and or contigs	kraken, kraken2	-	Kraken2, DIAMOND, mOTUs, MetaPhlAn3, MALT	-	-
	Taxonomic assignment of bins	TAXATOR-TK	GTDB-tk	-	-	-
Assembly	Short read assembly	metaspades and/or MEGAHIT	MEGAHIT	-	-	MEGAHIT
	Hybrid assembly	-	Yes	-	-	-
	Group-wise co-assembly	Yes	Yes	-	-	-
BINs-MAGs	Genome binning	metaBAT2, MaxBin2, CONCOCT	metabat2, maxbin2	-	-	-
	Bin refinement	Binning-refiner	-	DAS Tool	-	-
Annotation	Gene prediction	-	-	prodigal	-	FragGeneScan
	Functional annotation	prokka (using the bins)	eggNOG	-	hAMRonization, AMPcombi, comBGC.py	InterProScan, eggNOG, hmmsearch
	Ontologies	-	eggNOG	-	-	KEGG, GO, pfam, eggNOG, InterPro
FAIR-ness	keeping track of sample's metadata	-	-	-	-	Yes
	output as RO-Crate	-	-	-	-	Yes
	workflow provided through containers	-	-	-	Yes	Yes
Architecture	workflow manager	-	snakemake	nextflow	nextflow	cwl

Use case

To demonstrate metaGOflow and its key features, the analysis of a sediment and a water column sample from EMO BON was performed. As mentioned in the EMO BON handbook [72] and the EMO BON paper [14], DNA extraction, cleaning, library preparation and sequencing is performed at a centralised facility to minimize biases and maximize consistency in sequence quality. DNA extraction is performed using commercially available kits, to minimise deviations among samples. The samples were randomly chosen from two different stations but are considered to be representative EMO BON data. Moreover, an already publicly available marine metagenome sample from the Tara Oceans expedition [73], with size (in Gb) similar to those of the EMO BON data, was also analysed. All steps of metaGOflow were performed for each of these samples and the computational time (in hours), and the maximum memory (RAM, in GB) are reported in Table 2. Additionally, to demonstrate the applicability of metaGOflow for all types of shotgun metagenomic data, it was implemented for the analysis of a fish gut and a human gut metagenomic sample. All five samples were sequenced in different platforms: NovaSeq (EMO BON), HiSeq 2000 (Tara Oceans), BGISEQ-500 (fish gut), NextSeq 550 (human gut). The metaGOflow results for the gut samples are included in the zenodo repository [74] and the respective statistics are given in Supplementary Table 1.

Raw sequences were preprocessed using 130 bp as the minimum length of the reads and at least 30 bp of overlap for the merging step for the 2 EMO BON samples. In case of the Tara Oceans sample, a minimum length of 108 bp was used as the sequences were shorter. The pre-processing and the taxonomic inventory step lasted about from 10 to 24 hours. By allocating a computing node similar to the one used for the use case, taxonomic inventories from at least 300 metagenomes could be produced per year, based on the results from the EMO BON samples.

For the assembly step, a minimum contig length of 200 bp was used for all the samples. The assembly of the reads using the MEGAHIT algorithm was performed in less than 2 hours, while the maximum memory required was less than 10Gb which is at least one order of magnitude less than what other software, e.g. metaSPAdes, would require. The large number of contigs returned suggests one could aim for a higher minimum contig length. For example, using a minimum contig length of 500 bp for the Tara Oceans sample, the number of contigs was decreased from 102,343 (Table 2) to 34,426 and the required time was about 30 minutes.

The gene calling and the functional annotation steps were those requiring the most computing resources, as expected. For each of the three samples, it took about 4 days to complete these steps, with the InterProScan part being the most computationally expensive with respect to both time and memory. In order for metaGOflow to exploit the available computing resources in an optimal way, the user is strongly advised to follow the "Improving performance" instructions of InterProScan and set the relative arguments accordingly.

A summary of the metaGOflow outputs and their respective size for this use case is shown in Table 3. A visual representation of the detailed results (quality control report, taxonomic inventories, functional annotations) of the workflow can be found through this GitHub page[75]. An example of the complete data product of metaGOflow, packed in a RO-Crate, can be found through this Zenodo repo [74]. For the EMO BON samples, the default configuration files `config.yml` were used; for the Tara Oceans sample, the `config.yml` is included in the respective RO-crate object, which is available in the Zenodo repository.

Based on the scientific questions to be addressed, several types of downstream statistical analysis using the metaGOflow data products might be performed. Most of these statistical approaches are not specific for the analysis of metagenomic datasets *per se* [76]. Contrary, they are well established in several research communities: microbial ecologists, microbiologists, medical scientists. However, the nature of the metagenomic data lead to several challenges, such as the "compositional effect" that need to be dealt to the best possible extent [77, 78].


```
{
    "@id": "results/functional-annotation/stats/interproscan.stats",
    "@type": "File",
    "encodingFormat": "text/plain",
    "name": "InterProScan summary statistics"
},
{
    "@id": "results/functional-annotation/stats/go.stats",
    "@type": "File",
    "encodingFormat": "text/plain",
    "name": "Geno Ontology summary statistics"
},
{
    "@id": "results/functional-annotation/stats/ko.stats",
    "@type": "File",
    "encodingFormat": "text/plain",
    "name": "Kegg Ontology summary statistics"
},
{
    "@id": "results/functional-annotation/stats/pfam.stats",
    "@type": "File",
    "encodingFormat": "text/plain",
    "name": "Pfam summary statistcs"
},
{
    "@id": "results/functional-annotation/stats/orf.stats",
    "@type": "File",
    "encodingFormat": "text/plain",
    "name": "ORF summary statistics"
},
{
    "@id": "https://www.apache.org/licenses/LICENSE-2.0",
    "@type": "CreativeWork",
    "identifier": "https://spdx.org/licenses/Apache-2.0.html",
    "name": "Apache License 2.0"
},
```


Figure 3. Part of the `ro-crate-metadata.json` file describing the metaGOflow output files.

Discussion & conclusions

Metagenomic applications include different procedures and require expertise in different topics, from field sampling, to lab analyses, to sequencing [79]. This inevitably leads to delays in raw data production, let alone usable scientific results. On top of that, metagenomic raw data are not directly usable as they require time-consuming and computationally-demanding processing as well as specialized bioinformatics expertise [79, 76]. For EMO BON and other GOs to produce applicable and fit-for-purpose data, it is of huge importance that quality-controlled and standardised data, as well as informative data products, are made rapidly available. The disen-

Table 2. Computing requirements for the analysis of a sediment and a water column EMO BON sample as well as a Tara Oceans water sample, using metaGOflow in a "fat" node of the Zorba HPC.

workflow step(s)	computational time (hours)			memory (max RAM in Gb)		
	EB sediment	EB water	TO water	EB sediment	EB water	TO water
prepr. & taxon. invent. (Steps 1&2)	14.5	12.6	26.4	4.55	4.65	4.15
assembly (Step 3)	1.6	1.22	0.4	8.8	4.38	2.7
gene calling & funct. annot. (Steps 4&5)	98.7	92.4	84.2	205.1	188.6	155.4

EB: EMO BON, TO: Tara Oceans.

Table 3. metaGOflow results for the two EMO BON samples (marine sediment and a water column) and the Tara Oceans (seawater) sample.

product	EMO BON sediment	EMO BON water	Tara Oceans water
total reads (M)	51.8	44.0	36.5
filtered reads (M)	33.2	28.2	19.9
SSU	438	361	345
LSU	719	469	444
contigs	348,405	338,467	102,343
Reads with predicted CDS (M)	32.4	27.4	18.8
Pred. CDS* with IPS match (M)	9.9	9.4	5.2
Pred. CDS with GO match (M)	5.4	5.6	3.2
Pred. CDS with Pfam match (M)	9.3	8.9	4.9
Pred. CDS with KO match (M)	1.0	1.15	0.5

M: millions, *CDS: Coding Sequences

tanglement of the analyses from technical expertise and extensive computing infrastructures will allow the direct generation of meaningful data products, even by non-experts. There is a paramount added value to the provision of preliminary results and data products (i.e. taxonomic inventories) from metagenomic GO samples as it can lead to the full exploitation of the data, including enhanced and timely decision-making and successful environmental quality monitoring of the marine environment.

metaGOflow was developed with the ultimate objective to build a distributed workflow for analyses of marine metagenomic data generated by GOs such as EMO BON. The modular notion of metaGOflow allows us to perform the steps related to the taxonomy inventories and at a later point investigate the functional potential of a sample. Taxonomic inventories, essential for the case of GOs, are retrieved in a few hours. The functional annotation, as implemented, is highly time consuming compared to any other step of the workflow. That is mostly because of the InterProScan implementation; the vast amount of sequences but also the `standalone` module with which the scan is performed, lead to long single threaded processes. However, once the `clustermode` will be as fault tolerant as the `standalone`, metaGOflow will adopt it. On top of that, optimisations on the implementation of the InterProScan step would decrease further the total time for the complete analysis. MEGAHIT provides an assembly of the reads that it can then be used with the corresponding MGnify workflow for further analysis. Ultimately, using the parallel option of the `cwltool` combined with HPC environments and its modular notion, metaGOflow enables the effective, on time and valid, analysis of GOs data.

metaGOflow packages all its output, the workflow's metadata as well as the user's settings, in RO-crates, which is a novel feature in metagenomics bioinformatics analysis pipelines, to the best of our knowledge and as mentioned in Table 1. This novelty in the workflow's implementation allows the EMO BON community to access all data products, along with details on the employed methods, in a machine-readable way, either directly (see Zenodo example [74]) or through portals such as MGnify. Thus, it is now far easier for data and data products to be re-used for meta-analyses, but also to be exploited by data integration approaches [80, 81].

CWL, i.e. the language that the workflow is built on, has certain drawbacks. Among them, the requirement for explicit input-output declarations, the fact that the Javascript `ExpressionTools` may affect the portability of the workflow, and mainly being a data-driven "dataflow", means that handy control workflow patterns (e.g., loops) cannot be used [82]. However, some other features of the language, i.e. its modularity and its consistency when combined with containerization technologies, allowed us to build on top of the well-established MGnify environment; thus, metaGOflow enables the robust, standardized and fast-enough analysis of GO data. By all means, other workflow managers, such as Nextflow [28], may also support such community efforts. Toil [83] and similar technologies will be investigated for better exploitation of the provided computing resources, as well as cloud-based implementations of the workflow. The future integration of metaGOflow in e-infrastructures will be also considered.

The need for different approaches in the analysis of the shotgun metagenomics raw data has been well established [76]. metaGOflow's data products, like the output of any bioinformatics analysis of shotgun metagenomics data [84], may be explored in various ways through a great range of downstream analysis. Questions about key taxa in a sample or in a group of samples, about essential metabolic pathways that characterize a sample or a group of samples compared with others and so on, they can now be addressed using the findings of shotgun metagenomics analysis as input. In [85], Liu et al. distinguish the possible downstream analysis in *"overall"*, exploring differences in alpha/beta- diversity and taxonomic composition in a feature table, and *"details analysis"*, identifying biomarkers via comparison (using correlation and/or network analysis, machine learning etc.).

metaGOflow adds to a list of similar approaches such as nf-core/mag [86], metaWRAP [22], MG-RAST [32], JGI-IMG [34], bioBakery 3 (MetaPhlan 3) [23]. metaGOflow highlights the potential that modern workflow managers and containerization technologies support for building workflows upon workflows. Regarding raw data deriving from GOs, metaGOflow facilitates data generation , and, subsequently, interpretation of times-series biodiversity data, thus granting valuable insights to the scientific community and building a solid foundation for long-term sustainable and high-value data outputs. Long-term sustainability is assured by the FAIRness of the outputs and the strategic support of the EMBRC-ERIC infrastructure. Moreover, even if it was initially developed to address the specific needs of a GO project such as EMO BON, metaGOflow is overall a user-friendly flexible workflow that can be broadly used for one-sample-at-a-time analysis of shotgun metagenomics data.

Availability of source code and requirements

- Project name: metaGOflow: A workflow for marine Genomic Observatories data analysis
- Project home page: https://github.com/emo-bon/MetaGOflow
- Manual page: https://metagoflow.readthedocs.io
- WorkflowHub: https://workflowhub.eu/workflows/384
- RRID: SCR_023674
- biotools id: metagoflow
- Operating system(s): Unix
- Programming language: Common Workflow Language (CWL)
- Other requirements: Docker or Singularity engines. Node.js is required in cases where Docker is not available.
- License: Apache License 2.0. For third-party components separate licenses apply. Any restrictions to use by non-academics: licence needed.

Availability of supporting data and materials

Snapshots of our code and other data further supporting this work are openly available in the GigaScience repository, GigaDB 102443 [87]. All the raw sequence files of this study are available at ENA [43]:

- EMO BON super study accession number PRJEB51688 [88]
- EMO BON marine sediment sample: run accession number ERS14961254 [89], study accession number PRJEB51652 [90]
- EMO BON water column sample: run accssion number ERS14961281 [91] study accession number PRJEB51664 [92]
- Tara Oceans sample: run accession number ERR599171 [93], study accession number PRJEB402 [94]

Declarations

List of abbreviations

- CDS: Coding Sequences
- CWL: Common Workflow Language
- EMBRC: European Marine Biological Resource Centre
- EMO BON: European Marine Omics Biodiversity Observation Network
- ENA: European Nucleotide Archive
- GO terms: Gene Ontology terms
- GOs: Genomic Observatories
- HPC: High Performance Computing
- LSU: Large Sub Unit
- OSD: Ocean Sampling Day
- RO-Crate: Research Object Crate
- SSU: Small Sub Unit

Ethical Approval

Not applicable.

Consent for publication

Not applicable.

Competing Interests

M.B., L.R. and R.D.F. are members of the MGnify group that is part of the ELIXIR infrastructure [95]. The authors declare that they have no other competing interests.

Funding

This project has received funding from the European Union's Horizon 2020 research and innovation programme under grant agreement No 824087, under the 1st EOSC-Life Digital Life Sciences Open Call (Project ID 14325) and by the European Marine Biological Resource Centre - European Research Infrastructure Consortium (EMBRC-ERIC), which is part of the European Strategy Forum on Research Infrastructures (ESFRI). M.B. and L.R. were supported by EBML core funds.

Author's Contributions

Conceptualization: C.J.C., R.D.F., C.P.; Project Administration: C.P., A.P., H.Z.; Investigation: H.Z., M.B., S.N., G.D.M., J.M.; Formal Analysis: H.Z.; Software: H.Z., M.B., S.N., J.M., C.J.C.; Methodology: H.Z., S.N., K.E., E.C.; Validation: H.Z., C.P., I.S.; Data Curation: I.S., K.E., C.P., H.Z.; Resources: I.S., R.D.F., L.R., C.J.C., E.P.; Funding Acquisition: C.P., G.K., C.J.C., H.Z., R.D.F.; Writing - Original Draft Preparation: H.Z., C.P.; Writing - Review & Editing: all; Visualization: H.Z.

Acknowledgements

This research was supported in part through computational resources provided by IMBBC (Institute of Marine Biology, Biotechnology and Aquaculture) of the HCMR (Hellenic Centre for Marine Research). Funding for establishing the IMBBC HPC has been received by the MARBIGEN (EU Regpot) project, LifeWatchGreece RI and the CMBR (Centre for the study and sustainable exploitation of Marine Biological Resources) RI. This study received Portuguese national funds from FCT - Foundation for Science and Technology through project UIDB/04326/2020, UIDP/04326/2020 and LA/P/0101/2020, and from the operational programmes CRESC Algarve 2020 and COMPETE 2020 through projects EMBRC.PT ALG-01-0145-FEDER-022121 and BIODATA.PT ALG-01-0145-FEDER-022231 to C.J.C and G.D.M. This work received Computational Time to HPC infrastructures and scientific and technical support from the high-level support team at NCC-Greece. The financial support from the EuroHPC-JU Project 101101903—EuroCC 2 project of the European Commission is acknowledged. Parts of the runs were performed on the MeluXina machine within the project with ID: EHPC-DEV-2022D10-062. The acquisition and operation of the EuroHPC supercomputer is funded jointly by the EuroHPC Joint Undertaking, through the European Union's Connecting Europe Facility and the Horizon 2020 research and innovation programme, as well as the Grand Duché du Luxembourg.

References

1. Louca S, Parfrey LW, Doebeli M. Decoupling function and taxonomy in the global ocean microbiome. Science 2016;353(6305):1272–1277.
2. Doney SC, Ruckelshaus M, Emmett Duffy J, Barry JP, Chan F, English CA, et al. Climate change impacts on marine ecosystems. Annual review of marine science 2012;4:11–37.
3. Chen J, McIlroy SE, Archana A, Baker DM, Panagiotou G. A pollution gradient contributes to the taxonomic, functional, and resistome diversity of microbial communities in marine sediments. Microbiome 2019;7(1):1–12.
4. Caruso G, La Ferla R, Azzaro M, Zoppini A, Marino G, Petochi T, et al. Microbial assemblages for environmental quality assessment: knowledge, gaps and usefulness in the European Marine Strategy Framework Directive. Critical reviews in microbiology 2016;42(6):883–904.
5. Caruso G, Azzaro M, Caroppo C, Decembrini F, Monticelli LS, Leonardi M, et al. Microbial community and its potential as descriptor of environmental status. ICES Journal of Marine Science 2016;73(9):2174–2177.
6. Liu X, Ashforth E, Ren B, Song F, Dai H, Liu M, et al. Bioprospecting microbial natural product libraries from the marine environment for drug discovery. The Journal of Antibiotics 2010;63(8):415–422.
7. Glasl B, Webster NS, Bourne DG. Microbial indicators as a diagnostic tool for assessing water quality and climate stress in coral reef ecosystems. Marine Biology 2017;164(4):1–18.
8. Kopf A, Bicak M, Kottmann R, Schnetzer J, Kostadinov I, Lehmann K, et al. The ocean sampling day consortium. Gigascience 2015;4(1):1–5.
9. Duarte CM. Seafaring in the 21st century: the Malaspina 2010 circumnavigation expedition. Limnology and Oceanography Bulletin 2015;.
10. Sunagawa S, Acinas SG, Bork P, Bowler C, Eveillard D, Gorsky

G, et al. Tara Oceans: towards global ocean ecosystems biology. Nature Reviews Microbiology 2020;18(8):428–445.
11. Zayed AA, Wainaina JM, Dominguez-Huerta G, Pelletier E, Guo J, Mohssen M, et al. Cryptic and abundant marine viruses at the evolutionary origins of Earth's RNA virome. Science 2022;376(6589):156–162.
12. Sunagawa S, Coelho LP, Chaffron S, Kultima JR, Labadie K, Salazar G, et al. Structure and function of the global ocean microbiome. Science 2015;348(6237):1261359.
13. Yelton AP, Acinas SG, Sunagawa S, Bork P, Pedrós-Alió C, Chisholm SW. Global genetic capacity for mixotrophy in marine picocyanobacteria. The ISME journal 2016;10(12):2946–2957.
14. Santi I, Beluche O, Beraud M, Buttigieg P, Casotti R, Cox C, et al. European marine omics biodiversity observation network: a strategic outline for the implementation of omics approaches in ocean observation. Frontiers in Marine Science 2023;10:1118120.
15. Advancing ocean science | EMBRC;. https://www.embrc.eu/.
16. Buck M, Hamilton C. The Nagoya Protocol on access to genetic resources and the fair and equitable sharing of benefits arising from their utilization to the Convention on Biological Diversity. Review of European Community & International Environmental Law 2011;20(1):47–61.
17. GSC, Genomic Standards Consortium;. https://genomicsstandardsconsortium.github.io/gensc.github.io//.
18. Kottmann R, Gray T, Murphy S, Kagan L, Kravitz S, Lombardot T, et al. A standard MIGS/MIMS compliant XML Schema: toward the development of the Genomic Contextual Data Markup Language (GCDML). Omics a journal of integrative biology 2008;12(2):115–121.
19. Samuel RM, Meyer R, Buttigieg PL, Davies N, Jeffery NW, Meyer C, et al. Toward a Global Public Repository of Community Protocols to Encourage Best Practices in Biomolecular Ocean Observing and Research. Frontiers in Marine Science 2021;p. 1488.
20. Decade O, Better Biomolecular Ocean Practices; 2022. https://oceandecade.org/actions/better-biomolecular-ocean-practices/.
21. Tamames J, Cobo-Simón M, Puente-Sánchez F. Assessing the performance of different approaches for functional and taxonomic annotation of metagenomes. BMC genomics 2019;20(1):1–16.
22. Uritskiy GV, DiRuggiero J, Taylor J. MetaWRAP—a flexible pipeline for genome-resolved metagenomic data analysis. Microbiome 2018;6(1):1–13.
23. Beghini F, McIver LJ, Blanco-Míguez A, Dubois L, Asnicar F, Maharjan S, et al. Integrating taxonomic, functional, and strain-level profiling of diverse microbial communities with bioBakery 3. Elife 2021;10:e65088.
24. Ewels PA, Peltzer A, Fillinger S, Patel H, Alneberg J, Wilm A, et al. The nf-core framework for community-curated bioinformatics pipelines. Nature biotechnology 2020;38(3):276–278.
25. Straub D, Blackwell N, Langarica-Fuentes A, Peltzer A, Nahnsen S, Kleindienst S. Interpretations of environmental microbial community studies are biased by the selected 16S rRNA (gene) amplicon sequencing pipeline. Frontiers in Microbiology 2020;11:550420.
26. Merkel D. Docker: lightweight linux containers for consistent development and deployment. Linux journal 2014;2014(239):2.
27. Kurtzer GM, Sochat V, Bauer MW. Singularity: Scientific containers for mobility of compute. PloS one 2017;12(5):e0177459.
28. Di Tommaso P, Chatzou M, Floden EW, Barja PP, Palumbo E, Notredame C. Nextflow enables reproducible computational workflows. Nature biotechnology 2017;35(4):316–319.
29. Mölder F, Jablonski K, Letcher B, Hall M, Tomkins-Tinch C, Sochat V, et al. Sustainable data analysis with Snakemake [version 1; peer review: 1 approved, 1 approved with reservations]. F1000Research 2021;10(33).
30. Zafeiropoulos H, Gioti A, Ninidakis S, Potirakis A, Paragkamian S, Angelova N, et al. 0s and 1s in marine molecular research: a regional HPC perspective. GigaScience 2021;10(8):giab053.
31. ATLAS C, Yamamoto S, Shapiro M, Virzi J, Werner M, Venturi M, et al. The simulation principle and performance of the ATLAS fast calorimeter simulation FastCaloSim. ATL-COM-PHYS-2010-838; 2010.
32. Keegan KP, Glass EM, Meyer F. MG-RAST, a metagenomics service for analysis of microbial community structure and function. In: Microbial environmental genomics (MEG) Springer; 2016.p. 207–233.
33. Mitchell AL, Almeida A, Beracochea M, Boland M, Burgin J, Cochrane G, et al. MGnify: the microbiome analysis resource in 2020. Nucleic acids research 2020;48(D1):D570–D578.
34. Chen IMA, Chu K, Palaniappan K, Pillay M, Ratner A, Huang J, et al. IMG/M v. 5.0: an integrated data management and comparative analysis system for microbial genomes and microbiomes. Nucleic acids research 2019;47(D1):D666–D677.
35. Meyer F, Fritz A, Deng ZL, Koslicki D, Lesker TR, Gurevich A, et al. Critical assessment of metagenome interpretation: the second round of challenges. Nature methods 2022;19(4):429–440.
36. Soiland-Reyes S, Sefton P, Crosas M, Castro LJ, Coppens F, Fernández JM, et al. Packaging research artefacts with RO-Crate. Data Science 2021;5(2):1–42.
37. Wilkinson MD, Dumontier M, Aalbersberg IJ, Appleton G, Axton M, Baak A, et al. The FAIR Guiding Principles for scientific data management and stewardship. Scientific data 2016;3(1):1–9.
38. Wei Q, Khan IK, Ding Z, Yerneni S, Kihara D. NaviGO: interactive tool for visualization and functional similarity and coherence analysis with gene ontology. Bmc Bioinformatics 2017;18(1):1–13.
39. Amstutz P, Crusoe MR, Tijanić N, Chapman B, Chilton J, Heuer M, et al., Common workflow language, v1. 0. figshare; 2016.
40. Language (CWL) CW, Home;. https://www.commonwl.org/.
41. pipeline-v5. MGnify; 2023. https://github.com/EBI-Metagenomics/pipeline-v5, original-date: 2019-05-24T11:11:51Z.
42. Release v.1.0.1: Merge pull request #37 from emo-bon/fix-bugs · emo-bon/MetaGOflow;. https://github.com/emo-bon/MetaGOflow/releases/tag/v.1.0.1.
43. Burgin J, Ahamed A, Cummins C, Devraj R, Gueye K, Gupta D, et al. The European Nucleotide Archive in 2022. Nucleic Acids Research 2023;51(D1):D121–D125.
44. Microbiome Informatics ENA fetch tool. MGnify; 2022. https://github.com/EBI-Metagenomics/fetch_tool, original-date: 2018-09-06T15:38:50Z.
45. metaGOflow: A workflow for marine Genomic Observatories' data analysis. EMO BON; 2023. https://github.com/emo-bon/MetaGOflow, original-date: 2021-10-15T13:40:02Z.
46. metagoflow GitHub wiki page;. https://github.com/emo-bon/MetaGOflow/wiki/Home.
47. Welcome to metaGOflow's documentation! — metaGOflow 0.1 documentation;. https://metagoflow.readthedocs.io/en/latest/.
48. Front Page; 2023. https://www.luxprovide.lu/.
49. Chen S, Zhou Y, Chen Y, Gu J. fastp: an ultra-fast all-in-one FASTQ preprocessor. Bioinformatics 2018;34(17):i884–i890.
50. Nawrocki EP, Eddy SR. Infernal 1.1: 100-fold faster RNA homology searches. Bioinformatics 2013;29(22):2933–2935.
51. Eddy Lab: Home;. http://eddylab.org/.
52. Matias Rodrigues JF, Schmidt TS, Tackmann J, von Mering C. MAPseq: highly efficient k-mer search with confidence estimates, for rRNA sequence analysis. Bioinformatics 2017;33(23):3808–3810.
53. Milanese A, Mende DR, Paoli L, Salazar G, Ruscheweyh HJ, Cuenca M, et al. Microbial abundance, activity and popula-

tion genomic profiling with mOTUs2. Nature communications 2019;10(1):1–11.

54. Ondov BD, Bergman NH, Phillippy AM. Interactive metagenomic visualization in a Web browser. BMC bioinformatics 2011;12(1):1–10.
55. Vollmers J, Wiegand S, Kaster AK. Comparing and evaluating metagenome assembly tools from a microbiologist's perspective-not only size matters! PloS one 2017;12(1):e0169662.
56. Li D, Liu CM, Luo R, Sadakane K, Lam TW. MEGAHIT: an ultra-fast single-node solution for large and complex metagenomics assembly via succinct de Bruijn graph. Bioinformatics 2015;31(10):1674–1676.
57. Nurk S, Meleshko D, Korobeynikov A, Pevzner PA. metaSPAdes: a new versatile metagenomic assembler. Genome Research 2017;27(5):824–834. http://genome.cshlp.org/content/27/5/824.abstract.
58. Rho M, Tang H, Ye Y. FragGeneScan: predicting genes in short and error-prone reads. Nucleic acids research 2010;38(20):e191–e191.
59. Jones P, Binns D, Chang HY, Fraser M, Li W, McAnulla C, et al. InterProScan 5: genome-scale protein function classification. Bioinformatics 2014;30(9):1236–1240.
60. Mitchell AL, Attwood TK, Babbitt PC, Blum M, Bork P, Bridge A, et al. InterPro in 2019: improving coverage, classification and access to protein sequence annotations. Nucleic acids research 2019;47(D1):D351–D360.
61. El-Gebali S, Mistry J, Bateman A, Eddy SR, Luciani A, Potter SC, et al. The Pfam protein families database in 2019. Nucleic acids research 2019;47(D1):D427–D432.
62. Haft DH, Selengut JD, Richter RA, Harkins D, Basu MK, Beck E. TIGRFAMs and genome properties in 2013. Nucleic acids research 2012;41(D1):D387–D395.
63. Sigrist CJ, De Castro E, Cerutti L, Cuche BA, Hulo N, Bridge A, et al. New and continuing developments at PROSITE. Nucleic acids research 2012;41(D1):D344–D347.
64. Ashburner M, Ball CA, Blake JA, Botstein D, Butler H, Cherry JM, et al. Gene ontology: tool for the unification of biology. Nature genetics 2000;25(1):25–29.
65. Huerta-Cepas J, Szklarczyk D, Heller D, Hernández-Plaza A, Forslund SK, Cook H, et al. eggNOG 5.0: a hierarchical, functionally and phylogenetically annotated orthology resource based on 5090 organisms and 2502 viruses. Nucleic acids research 2019;47(D1):D309–D314.
66. Cantalapiedra CP, Hernández-Plaza A, Letunic I, Bork P, Huerta-Cepas J. eggNOG-mapper v2: functional annotation, orthology assignments, and domain prediction at the metagenomic scale. Molecular biology and evolution 2021;38(12):5825–5829.
67. Eddy SR. Accelerated profile HMM searches. PLoS computational biology 2011;7(10):e1002195.
68. Aramaki T, Blanc-Mathieu R, Endo H, Ohkubo K, Kanehisa M, Goto S, et al. KofamKOALA: KEGG Ortholog assignment based on profile HMM and adaptive score threshold. Bioinformatics 2020;36(7):2251–2252.
69. Kanehisa M, Sato Y, Kawashima M, Furumichi M, Tanabe M. KEGG as a reference resource for gene and protein annotation. Nucleic acids research 2016;44(D1):D457–D462.
70. Soiland-Reyes S, Sefton P, Crosas M, Castro LJ, Coppens F, Fernández JM, et al. Packaging research artefacts with RO-Crate. Data Science 2022;5(2):97–138.
71. De Geest P, Droesbeke B, Eguinoa I, Gaignard A, Huber S, Leo S, et al., ro-crate-py. Zenodo; 2022. https://doi.org/10.5281/zenodo.6594974, cite as.
72. Santi I, Casotti R, Comtet T, Cunliffe M, Koulouri PY, Macheriotou L, et al. European Marine Omics Biodiversity Observation Network (EMO BON) Handbook (Version 1.0). EMBRC-ERIC; 2021.
73. Pesant S, Not F, Picheral M, Kandels-Lewis S, Le Bescot N, Gorsky G, et al. Open science resources for the discovery and analysis of Tara Oceans data. Scientific data 2015;2(1):1–16.
74. Zafeiropoulos H, Beracochea M, Ninidakis S, Exter K, Moro GD, Potirakis A, et al., metaGOflow: a workflow for the analysis of marine Genomic Observatories shotgun metagenomics data - use case. Zenodo; 2023. https://doi.org/10.5281/zenodo.8046421.
75. use cases;. https://data.emobon.embrc.eu/MetaGOflow/.
76. Quince C, Walker AW, Simpson JT, Loman NJ, Segata N. Shotgun metagenomics, from sampling to analysis. Nature biotechnology 2017;35(9):833–844.
77. Faust K, Sathirapongsasuti JF, Izard J, Segata N, Gevers D, Raes J, et al. Microbial co-occurrence relationships in the human microbiome. PLoS computational biology 2012;8(7):e1002606.
78. Friedman J, Alm EJ. Inferring correlation networks from genomic survey data. PLoS computational biology 2012;8(9):e1002687.
79. Bharti R, Grimm DG. Current challenges and best-practice protocols for microbiome analysis. Briefings in bioinformatics 2021;22(1):178–193.
80. Reimer LC, Sardà Carbasse J, Koblitz J, Ebeling C, Podstawka A, Overmann J. Bac Dive in 2022: the knowledge base for standardized bacterial and archaeal data. Nucleic Acids Research 2022;50(D1):D741–D746.
81. Zafeiropoulos H, Paragkamian S, Ninidakis S, Pavlopoulos GA, Jensen LJ, Pafilis E. PREGO: a literature and data-mining resource to associate microorganisms, biological processes, and environment types. Microorganisms 2022;10(2):293.
82. contributors TUoMUaB, Pitfalls and limitations · BioExcel Best Practice Guide: Creating workflows with Common Workflow Language; 2021. http://docs.bioexcel.eu/cwl-best-practice-guide/limitations.html.
83. Vivian J, Rao AA, Nothaft FA, Ketchum C, Armstrong J, Novak A, et al. Toil enables reproducible, open source, big biomedical data analyses. Nature biotechnology 2017;35(4):314–316.
84. Sharpton TJ. An introduction to the analysis of shotgun metagenomic data. Frontiers in plant science 2014;5:209.
85. Liu YX, Qin Y, Chen T, Lu M, Qian X, Guo X, et al. A practical guide to amplicon and metagenomic analysis of microbiome data. Protein & cell 2021;12(5):315–330.
86. Krakau S, Straub D, Gourlé H, Gabernet G, Nahnsen S. nf-core/mag: a best-practice pipeline for metagenome hybrid assembly and binning. NAR Genomics and Bioinformatics 2022;4(1):lqac007.
87. Zafeiropoulos H, Beracochea M, Ninidakis S, Exter K, Potirakis A, De Moro G, et al., Supporting data for "metaGOflow: a workflow for the analysis of marine Genomic Observatories shotgun metagenomics data. GigaScience Database; 2023. http://dx.doi.org/10.5524/102443.
88. EMO BON ENA super study record;.http://www.ebi.ac.uk/ena/data/view/PRJEB51688, accessed: 2023-09-01.
89. EMO BON sediment sample ENA run record;.http://www.ebi.ac.uk/ena/data/view/ERS14961254, accessed: 2023-09-01.
90. ENA study record for EMO BON Observatory Station BPNS (VLIZ, UGhent, RBINS, KULeuven) samples;.http://www.ebi.ac.uk/ena/data/view/PRJEB51652, accessed: 2023-09-01.
91. EMO BON water column sample ENA run record;.http://www.ebi.ac.uk/ena/data/view/ERS14961281, accessed: 2023-09-01.
92. ENA study record for EMO BON Observatory Station VB (IMEV) samples;.http://www.ebi.ac.uk/ena/data/view/PRJEB51664, accessed: 2023-09-01.
93. TARA OCEAN sample ENA run record;.http://www.ebi.ac.uk/ena/data/view/ERR599171, accessed: 2023-09-01.
94. TARA OCEAN ENA study record;.http://www.ebi.ac.uk/ena/data/view/PRJEB402, accessed: 2023-09-01.
95. ELIXIR;. https://elixir-europe.org/.

Manuscript

Click here to access/download;Manuscript;marine_GOs_CWL_workflow_v3

OXFORD (GIGA)^n SCIENCE

GigaScience, 2022, 1–10

doi: xx.xxxx/xxxx
Manuscript in Preparation
Technical Note

TECHNICAL NOTE

metaGOflow: a workflow for the analysis of marine Genomic Observatories shotgun metagenomics data

Haris Zafeiropoulos[1,2] *, Martin Beracochea[3] *, Stelios Ninidakis[1], Katrina Exter[4], Antonis Potirakis[1], Gianluca De Moro[5], Lorna Richardson[3], Erwan Corre[6], João Machado[5], Evangelos Pafilis[1], Georgios Kotoulas[1], Ioulia Santi[7,1], Robert D. Finn[3], Cymon J. Cox[5] and Christina Pavloudi[1,8] †

[1] Institute of Marine Biology, Biotechnology and Aquaculture (IMBBC), Hellenic Centre for Marine Research (HCMR), Former U.S. Base of Gournes P.O. Box 2214, 71003, Heraklion, Crete, Greece and [2] KU Leuven, Department of Microbiology, Immunology and Transplantation, Rega Institute for Medical Research, Laboratory of Molecular Bacteriology, 3000 Leuven, Belgium and [3] European Molecular Biology Laboratory, European Bioinformatics Institute (EMBL-EBI), Wellcome Genome Campus, Hinxton, Cambridge CB10 1SD, UK and [4] Flanders Marine Institute (VLIZ), Oostende, Belgium and [5] Centro de Ciências do Mar (CCMAR), Universidade do Algarve, Campus de Gambelas, 8005-139, Faro, Portugal and [6] CNRS, FR 2424, ABiMS Platform, Station Biologique de Roscoff (SBR), Roscoff, France and [7] European Marine Biological Resource Centre (EMBRC-ERIC), Paris, France and [8] Department of Biological Sciences, The George Washington University, District of Columbia, USA

* Corresponding authors: haris.zafeiropoulos@kuleuven.be & mbc@ebi.ac.uk
† Current affiliation: PSL Research University: EPHE-UPVD-CNRS, UAR CNRS 3278 Centre de Recherche Insulaire et Observatoire de l'Environnement (CRIOBE), France & Laboratoire d'Excellence "CORAIL", Centre de Recherche Insulaire et Observatoire de l'Environnement (CRIOBE), French Polynesia

Abstract

Background: Genomic Observatories (GOs) are sites of long-term scientific study that undertake regular assessments of the genomic biodiversity . The European Marine Omics Biodiversity Observation Network (EMO BON) is a network of GOs that conduct regular biological community samplings to generate environmental and metagenomic data of microbial communities from designated marine stations around Europe. The development of an effective workflow is essential for the analysis of the EMO BON metagenomic data in a timely and reproducible manner.
Findings: Based on the established MGnify resource we developed metaGOflow. metaGOflow supports the fast inference of taxonomic profiles from GO-derived data based on rRNA genes and their functional annotation using the raw reads. Thanks to the Research Object Crate (RO-Crate) packaging, relevant metadata about the sample under study, and the details of the bioinformatics analysis it has been subjected to, are inherited to the data product while its modular implementation allows running the workflow partially. The analysis of two EMO BON and one Tara Oceans samples was performed as a use case.
Conclusions: metaGOflow is an efficient and robust workflow that scales to the needs of projects producing big metagenomic data such as EMO BON. It highlights how containerization technologies along with modern workflow languages and metadata package approaches can support the needs of researchers when dealing with ever-increasing volumes of biological data. Despite being initially oriented to address the needs of EMO BON, metaGOflow is a flexible and easy-to-use workflow that can be broadly used for one-sample-at-a-time analysis of shotgun metagenomics data.

Key words: shotgun metagenomics; MGnify; Common Workflow Language (CWL); containers; provenance; RO-Crate

Compiled on: September 8, 2023.
Draft manuscript prepared by the author.

Introduction

It is well established that microbial assemblages support multiple ecosystem services and that microbial community profiling using metagenomics methods can help elucidate the mechanisms that govern the structure of these communities and their interactions with the environment [1]. The community composition and structure of marine microbiome is directly correlated with environmental quality [2, 3]. Indeed, the quality of a marine microbial environment (e.g. a marine sediment) can impact the food chain [4] through the physical and chemical effects of secondary metabolites [5]. In addition, secondary metabolites produced by microorganisms may also become targets for bio-prospecting in medicine and industry [6]. Monitoring the changes in microbial community composition and function due to climate change-related stressors, such as ocean acidification or increases in temperature and UV absorption, can provide insights on ecosystem function, health, and resilience [7].

Pioneering research programmes such as the Ocean Sampling Day (OSD) [8], Malaspina circumnavigation expedition [9], and Tara Oceans [10], have been instrumental in collecting large series' of marine genomic samples from sites around the globe. The analysis of data resulting from these studies has greatly increased our understanding of the importance, the role, and the mechanisms governing microbial communities in some of the most common, sensitive or threatened marine environments [11, 12, 13]. EMO BON [14], a European Marine Biological Resource Centre (EMBRC-ERIC) [15] initiative, is designed to continue and expand this effort by regular bimonthly microbial genomic biodiversity samplings at designated marine coastal stations around the European coastline. In the first two years of the EMO BON (2021-2022) it is expected that more than 540 shotgun metagenomic data sets from water column and sediment samples will be generated from 17 European sites.

The ultimate success of GOs depends on the development and adoption of standards for sampling, metadata collection, sequencing, and data analysis. The provision of metadata relating to the raw sequence data, data products, and their analysis methods, are of high importance for interpretation and interoperability, and need to be accessible in both human- and machine-readable formats. Legislative framework, such as the Nagoya Protocol for Access and Benefit Sharing (ABS) [16], and community written frameworks, such as those developed by the Genomic Standards Consortium (GSC) [17, 18], as well as initiatives encouraging adherence to best practices, such as the Better Biomolecular Ocean Practices (BeBOP) project [19, 20], have all been key to providing agreed-upon standard that aim to fulfil these needs. Standard operating procedures and standardised methods of analysis enable the comparison of results among sites, through time, and among projects, without which, much of the value of the data for environmental assessment is lost.

Effective analysis of shotgun metagenomic data is time-consuming, especially regarding computational steps such as sequence assembly and annotation [21]. Moreover, microbial community profiling and functional analyses are most useful when samples are maximally comparable in space and time, and have been thereby treated using the same analytical procedures. To address the challenges that arise when analyzing metagenomic data, numerous workflows and pipelines have been developed. Notable pipelines include metaWRAP [22], bioBakery [23], and nf-core [24], which provides a collection of pipelines such as nf-core/ampliseq [25] and nf-core/taxprofiler [24]. Recently, containerization approaches (e.g., Docker [26], Singularity [27] etc.), along with workflow managers (e.g., Nextflow [28], Snakemake [29] etc.), have been widely used to a) address the complexity of the analysis, b) facilitate execution and reproducibility and c) distribute and share software to a broader audience [30]. nf-core and ATLAS [31] shotgun metagenomic analysis pipelines are examples of the implementation of such approaches.

Additionally, there are (data analysis) resources like MG-RAST [32], MGnify [33], and IMG/M [34] that come with their own distinct advantages and disadvantages.

The computing requirements for the analysis of the EMO BON data may exceed the computing capacity that a single research institute and/or a regional High Performance Computing (HPC) (i.e., Tier 2) systems can support using the available workflows. Indicatively, for a single dataset, software tools related to the retrieval of taxonomic profiles require up to 160 CPU hours and up to 100 GB of RAM [35]. Computing requirements for the functional annotation of shotgun reads are even higher. Nevertheless, timely provision of data and data products from GOs is of paramount importance to facilitate long-term ecological studies, to accelerate policy-making, and to directly assess the impact of anthropogenic effects on the marine environment.

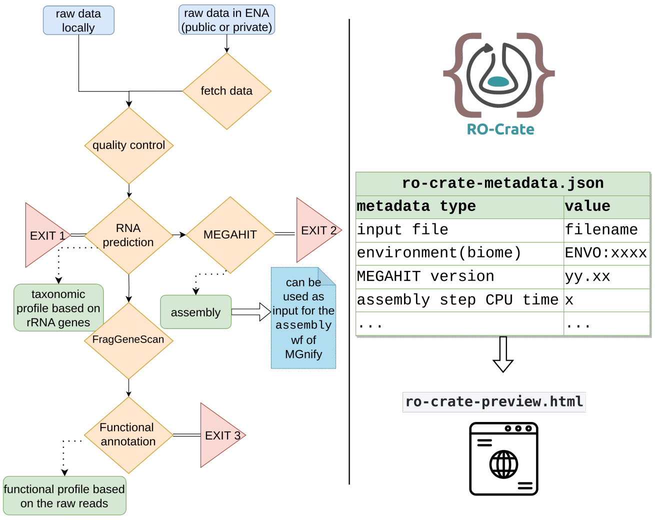

Figure 1. Schematic overview of metaGOflow, showing the main steps of the analysis along with their corresponding data products; the partial execution of the workflow is also shown by the potential exit points (left). Independent of the steps to be performed, once completed, an RO-Crate is built (right).

To address the challenges of analysing GO data in a timely and standardised framework we developed metaGOflow: a MGnify-based [33] computational workflow that implements the critical steps of a shotgun metagenomic bioinformatics analysis, and provides rich provenance metadata describing the data, data products, and workflow execution (Figure 1). The novel aspects of this workflow are mainly a) partial workflow execution; e.g. the user has the flexibility to choose whether to run the functional annotation sub-workflow or not, or even run it at a later point using the data products of the previous steps, b) the incorporation of an alternative assembler with a significantly lower computational cost as compared to the MGnfiy default one and c) the ultimate generation and verification of a Research Object (RO) crate ensuring the workflow's FAIRness. On top of that, several updates of the databases and tools invoked by MGnify have been performed.

metaGOflow consists of two basic concepts:

- an *analytical workflow* which provides taxonomic inventories and community gene function profiles of the samples as data products packaged in RO Crates [36],
- a *data provenance workflow* that generates extensive metadata and thereby provides compliance of the data, data products, and analytical procedures with FAIR data practices and the principles of Open Science, also packaged in the RO Crates [37, 19].

Implementation

Overview

The pillars around which metaGOflow [SCR_023674] has been built, namely containerisation technologies such as Docker [26] and Singularity [27], and the Common Workflow Language (CWL) [39, 40], ensure the workflow's ability to perform in different HPC and cloud computing platforms, following the MGnify example.

metaGOflow inherits the architecture of MGnify pipeline-v5 [41] and exploits several of the already containerized tools and the sub-workflows implemented in the MGnify pipeline. Several enhancements and upgrades allow metaGOflow to make use of the latest versions of the tools and databases invoked. metaGOflow makes extensive use of CWL *subworkflows* and *conditional* step execution to address the specific needs of the EMO BON project from a computing resources point of view.

For example, the user can run the workflow to only generate the taxonomic inventory of a sample. Then, at a later time and by using the output of the first analysis, the user can also generate the assembly of this sample's reads and/or their functional annotation. This flexibility in the workflow is essential as there are a considerable number of samples to be analysed (preferably in as short a period of time as possible), and the computing requirements, especially for the functional annotation step, can be substantial (see Table 2).

In its current version (v.1.0.1[42]), metaGOflow has 5 distinct steps. As in MGnify, metaGOflow analyses a single sample at a time (see Figure 1). The user may either provide locally stored raw data (`.fastq` files) or start the workflow by giving a European Nucleotide Archive (ENA)[43] run accession number. In the later case, metaGOflow invokes the `fetch_tool` [44] to retrieve the raw sequence files from ENA; if the data to be retrieved are held privately, the username and password of the associated ENA account are also requested. The user sets the steps of the workflow to be performed and provides values for certain tool parameters through a text-based configuration file (`config.yml`).

To enhance the FAIRness of the data products and of the bioinformatic analysis, metaGOflow data products are packages as RO-Crates: this allows the set of files to be semantically described, to be accompanied by the metadata that describe the precise steps of the workflow execution, the tools and the parameters used, and to flag the specific input and output files. This description is provided in a `JSON-LD` file following a particular (user-generated) profile. Along with the data products, the RO-crate contains information describing the version of the workflow *per se*, including the software and database versions that it uses.

A comparison of the main features of metaGOflow with other commonly used pipelines for shotgun metagenomic analysis is given in Table 1.

metaGOflow is available on GitHub [45]. A Continuous Integration/Continuous Deployment (CI/CD) workflow using GitHub Actions ensures the validity of the workflow's `cwl` main script and, therefore, of all its components. A thorough description of how to install and use metaGOflow, as well as common errors that might occur during the analysis of a sample can be found at its wiki page [46], as well as on its main documentation page [47]. The databases to be installed before using metaGOflow, require 160GB of storage and as a rule of thumb, the user should allocate 1TB of storage to perform a metaGOflow analysis.

The development and testing of metaGOflow was performed in the IMBBC HCMR "Zorbas" HPC [30] and at the HPC facility of CCMAR. Further testing was performed on the Luxembourg national supercomputer MeluXina [48]. The use case experiments (see Section 6) were performed in a "fat" node of the "Zorbas" HPC (2x Inter(R) Xeon(R) Gold 6230 CPU @ 2.10GHz 40 cores and 500 GB).

Step 1: Sequence preprocessing

Sequences are filtered and merged using `fastp` (version 0.20.0) [49]. Short, low quality, and non-merging sequences are removed and a series of statistical tests describing the quality of the sequencing are performed. An `.html` file returned by the `fastp` tool, provides visualizations of these statistics (see Figure 2A). The filtered sequences and the merged filtered sequences are returned as `.fasta` files.

Step 2: Taxonomy inventory

metaGOflow makes use of the `esl-sfetch` miniapp of the EASEL library (S.R. Eddy, unpublished) to index the filtered sequences and support fast sequence retrieval. Then `cmsearch`, an Infernal program [50, 51], is performed using the ribosomal and the non-coding RNA (ncRNA) Rfam covariance models (CM) (version v13.0) against the filtered sequences. Eventually, this is followed by taxonomic classification using `MAPseq` (v 1.2.3) [52] and the SILVA database (version 132) for the taxonomic classification of the SSU and the LSU sequences, while `mOTUs2` [53] quantifies both known and unknown taxa on the filtered sequences. metaGOflow automatically returns Krona plots (an interactive visualization approach of hierarchical data as multi-layered pie charts [54]) using the taxonomic assignments made for the SSU and LSU genes (see Figure 2B).

Step 3: Assembly

Shotgun metagenomic read assembly requires significant computing resources as discussed in Mitchell et. *al* [33] and in Vollmers et. *al* [55]. The extent of the computational "burden" depends heavily on the chosen algorithm. To be able to handle the vast amount of data produced by EMO BON in a timely manner, and since we aim more at unravelling biodiversity at the community, rather than at the individual (i.e. species), level, metaGOflow makes use of the MEGAHIT algorithm [56]. Longer contigs would be returned if e.g. metaSPAdes [57] was employed, but given metaGOflow's high pace data generation and analysis needs, the MEGAHIT algorithm seems a better match.

Step 4: Gene prediction on the reads

metaGOflow performs gene prediction using FragGeneScan (v1.20) [58] like MGnify. This step is a prerequisite for the functional annotation of the reads (Step). To partially run this step, the user needs to provide the merged filtered `.fasta` file, provided by the sequence preprocessing step.

Step 5: Functional annotation of the reads

metaGOflow focuses on the potential metabolic processes of the whole community rather than the processes of each individual species. Therefore, it performs functional annotation at the reads level. Using InterProScan (v.57-90) [59] metaGOflow annotates the reads with InterPro5 [60], PFam [61], TIGRFAM [62], ProSite patterns and profiles [63] and Gene Ontology (GO) [64] terms. Functional annotations are returned as text files. Both GO and GO Slim (available at geneontology.org) annotations are returned. EggNOG5 [65] annotation is also performed using to the eggnog-mapper (v2.1.8) [66]. Last, metaGOflow invokes the HMMER [67] tool along with the KOfam library [68] to get KEGG orthology annotations [69]. This step requires a significant amount of computing time.

To partially run this step, the user needs to provide the merged filtered `.fasta` file, provided by the sequence preprocessing step (Step 1) as well as the output of the gene prediction step (Step 4).

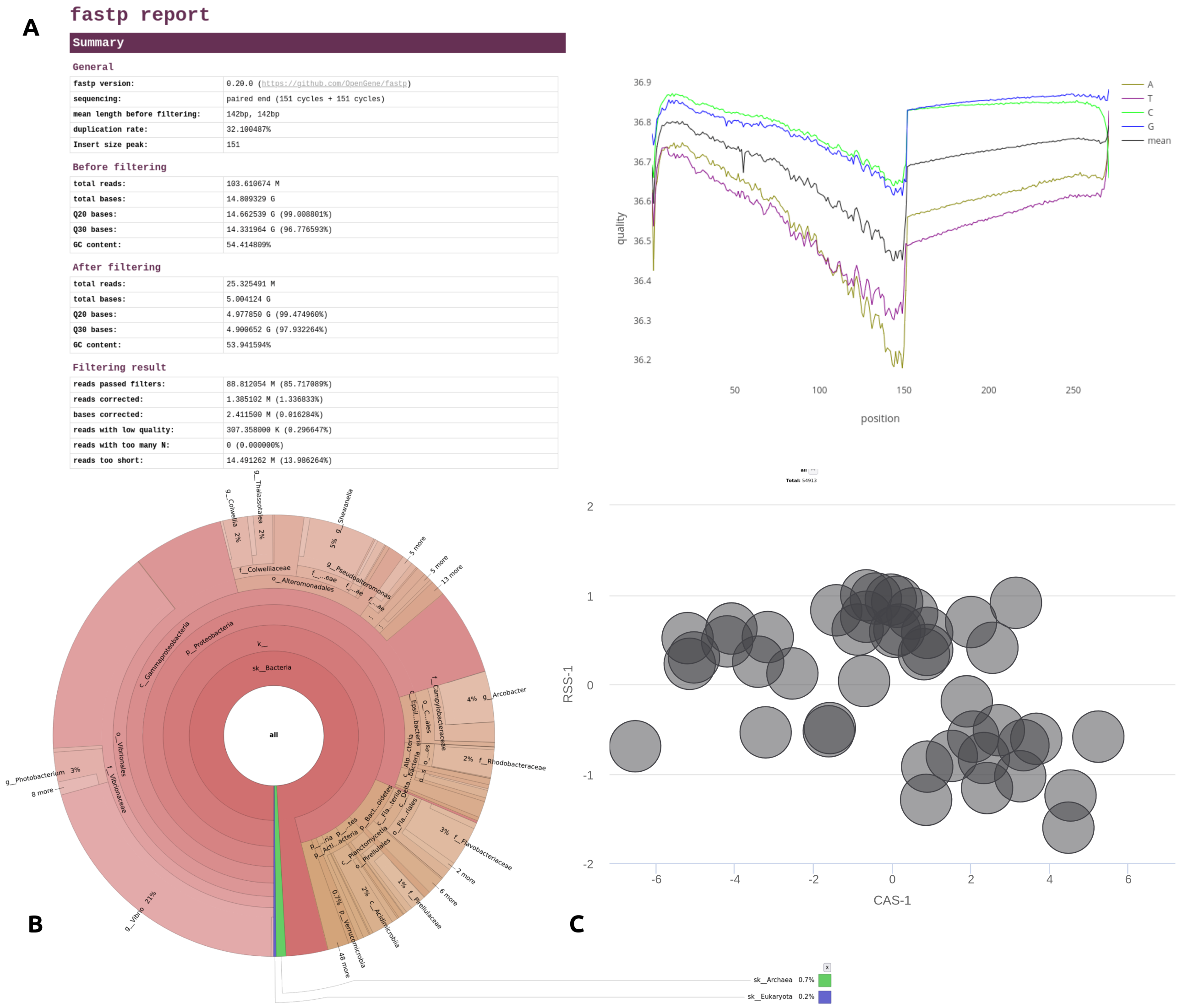

Figure 2. Visualisation of metaGOflow's main output. A. Raw data are first filtered and only high quality sequences are analysed further in the next steps. An `.html` file with the report of the merged reads is produced. Here, an excerpt of this report is shown: reads' statistics before and after filtering (left), ATGC chart with the quality of each base cycle-after-cycle for the merged reads (right) B. The taxonomy inventory step returns Molecular Operational Taxonomic Units (mOTUs) and the taxonomic composition based on the Large Sub Unit (LSU) and the Small Sub Unit (SSU) genes. Here, the taxonomic composition is represented by a Krona interactive visualization. C. The functional annotation step returns text files with the Gene Ontology (GO), KEGG, InterProScan, and Pfam terms retrieved. The retrieved GO terms are presented using Navigo [38], the Co-occurrence Association Score (CAS-1) and the Relevance Semantic Similarity (RSS-1). The Gene prediction step returns a `.ffn` and a `.faa` file while the assembly step a `.fasta` file including the contigs retrieved. The main output of the provenance feature is the `ro-crate-metedata.json` file.

For the visualization of each annotation type there is a great number of software; indicatively, in Figure 2C, the Co-occurrence Association Score (CAS) scores of the GO terms found in the sample are plotted against their Relevance Semantic Similarity (RSS) scores, which quantify the frequency of co-occurring GO terms within the gene annotations in the GOA database, as described in Navigo [38].

Building RO-Crates

An RO-Crate is created automatically by the workflow to store the data products of the aforementioned steps, along with the MetaGOflow run associated metadata (including the user set parameters, the version and the source of the workflow used). To this end, the `rocrate` Python library [70, 71] is used. As mentioned, an RO-Crate object is accompanied by a `JSON-LD` file (called `ro-crate-metadata.json`), part of which is shown in Figure 3, which includes the descriptions of both input and output files.
A thorough list of the metaGOflow's data products along with their descriptions can be found in the *Description of metaGOflow's data products* page of the manual. Supporting documentation, related to some of the software tools invoked by metaGOflow, is also provided to support the interpretation of the data products.

Parameters tuning

The `config.yml` file is the interface between the user and the pipeline. Through this file, the user sets which steps to perform, a number of parameters related to the idiosyncrasy of each experiment, as well as parameters that may affect the time efficiency of metaGOflow to a great extent (i.e., number of chunks). Further, metaGOflow supports inline arguments describing technical aspects of how to run, e.g. which containerization technology should be used. A thorough description of these parameters, as well as best practices and rules-of-thumb, are available at metaGOflow's manual on the *Arguments and parameters* page.

Table 1. Comparison of the main features and implementation of pipelines similar to metaGOflow.

Category	Feature	MetaWRAP	ATLAS	nf-core/taxprofiler	nf-core/funcscan	metaGOflow
Pre-processing	Quality control	fastqc	-	fastp, falco	-	fastp
	Filtering	Trim Galore	BBTools	porechop, fastp, bbduk, prinseq++, Filtlong	-	fastp
Taxonomy	Host-read removal	bmtagger	-	Bowtie2 for short reads and minimap2 for long reads	-	-
	Taxonomy assignment of rRNA genes	-	-	-	-	mOTUs, MAPseq
	Taxonomic assignment of reads and or contigs	kraken, kraken2	-	Kraken2, DIAMOND, mOTUs, MetaPhlAn3, MALT	-	-
	Taxonomic assignment of bins	TAXATOR-TK	GTDB-tk	-	-	-
Assembly	Short read assembly	metaspades and/or MEGAHIT	MEGAHIT	-	-	MEGAHIT
	Hybrid assembly	-	Yes	-	-	-
	Group-wise co-assembly	Yes	Yes	-	-	-
BINs-MAGs	Genome binning	metaBAT2, MaxBin2, CONCOCT	metabat2, maxbin2	-	-	-
	Bin refinement	Binning-refiner	-	DAS Tool	-	-
Annotation	Gene prediction	-	-	prodigal	-	FragGeneScan
	Functional annotation	prokka (using the bins)	eggNOG	-	hAMRonization, AMPcombi, comBGC.py	InterProScan, eggNOG, hmmsearch
	Ontologies	-	eggNOG	-	-	KEGG, GO, pfam, eggNOG, InterPro
FAIR-ness	keeping track of sample's metadata	-	-	-	-	Yes
	output as RO-Crate	-	-	-	-	Yes
	workflow provided through containers	-	-	-	Yes	Yes
Architecture	workflow manager	-	snakemake	nextflow	nextflow	cwl

Use case

To demonstrate metaGOflow and its key features, the analysis of a sediment and a water column sample from EMO BON was performed. As mentioned in the EMO BON handbook [72] and the EMO BON paper [14], DNA extraction, cleaning, library preparation and sequencing is performed at a centralised facility to minimize biases and maximize consistency in sequence quality. DNA extraction is performed using commercially available kits, to minimise deviations among samples. The samples were randomly chosen from two different stations but are considered to be representative EMO BON data. Moreover, an already publicly available marine metagenome sample from the Tara Oceans expedition [73], with size (in Gb) similar to those of the EMO BON data, was also analysed. All steps of metaGOflow were performed for each of these samples and the computational time (in hours), and the maximum memory (RAM, in GB) are reported in Table 2. Additionally, to demonstrate the applicability of metaGOflow for all types of shotgun metagenomic data, it was implemented for the analysis of a fish gut and a human gut metagenomic sample. All five samples were sequenced in different platforms: NovaSeq (EMO BON), HiSeq 2000 (Tara Oceans), BGISEQ-500 (fish gut), NextSeq 550 (human gut). The metaGOflow results for the gut samples are included in the zenodo repository [74] and the respective statistics are given in Supplementary Table 1.

Raw sequences were preprocessed using 130 bp as the minimum length of the reads and at least 30 bp of overlap for the merging step for the 2 EMO BON samples. In case of the Tara Oceans sample, a minimum length of 108 bp was used as the sequences were shorter. The pre-processing and the taxonomic inventory step lasted about from 10 to 24 hours. By allocating a computing node similar to the one used for the use case, taxonomic inventories from at least 300 metagenomes could be produced per year, based on the results from the EMO BON samples.

For the assembly step, a minimum contig length of 200 bp was used for all the samples. The assembly of the reads using the MEGAHIT algorithm was performed in less than 2 hours, while the maximum memory required was less than 10Gb which is at least one order of magnitude less than what other software, e.g. metaSPAdes, would require. The large number of contigs returned suggests one could aim for a higher minimum contig length. For example, using a minimum contig length of 500 bp for the Tara Oceans sample, the number of contigs was decreased from 102, 343 (Table 2) to 34, 426 and the required time was about 30 minutes.

The gene calling and the functional annotation steps were those requiring the most computing resources, as expected. For each of the three samples, it took about 4 days to complete these steps, with the InterProScan part being the most computationally expensive with respect to both time and memory. In order for metaGOflow to exploit the available computing resources in an optimal way, the user is strongly advised to follow the "Improving performance" instructions of InterProScan and set the relative arguments accordingly.

A summary of the metaGOflow outputs and their respective size for this use case is shown in Table 3. A visual representation of the detailed results (quality control report, taxonomic inventories, functional annotations) of the workflow can be found through this GitHub page[75]. An example of the complete data product of metaGOflow, packed in a RO-Crate, can be found through this Zenodo repo [74]. For the EMO BON samples, the default configuration files `config.yml` were used; for the Tara Oceans sample, the `config.yml` is included in the respective RO-crate object, which is available in the Zenodo repository.

Based on the scientific questions to be addressed, several types of downstream statistical analysis using the metaGOflow data products might be performed. Most of these statistical approaches are not specific for the analysis of metagenomic datasets *per se* [76]. Contrary, they are well established in several research communities: microbial ecologists, microbiologists, medical scientists. However, the nature of the metagenomic data lead to several challenges, such as the "compositional effect" that need to be dealt to the best possible extent [77, 78].

```
{
    "@id": "results/functional-annotation/stats/interproscan.stats",
    "@type": "File",
    "encodingFormat": "text/plain",
    "name": "InterProScan summary statistics"
},
{
    "@id": "results/functional-annotation/stats/go.stats",
    "@type": "File",
    "encodingFormat": "text/plain",
    "name": "Geno Ontology summary statistics"
},
{
    "@id": "results/functional-annotation/stats/ko.stats",
    "@type": "File",
    "encodingFormat": "text/plain",
    "name": "Kegg Ontology summary statistics"
},
{
    "@id": "results/functional-annotation/stats/pfam.stats",
    "@type": "File",
    "encodingFormat": "text/plain",
    "name": "Pfam summary statistcs"
},
{
    "@id": "results/functional-annotation/stats/orf.stats",
    "@type": "File",
    "encodingFormat": "text/plain",
    "name": "ORF summary statistics"
},
{
    "@id": "https://www.apache.org/licenses/LICENSE-2.0",
    "@type": "CreativeWork",
    "identifier": "https://spdx.org/licenses/Apache-2.0.html",
    "name": "Apache License 2.0"
},
```

Figure 3. Part of the `ro-crate-metadata.json` file describing the metaGOflow output files.

Discussion & conclusions

Metagenomic applications include different procedures and require expertise in different topics, from field sampling, to lab analyses, to sequencing [79]. This inevitably leads to delays in raw data production, let alone usable scientific results. On top of that, metagenomic raw data are not directly usable as they require time-consuming and computationally-demanding processing as well as specialized bioinformatics expertise [79, 76]. For EMO BON and other GOs to produce applicable and fit-for-purpose data, it is of huge importance that quality-controlled and standardised data, as well as informative data products, are made rapidly available. The disen-

Table 2. Computing requirements for the analysis of a sediment and a water column EMO BON sample as well as a Tara Oceans water sample, using metaGOflow in a "fat" node of the Zorba HPC.

workflow step(s)	computational time (hours)			memory (max RAM in Gb)		
	EB sediment	EB water	TO water	EB sediment	EB water	TO water
prepr. & taxon. invent. (Steps 1&2)	14.5	12.6	26.4	4.55	4.65	4.15
assembly (Step 3)	1.6	1.22	0.4	8.8	4.38	2.7
gene calling & funct. annot. (Steps 4&5)	98.7	92.4	84.2	205.1	188.6	155.4

EB: EMO BON, TO: Tara Oceans.

Table 3. metaGOflow results for the two EMO BON samples (marine sediment and a water column) and the Tara Oceans (seawater) sample.

product	EMO BON sediment	EMO BON water	Tara Oceans water
total reads (M)	51.8	44.0	36.5
filtered reads (M)	33.2	28.2	19.9
SSU	438	361	345
LSU	719	469	444
contigs	348,405	338,467	102,343
Reads with predicted CDS (M)	32.4	27.4	18.8
Pred. CDS* with IPS match (M)	9.9	9.4	5.2
Pred. CDS with GO match (M)	5.4	5.6	3.2
Pred. CDS with Pfam match (M)	9.3	8.9	4.9
Pred. CDS with KO match (M)	1.0	1.15	0.5

M: millions, *CDS: Coding Sequences

tanglement of the analyses from technical expertise and extensive computing infrastructures will allow the direct generation of meaningful data products, even by non-experts. There is a paramount added value to the provision of preliminary results and data products (i.e. taxonomic inventories) from metagenomic GO samples as it can lead to the full exploitation of the data, including enhanced and timely decision-making and successful environmental quality monitoring of the marine environment.

metaGOflow was developed with the ultimate objective to build a distributed workflow for analyses of marine metagenomic data generated by GOs such as EMO BON. The modular notion of metaGOflow allows us to perform the steps related to the taxonomy inventories and at a later point investigate the functional potential of a sample. Taxonomic inventories, essential for the case of GOs, are retrieved in a few hours. The functional annotation, as implemented, is highly time consuming compared to any other step of the workflow. That is mostly because of the InterProScan implementation; the vast amount of sequences but also the `standalone` module with which the scan is performed, lead to long single threaded processes. However, once the `clustermode` will be as fault tolerant as the `standalone`, metaGOflow will adopt it. On top of that, optimisations on the implementation of the InterProScan step would decrease further the total time for the complete analysis. MEGAHIT provides an assembly of the reads that it can then be used with the corresponding MGnify workflow for further analysis. Ultimately, using the parallel option of the `cwltool` combined with HPC environments and its modular notion, metaGOflow enables the effective, on time and valid, analysis of GOs data.

metaGOflow packages all its output, the workflow's metadata as well as the user's settings, in RO-crates, which is a novel feature in metagenomics bioinformatics analysis pipelines, to the best of our knowledge and as mentioned in Table 1. This novelty in the workflow's implementation allows the EMO BON community to access all data products, along with details on the employed methods, in a machine-readable way, either directly (see Zenodo example [74]) or through portals such as MGnify. Thus, it is now far easier for data and data products to be re-used for meta-analyses, but also to be exploited by data integration approaches [80, 81].

CWL, i.e. the language that the workflow is built on, has certain drawbacks. Among them, the requirement for explicit input-output declarations, the fact that the Javascript `ExpressionTools` may affect the portability of the workflow, and mainly being a data-driven "dataflow", means that handy control workflow patterns (e.g., loops) cannot be used [82]. However, some other features of the language, i.e. its modularity and its consistency when combined with containerization technologies, allowed us to build on top of the well-established MGnify environment; thus, metaGOflow enables the robust, standardized and fast-enough analysis of GO data. By all means, other workflow managers, such as Nextflow [28], may also support such community efforts. Toil [83] and similar technologies will be investigated for better exploitation of the provided computing resources, as well as cloud-based implementations of the workflow. The future integration of metaGOflow in e-infrastructures will be also considered.

The need for different approaches in the analysis of the shotgun metagenomics raw data has been well established [76]. metaGOflow's data products, like the output of any bioinformatics analysis of shotgun metagenomics data [84], may be explored in various ways through a great range of downstream analysis. Questions about key taxa in a sample or in a group of samples, about essential metabolic pathways that characterize a sample or a group of samples compared with others and so on, they can now be addressed using the findings of shotgun metagenomics analysis as input. In [85], Liu et al. distinguish the possible downstream analysis in *"overall"*, exploring differences in alpha/beta- diversity and taxonomic composition in a feature table, and *"details analysis"*, identifying biomarkers via comparison (using correlation and/or network analysis, machine learning etc.).

metaGOflow adds to a list of similar approaches such as nf-core/mag [86], metaWRAP [22], MG-RAST [32], JGI-IMG [34], bioBakery 3 (MetaPhlan 3) [23]. metaGOflow highlights the potential that modern workflow managers and containerization technologies support for building workflows upon workflows. Regarding raw data deriving from GOs, metaGOflow facilitates data generation , and, subsequently, interpretation of times-series biodiversity data, thus granting valuable insights to the scientific community and building a solid foundation for long-term sustainable and high-value data outputs. Long-term sustainability is assured by the FAIRness of the outputs and the strategic support of the EMBRC-ERIC infrastructure. Moreover, even if it was initially developed to address the specific needs of a GO project such as EMO BON, metaGOflow is overall a user-friendly flexible workflow that can be broadly used for one-sample-at-a-time analysis of shotgun metagenomics data.

Availability of source code and requirements

- Project name: metaGOflow: A workflow for marine Genomic Observatories data analysis
- Project home page: https://github.com/emo-bon/MetaGOflow
- Manual page: https://metagoflow.readthedocs.io
- WorkflowHub: https://workflowhub.eu/workflows/384
- RRID: SCR_023674
- biotools id: metagoflow
- Operating system(s): Unix
- Programming language: Common Workflow Language (CWL)
- Other requirements: Docker or Singularity engines. Node.js is required in cases where Docker is not available.
- License: Apache License 2.0. For third-party components separate licenses apply. Any restrictions to use by non-academics: licence needed.

Availability of supporting data and materials

Snapshots of our code and other data further supporting this work are openly available in the GigaScience repository, GigaDB 102443 [87]. All the raw sequence files of this study are available at ENA [43]:

- EMO BON super study accession number PRJEB51688 [88]
- EMO BON marine sediment sample: run accession number ERS14961254 [89], study accession number PRJEB51652 [90]
- EMO BON water column sample: run accssion number ERS14961281 [91] study accession number PRJEB51664 [92]
- Tara Oceans sample: run accession number ERR599171 [93], study accession number PRJEB402 [94]

Declarations

List of abbreviations

- CDS: Coding Sequences
- CWL: Common Workflow Language
- EMBRC: European Marine Biological Resource Centre
- EMO BON: European Marine Omics Biodiversity Observation Network
- ENA: European Nucleotide Archive
- GO terms: Gene Ontology terms
- GOs: Genomic Observatories
- HPC: High Performance Computing
- LSU: Large Sub Unit
- OSD: Ocean Sampling Day
- RO-Crate: Research Object Crate
- SSU: Small Sub Unit

Ethical Approval

Not applicable.

Consent for publication

Not applicable.

Competing Interests

M.B., L.R. and R.D.F. are members of the MGnify group that is part of the ELIXIR infrastructure [95]. The authors declare that they have no other competing interests.

Funding

This project has received funding from the European Union's Horizon 2020 research and innovation programme under grant agreement No 824087, under the 1st EOSC-Life Digital Life Sciences Open Call (Project ID 14325) and by the European Marine Biological Resource Centre - European Research Infrastructure Consortium (EMBRC-ERIC), which is part of the European Strategy Forum on Research Infrastructures (ESFRI). M.B. and L.R. were supported by EBML core funds.

Author's Contributions

Conceptualization: C.J.C., R.D.F., C.P.; Project Administration: C.P., A.P., H.Z.; Investigation: H.Z., M.B., S.N., G.D.M., J.M.; Formal Analysis: H.Z.; Software: H.Z., M.B., S.N., J.M., C.J.C.; Methodology: H.Z., S.N., K.E., E.C.; Validation: H.Z., C.P., I.S.; Data Curation: I.S., K.E., C.P., H.Z.; Resources: I.S., R.D.F., L.R., C.J.C., E.P.; Funding Acquisition: C.P., G.K., C.J.C., H.Z., R.D.F.; Writing - Original Draft Preparation: H.Z., C.P.; Writing - Review & Editing: all; Visualization: H.Z.

Acknowledgements

This research was supported in part through computational resources provided by IMBBC (Institute of Marine Biology, Biotechnology and Aquaculture) of the HCMR (Hellenic Centre for Marine Research). Funding for establishing the IMBBC HPC has been received by the MARBIGEN (EU Regpot) project, LifeWatchGreece RI and the CMBR (Centre for the study and sustainable exploitation of Marine Biological Resources) RI. This study received Portuguese national funds from FCT - Foundation for Science and Technology through project UIDB/04326/2020, UIDP/04326/2020 and LA/P/0101/2020, and from the operational programmes CRESC Algarve 2020 and COMPETE 2020 through projects EMBRC.PT ALG-01-0145-FEDER-022121 and BIODATA.PT ALG-01-0145-FEDER-022231 to C.J.C and G.D.M. This work received Computational Time to HPC infrastructures and scientific and technical support from the high-level support team at NCC-Greece. The financial support from the EuroHPC-JU Project 101101903—EuroCC 2 project of the European Commission is acknowledged. Parts of the runs were performed on the MeluXina machine within the project with ID: EHPC-DEV-2022D10-062. The acquisition and operation of the EuroHPC supercomputer is funded jointly by the EuroHPC Joint Undertaking, through the European Union's Connecting Europe Facility and the Horizon 2020 research and innovation programme, as well as the Grand Duché du Luxembourg.

References

1. Louca S, Parfrey LW, Doebeli M. Decoupling function and taxonomy in the global ocean microbiome. Science 2016;353(6305):1272–1277.
2. Doney SC, Ruckelshaus M, Emmett Duffy J, Barry JP, Chan F, English CA, et al. Climate change impacts on marine ecosystems. Annual review of marine science 2012;4:11–37.
3. Chen J, McIlroy SE, Archana A, Baker DM, Panagiotou G. A pollution gradient contributes to the taxonomic, functional, and resistome diversity of microbial communities in marine sediments. Microbiome 2019;7(1):1–12.
4. Caruso G, La Ferla R, Azzaro M, Zoppini A, Marino G, Petochi T, et al. Microbial assemblages for environmental quality assessment: knowledge, gaps and usefulness in the European Marine Strategy Framework Directive. Critical reviews in microbiology 2016;42(6):883–904.
5. Caruso G, Azzaro M, Caroppo C, Decembrini F, Monticelli LS, Leonardi M, et al. Microbial community and its potential as descriptor of environmental status. ICES Journal of Marine Science 2016;73(9):2174–2177.
6. Liu X, Ashforth E, Ren B, Song F, Dai H, Liu M, et al. Bioprospecting microbial natural product libraries from the marine environment for drug discovery. The Journal of Antibiotics 2010;63(8):415–422.
7. Glasl B, Webster NS, Bourne DG. Microbial indicators as a diagnostic tool for assessing water quality and climate stress in coral reef ecosystems. Marine Biology 2017;164(4):1–18.
8. Kopf A, Bicak M, Kottmann R, Schnetzer J, Kostadinov I, Lehmann K, et al. The ocean sampling day consortium. Gigascience 2015;4(1):1–5.
9. Duarte CM. Seafaring in the 21st century: the Malaspina 2010 circumnavigation expedition. Limnology and Oceanography Bulletin 2015;.
10. Sunagawa S, Acinas SG, Bork P, Bowler C, Eveillard D, Gorsky

G, et al. Tara Oceans: towards global ocean ecosystems biology. Nature Reviews Microbiology 2020;18(8):428–445.
11. Zayed AA, Wainaina JM, Dominguez-Huerta G, Pelletier E, Guo J, Mohssen M, et al. Cryptic and abundant marine viruses at the evolutionary origins of Earth's RNA virome. Science 2022;376(6589):156–162.
12. Sunagawa S, Coelho LP, Chaffron S, Kultima JR, Labadie K, Salazar G, et al. Structure and function of the global ocean microbiome. Science 2015;348(6237):1261359.
13. Yelton AP, Acinas SG, Sunagawa S, Bork P, Pedrós-Alió C, Chisholm SW. Global genetic capacity for mixotrophy in marine picocyanobacteria. The ISME journal 2016;10(12):2946–2957.
14. Santi I, Beluche O, Beraud M, Buttigieg P, Casotti R, Cox C, et al. European marine omics biodiversity observation network: a strategic outline for the implementation of omics approaches in ocean observation. Frontiers in Marine Science 2023;10:1118120.
15. Advancing ocean science | EMBRC;. https://www.embrc.eu/.
16. Buck M, Hamilton C. The Nagoya Protocol on access to genetic resources and the fair and equitable sharing of benefits arising from their utilization to the Convention on Biological Diversity. Review of European Community & International Environmental Law 2011;20(1):47–61.
17. GSC, Genomic Standards Consortium;. https://genomicsstandardsconsortium.github.io/gensc.github.io//.
18. Kottmann R, Gray T, Murphy S, Kagan L, Kravitz S, Lombardot T, et al. A standard MIGS/MIMS compliant XML Schema: toward the development of the Genomic Contextual Data Markup Language (GCDML). Omics a journal of integrative biology 2008;12(2):115–121.
19. Samuel RM, Meyer R, Buttigieg PL, Davies N, Jeffery NW, Meyer C, et al. Toward a Global Public Repository of Community Protocols to Encourage Best Practices in Biomolecular Ocean Observing and Research. Frontiers in Marine Science 2021;p. 1488.
20. Decade O, Better Biomolecular Ocean Practices; 2022. https://oceandecade.org/actions/better-biomolecular-ocean-practices/.
21. Tamames J, Cobo-Simón M, Puente-Sánchez F. Assessing the performance of different approaches for functional and taxonomic annotation of metagenomes. BMC genomics 2019;20(1):1–16.
22. Uritskiy GV, DiRuggiero J, Taylor J. MetaWRAP—a flexible pipeline for genome-resolved metagenomic data analysis. Microbiome 2018;6(1):1–13.
23. Beghini F, McIver LJ, Blanco-Míguez A, Dubois L, Asnicar F, Maharjan S, et al. Integrating taxonomic, functional, and strain-level profiling of diverse microbial communities with bioBakery 3. Elife 2021;10:e65088.
24. Ewels PA, Peltzer A, Fillinger S, Patel H, Alneberg J, Wilm A, et al. The nf-core framework for community-curated bioinformatics pipelines. Nature biotechnology 2020;38(3):276–278.
25. Straub D, Blackwell N, Langarica-Fuentes A, Peltzer A, Nahnsen S, Kleindienst S. Interpretations of environmental microbial community studies are biased by the selected 16S rRNA (gene) amplicon sequencing pipeline. Frontiers in Microbiology 2020;11:550420.
26. Merkel D. Docker: lightweight linux containers for consistent development and deployment. Linux journal 2014;2014(239):2.
27. Kurtzer GM, Sochat V, Bauer MW. Singularity: Scientific containers for mobility of compute. PloS one 2017;12(5):e0177459.
28. Di Tommaso P, Chatzou M, Floden EW, Barja PP, Palumbo E, Notredame C. Nextflow enables reproducible computational workflows. Nature biotechnology 2017;35(4):316–319.
29. Mölder F, Jablonski K, Letcher B, Hall M, Tomkins-Tinch C, Sochat V, et al. Sustainable data analysis with Snakemake [version 1; peer review: 1 approved, 1 approved with reservations]. F1000Research 2021;10(33).
30. Zafeiropoulos H, Gioti A, Ninidakis S, Potirakis A, Paragkamian S, Angelova N, et al. 0s and 1s in marine molecular research: a regional HPC perspective. GigaScience 2021;10(8):giab053.
31. ATLAS C, Yamamoto S, Shapiro M, Virzi J, Werner M, Venturi M, et al. The simulation principle and performance of the ATLAS fast calorimeter simulation FastCaloSim. ATL-COM-PHYS-2010-838; 2010.
32. Keegan KP, Glass EM, Meyer F. MG-RAST, a metagenomics service for analysis of microbial community structure and function. In: Microbial environmental genomics (MEG) Springer; 2016.p. 207–233.
33. Mitchell AL, Almeida A, Beracochea M, Boland M, Burgin J, Cochrane G, et al. MGnify: the microbiome analysis resource in 2020. Nucleic acids research 2020;48(D1):D570–D578.
34. Chen IMA, Chu K, Palaniappan K, Pillay M, Ratner A, Huang J, et al. IMG/M v. 5.0: an integrated data management and comparative analysis system for microbial genomes and microbiomes. Nucleic acids research 2019;47(D1):D666–D677.
35. Meyer F, Fritz A, Deng ZL, Koslicki D, Lesker TR, Gurevich A, et al. Critical assessment of metagenome interpretation: the second round of challenges. Nature methods 2022;19(4):429–440.
36. Soiland-Reyes S, Sefton P, Crosas M, Castro LJ, Coppens F, Fernández JM, et al. Packaging research artefacts with RO-Crate. Data Science 2021;5(2):1–42.
37. Wilkinson MD, Dumontier M, Aalbersberg IJ, Appleton G, Axton M, Baak A, et al. The FAIR Guiding Principles for scientific data management and stewardship. Scientific data 2016;3(1):1–9.
38. Wei Q, Khan IK, Ding Z, Yerneni S, Kihara D. NaviGO: interactive tool for visualization and functional similarity and coherence analysis with gene ontology. Bmc Bioinformatics 2017;18(1):1–13.
39. Amstutz P, Crusoe MR, Tijanić N, Chapman B, Chilton J, Heuer M, et al., Common workflow language, v1. 0. figshare; 2016.
40. Language (CWL) CW, Home;. https://www.commonwl.org/.
41. pipeline-v5. MGnify; 2023. https://github.com/EBI-Metagenomics/pipeline-v5, original-date: 2019-05-24T11:11:51Z.
42. Release v.1.0.1: Merge pull request #37 from emo-bon/fix-bugs · emo-bon/MetaGOflow;. https://github.com/emo-bon/MetaGOflow/releases/tag/v.1.0.1.
43. Burgin J, Ahamed A, Cummins C, Devraj R, Gueye K, Gupta D, et al. The European Nucleotide Archive in 2022. Nucleic Acids Research 2023;51(D1):D121–D125.
44. Microbiome Informatics ENA fetch tool. MGnify; 2022. https://github.com/EBI-Metagenomics/fetch_tool, original-date: 2018-09-06T15:38:50Z.
45. metaGOflow: A workflow for marine Genomic Observatories' data analysis. EMO BON; 2023. https://github.com/emo-bon/MetaGOflow, original-date: 2021-10-15T13:40:02Z.
46. metagoflow GitHub wiki page;. https://github.com/emo-bon/MetaGOflow/wiki/Home.
47. Welcome to metaGOflow's documentation! — metaGOflow 0.1 documentation;. https://metagoflow.readthedocs.io/en/latest/.
48. Front Page; 2023. https://www.luxprovide.lu/.
49. Chen S, Zhou Y, Chen Y, Gu J. fastp: an ultra-fast all-in-one FASTQ preprocessor. Bioinformatics 2018;34(17):i884–i890.
50. Nawrocki EP, Eddy SR. Infernal 1.1: 100-fold faster RNA homology searches. Bioinformatics 2013;29(22):2933–2935.
51. Eddy Lab: Home;. http://eddylab.org/.
52. Matias Rodrigues JF, Schmidt TS, Tackmann J, von Mering C. MAPseq: highly efficient k-mer search with confidence estimates, for rRNA sequence analysis. Bioinformatics 2017;33(23):3808–3810.
53. Milanese A, Mende DR, Paoli L, Salazar G, Ruscheweyh HJ, Cuenca M, et al. Microbial abundance, activity and popula-

tion genomic profiling with mOTUs2. Nature communications 2019;10(1):1–11.
54. Ondov BD, Bergman NH, Phillippy AM. Interactive metagenomic visualization in a Web browser. BMC bioinformatics 2011;12(1):1–10.
55. Vollmers J, Wiegand S, Kaster AK. Comparing and evaluating metagenome assembly tools from a microbiologist's perspective-not only size matters! PloS one 2017;12(1):e0169662.
56. Li D, Liu CM, Luo R, Sadakane K, Lam TW. MEGAHIT: an ultra-fast single-node solution for large and complex metagenomics assembly via succinct de Bruijn graph. Bioinformatics 2015;31(10):1674–1676.
57. Nurk S, Meleshko D, Korobeynikov A, Pevzner PA. metaSPAdes: a new versatile metagenomic assembler. Genome Research 2017;27(5):824–834. http://genome.cshlp.org/content/27/5/824.abstract.
58. Rho M, Tang H, Ye Y. FragGeneScan: predicting genes in short and error-prone reads. Nucleic acids research 2010;38(20):e191–e191.
59. Jones P, Binns D, Chang HY, Fraser M, Li W, McAnulla C, et al. InterProScan 5: genome-scale protein function classification. Bioinformatics 2014;30(9):1236–1240.
60. Mitchell AL, Attwood TK, Babbitt PC, Blum M, Bork P, Bridge A, et al. InterPro in 2019: improving coverage, classification and access to protein sequence annotations. Nucleic acids research 2019;47(D1):D351–D360.
61. El-Gebali S, Mistry J, Bateman A, Eddy SR, Luciani A, Potter SC, et al. The Pfam protein families database in 2019. Nucleic acids research 2019;47(D1):D427–D432.
62. Haft DH, Selengut JD, Richter RA, Harkins D, Basu MK, Beck E. TIGRFAMs and genome properties in 2013. Nucleic acids research 2012;41(D1):D387–D395.
63. Sigrist CJ, De Castro E, Cerutti L, Cuche BA, Hulo N, Bridge A, et al. New and continuing developments at PROSITE. Nucleic acids research 2012;41(D1):D344–D347.
64. Ashburner M, Ball CA, Blake JA, Botstein D, Butler H, Cherry JM, et al. Gene ontology: tool for the unification of biology. Nature genetics 2000;25(1):25–29.
65. Huerta-Cepas J, Szklarczyk D, Heller D, Hernández-Plaza A, Forslund SK, Cook H, et al. eggNOG 5.0: a hierarchical, functionally and phylogenetically annotated orthology resource based on 5090 organisms and 2502 viruses. Nucleic acids research 2019;47(D1):D309–D314.
66. Cantalapiedra CP, Hernández-Plaza A, Letunic I, Bork P, Huerta-Cepas J. eggNOG-mapper v2: functional annotation, orthology assignments, and domain prediction at the metagenomic scale. Molecular biology and evolution 2021;38(12):5825–5829.
67. Eddy SR. Accelerated profile HMM searches. PLoS computational biology 2011;7(10):e1002195.
68. Aramaki T, Blanc-Mathieu R, Endo H, Ohkubo K, Kanehisa M, Goto S, et al. KofamKOALA: KEGG Ortholog assignment based on profile HMM and adaptive score threshold. Bioinformatics 2020;36(7):2251–2252.
69. Kanehisa M, Sato Y, Kawashima M, Furumichi M, Tanabe M. KEGG as a reference resource for gene and protein annotation. Nucleic acids research 2016;44(D1):D457–D462.
70. Soiland-Reyes S, Sefton P, Crosas M, Castro LJ, Coppens F, Fernández JM, et al. Packaging research artefacts with RO-Crate. Data Science 2022;5(2):97–138.
71. De Geest P, Droesbeke B, Eguinoa I, Gaignard A, Huber S, Leo S, et al., ro-crate-py. Zenodo; 2022. https://doi.org/10.5281/zenodo.6594974, cite as.
72. Santi I, Casotti R, Comtet T, Cunliffe M, Koulouri PY, Macheriotou L, et al. European Marine Omics Biodiversity Observation Network (EMO BON) Handbook (Version 1.0). EMBRC-ERIC; 2021.
73. Pesant S, Not F, Picheral M, Kandels-Lewis S, Le Bescot N, Gorsky G, et al. Open science resources for the discovery and analysis of Tara Oceans data. Scientific data 2015;2(1):1–16.
74. Zafeiropoulos H, Beracochea M, Ninidakis S, Exter K, Moro GD, Potirakis A, et al., metaGOflow: a workflow for the analysis of marine Genomic Observatories shotgun metagenomics data - use case. Zenodo; 2023. https://doi.org/10.5281/zenodo.8046421.
75. use cases;. https://data.emobon.embrc.eu/MetaGOflow/.
76. Quince C, Walker AW, Simpson JT, Loman NJ, Segata N. Shotgun metagenomics, from sampling to analysis. Nature biotechnology 2017;35(9):833–844.
77. Faust K, Sathirapongsasuti JF, Izard J, Segata N, Gevers D, Raes J, et al. Microbial co-occurrence relationships in the human microbiome. PLoS computational biology 2012;8(7):e1002606.
78. Friedman J, Alm EJ. Inferring correlation networks from genomic survey data. PLoS computational biology 2012;8(9):e1002687.
79. Bharti R, Grimm DG. Current challenges and best-practice protocols for microbiome analysis. Briefings in bioinformatics 2021;22(1):178–193.
80. Reimer LC, Sardà Carbasse J, Koblitz J, Ebeling C, Podstawka A, Overmann J. Bac Dive in 2022: the knowledge base for standardized bacterial and archaeal data. Nucleic Acids Research 2022;50(D1):D741–D746.
81. Zafeiropoulos H, Paragkamian S, Ninidakis S, Pavlopoulos GA, Jensen LJ, Pafilis E. PREGO: a literature and data-mining resource to associate microorganisms, biological processes, and environment types. Microorganisms 2022;10(2):293.
82. contributors TUoMUaB, Pitfalls and limitations · BioExcel Best Practice Guide: Creating workflows with Common Workflow Language; 2021. http://docs.bioexcel.eu/cwl-best-practice-guide/limitations.html.
83. Vivian J, Rao AA, Nothaft FA, Ketchum C, Armstrong J, Novak A, et al. Toil enables reproducible, open source, big biomedical data analyses. Nature biotechnology 2017;35(4):314–316.
84. Sharpton TJ. An introduction to the analysis of shotgun metagenomic data. Frontiers in plant science 2014;5:209.
85. Liu YX, Qin Y, Chen T, Lu M, Qian X, Guo X, et al. A practical guide to amplicon and metagenomic analysis of microbiome data. Protein & cell 2021;12(5):315–330.
86. Krakau S, Straub D, Gourlé H, Gabernet G, Nahnsen S. nf-core/mag: a best-practice pipeline for metagenome hybrid assembly and binning. NAR Genomics and Bioinformatics 2022;4(1):lqac007.
87. Zafeiropoulos H, Beracochea M, Ninidakis S, Exter K, Potirakis A, De Moro G, et al., Supporting data for "metaGOflow: a workflow for the analysis of marine Genomic Observatories shotgun metagenomics data. GigaScience Database; 2023. http://dx.doi.org/10.5524/102443.
88. EMO BON ENA super study record;.http://www.ebi.ac.uk/ena/data/view/PRJEB51688, accessed: 2023-09-01.
89. EMO BON sediment sample ENA run record;.http://www.ebi.ac.uk/ena/data/view/ERS14961254, accessed: 2023-09-01.
90. ENA study record for EMO BON Observatory Station BPNS (VLIZ, UGhent, RBINS, KULeuven) samples;.http://www.ebi.ac.uk/ena/data/view/PRJEB51652, accessed: 2023-09-01.
91. EMO BON water column sample ENA run record;.http://www.ebi.ac.uk/ena/data/view/ERS14961281, accessed: 2023-09-01.
92. ENA study record for EMO BON Observatory Station VB (IMEV) samples;.http://www.ebi.ac.uk/ena/data/view/PRJEB51664, accessed: 2023-09-01.
93. TARA OCEAN sample ENA run record;.http://www.ebi.ac.uk/ena/data/view/ERR599171, accessed: 2023-09-01.
94. TARA OCEAN ENA study record;.http://www.ebi.ac.uk/ena/data/view/PRJEB402, accessed: 2023-09-01.
95. ELIXIR;. https://elixir-europe.org/.

Supplementary Table 1

Click here to access/download
Supplementary Material
metaGOflow_Supplementary-Table-1.docx
