# Supplementary material for: metaGOflow: a workflow for the analysis of marine Genomic Observatories shotgun metagenomics data
Source: Gigascience. 2023 Oct 18;12:giad078. doi: 10.1093/gigascience/giad078 (PMC10583283; doi:10.1093/gigascience/giad078)
Supplement: giad078_Supplemental_File [file giad078_supplemental_file.docx]

Supplementary Table 1: metaGOflow results for the two gut samples. ERR4765907: fish gut sample; SRR9654976: human gut sample.

| **product** | **ERR4765907** | **SRR9654976** |
| --- | --- | --- |
| total reads (M) | 4.3 | 12.7 |
| filtered reads (M) | 1.44 | 7.8 |
| SSU | 71 | 268 |
| LSU | 75 | 308 |
| contigs | 2476 | 75624 |
| Reads with predicted CDS (M) | 1.3 | 7.7 |
| Pred. CDS* with IPS match (M) | 0.2 | 2.2 |
| Pred. CDS with GO match (M) | 0.14 | 1.26 |
| Pred. CDS with Pfam match (M) | 0.19 | 2.01 |
| Pred. CDS with KO match (M) | 0.01 | 0.22 |

M: millions, *CDS: Coding Sequences
