# Supplementary material for: metaGOflow: a workflow for the analysis of marine Genomic Observatories shotgun metagenomics data
Source: Gigascience. 2023 Oct 18;12:giad078. doi: 10.1093/gigascience/giad078 (PMC10583283; doi:10.1093/gigascience/giad078)
Supplement: giad078_Response_to_Reviewer_Comments_Original_Submission [file giad078_response_to_reviewer_comments_original_submission.pdf]

Author's Response To Reviewer Comments

Our point-by-point response has been uploaded as a separate file.
