# Supplementary material for: metaGOflow: a workflow for the analysis of marine Genomic Observatories shotgun metagenomics data
Source: Gigascience. 2023 Oct 18;12:giad078. doi: 10.1093/gigascience/giad078 (PMC10583283; doi:10.1093/gigascience/giad078)
Supplement: giad078_Response_to_Reviewer_Comments_Revision_3 [file giad078_response_to_reviewer_comments_revision_3.pdf]

Author's Response To Reviewer Comments

- URLs need to be moved to the references
Done as suggested.

- The GigaDB DOI needs to be cited under Data Availability and added to the References
Done as suggested.

- RRID number mentioned under "overview" at first mention of metaGOflow.
Done as suggested.

- Please also check any preprint in the References. If they are officially published, please update the citation accordingly.
Done as suggested.

The latex files have also been included.
