# Supplementary material for: metaGOflow: a workflow for the analysis of marine Genomic Observatories shotgun metagenomics data
Source: Gigascience. 2023 Oct 18;12:giad078. doi: 10.1093/gigascience/giad078 (PMC10583283; doi:10.1093/gigascience/giad078)
Supplement: giad078_Reviewer_1_Report_Original_Submission — Artem Barski -- 6/8/2023 Reviewed [file giad078_reviewer_1_report_original_submission.pdf]

Reviewer Report

Title: metaGOflow: a workflow for the analysis of marine Genomic Observatories shotgun metagenomics data

Version: Original Submission **Date:** 6/8/2023

Reviewer name: Artem Barski

Reviewer Comments to Author:

The submitted manuscript describes a bioinformatics workflow called metaGOflow. It is billed as a user-friendly flexible workflow that can be broadly used for one-sample-at-a-time analysis of shotgun metagenomics data. The pipeline is essentially a wrapper of existing metagenomic sequencing data tools packaged into a portable workflow and uses data standards such as RO-crates for organizing sample outputs. The resulting workflow is supposed to be portable and the analysis results comparable when it is run at different research centers. This is an important development: making pipelines portable and usable by others is important for the field. Unfortunately, the current implementation of metaGOflow is not as portable as onewe would have liked. There is still significant overhead of additional dependencies required to be set up on the server running the workflow components, instead of having all but the CWL runner dockerized for maximum portability. The size of a database is another hurdle, however the fact it is all packaged into a single "installation" script helps ease its use. Scientific description of inputs, results and further use of the data is not included in the manuscript. Overall, this manuscript is of interest. and Iif the pipeline is properly implemented it and the paper will be of great use for the scientific community. However, the following issues need to be addressed for publication:There is still too much overhead with respect to deployment for this workflow to be considered portable, so we suggest the following adjustments to increase ease of use towards greater adoption of the workflow:Remove any root access requirements for installationDockerize all dependencies used by the tools so that all a user needs to install to run the workflow is the workflow runner (CWL) and docker. Currently, installation of the following tools is required:conda and a supporting environmentcwlref-runnercwltool[all]rdflib-jsonldrocratepyyamlResults from shotgun metagenomic samples from various environments should be added to the results tables in order to cover a broader range of abundance profiles.Discussion of applicability of the pipeline to other non-marine data types would be useful. For example, can it be used for human microbiome data or soil data? Does anything (e.g. the database) need to be changed for this?Need to have at least some comparison with the alternative pipelines for processing this data on both speed, logic, and scientific outputs.A brief discussion of input data from a molecular biology standpoint will be useful: how are the libraries for sequencing prepared? What kit? Does it matter?The description of outputs from the scientific standpoint is absent in the manuscript. It is not clear what results are produced, how to tell if the quality of data is good, what files are produced by the pipeline, and how to interpret the results. This is also not present in the wiki: just including the directory structure of the outputs without any description is not conducive to the use of the pipeline by anybody other than the developers.It would be nice to produce some kind of standardized report with key results e.g. in the form of html that can be interpreted by biologists.It is assumed that processing individual

samples with metaGOflow is supposed to be followed by differential analysis. No script or even discussion for this is provided but are needed to make the pipeline useful.Minor comments:Comparison of results is not addressed and is perhaps out of scope for this publication, however we suggest commenting on how metaGOflow output can interface with potential downstream applications such as differential abundance analyses.The following remark in the Introduction is perhaps a bit overblown. Analyzing 540 microbiome samples should not exceed the computing capacity of a research institute."The computing requirements for the analysis of the EMO BON data exceed the computing capacity that a single research institute or even a regional High Performance Computing (HPC) system can support using the available workflows."Spelling: Implementation > Overview > second paragraph > "metaGOlfow"Spelling: Implementation > Step 5: Functional annotation of the reads > "ProSite patterns and prorfiles"As part of your review, please consider the following:Does the manuscript adhere to the journal's guidelines on minimum standards of reporting?Key questions we ask reviewers to carefully check are:1) Are the methods appropriate to the aims of the study, are they well described, and are necessary controls included?The aim of the study is to present a standardized workflow for shotgun metagenomic data, which is indeed well described for single samples. However, the only sample results presented in the study are a comparison of computational resources at various steps in the workflow. The tools used in the workflow are simply cited, and their accuracy not explicitly assessed or even addressed, though that is likely out of this study's scope. It would be beneficial to see the results produced by shotgun metagenomic samples from various environments that cover a range of abundance profiles (https://www.sigmaaldrich.com/US/en/technical-documents/technical-article/genomics/next-gen-sequencing/metagenomic-research-into-microbiome-communities) in order to better assess metaGOflow for applications outside of marine environments.2) Are the conclusions adequately supported by the data shown?Yes, their conclusion that their workflow is well suited to support EMO BOM environmental studies is supported by the results in Table 1, assuming that these samples are representative. Additionally, they rightly claim that modern workflow managers are a useful tool for large genomic studies that require rigorous standardization of analysis protocols.3) Please indicate the quality of language in the manuscript. Does it require a heavy editing for language and clarity?The quality of language in the manuscript does not require heavy editing, and is suitable for publication.4) Are you able to assess all statistics in the manuscript, including the appropriateness of statistical tests used?There are no statistics used in the manuscript.

Level of Interest

Please indicate how interesting you found the manuscript: Choose an item.

Quality of Written English

Please indicate the quality of language in the manuscript: Choose an item.

Declaration of Competing Interests

Please complete a declaration of competing interests, considering the following questions:

- Have you in the past five years received reimbursements, fees, funding, or salary from an organisation that may in any way gain or lose financially from the publication of this manuscript, either now or in the future?
- Do you hold any stocks or shares in an organisation that may in any way gain or lose financially from the publication of this manuscript, either now or in the future?
- Do you hold or are you currently applying for any patents relating to the content of the manuscript?
- Have you received reimbursements, fees, funding, or salary from an organization that holds or has applied for patents relating to the content of the manuscript?
- Do you have any other financial competing interests?
- Do you have any non-financial competing interests in relation to this paper?

If you can answer no to all of the above, write 'I declare that I have no competing interests' below. If your reply is yes to any, please give details below.

AB is a co-founder and RP is an employee of Datirium, LLC which is developing SciDAP, a data analysis platform that uses CWL pipelines. Datirium may benefit if the pipeline described here follows standards, is easy to implement in SciDAP and produces clear and useful results.

I agree to the open peer review policy of the journal. I understand that my name will be included on my report to the authors and, if the manuscript is accepted for publication, my named report including any attachments I upload will be posted on the website along with the authors' responses. I agree for my report to be made available under an Open Access Creative Commons CC-BY license (http://creativecommons.org/licenses/by/4.0/). I understand that any comments which I do not wish to be included in my named report can be included as confidential comments to the editors, which will not be published.

Choose an item.

To further support our reviewers, we have joined with Publons, where you can gain additional credit to further highlight your hard work (see: https://publons.com/journal/530/gigascience). On publication of this paper, your review will be automatically added to Publons, you can then choose whether or not to claim your Publons credit. I understand this statement.

Yes Choose an item.
