# Supplementary material for: metaGOflow: a workflow for the analysis of marine Genomic Observatories shotgun metagenomics data
Source: Gigascience. 2023 Oct 18;12:giad078. doi: 10.1093/gigascience/giad078 (PMC10583283; doi:10.1093/gigascience/giad078)
Supplement: giad078_Reviewer_1_Report_Revision_1 — Artem Barski -- 7/17/2023 Reviewed [file giad078_reviewer_1_report_revision_1.pdf]

Reviewer Report

Title: metaGOflow: a workflow for the analysis of marine Genomic Observatories shotgun metagenomics data

Version: Revision 1 **Date:** 7/17/2023

Reviewer name: Artem Barski

Reviewer Comments to Author:

While, in this reviewer's opinion, the pipeline is a very useful contribution to the field, the authors are shooting themselves in the foot by not providing sufficient explanations in either manuscript or manual/wiki to make it usable by the community. The revised manuscript does show improvement in several areas, however most of my concerns were not addressed. As such, I cannot endorse the publication at this stage but will be happy to re-review one more time if the authors decide to address the comments and re-submit.In general, the authors need to decide whether MetaGOflow is an internal EMO BON routine or is widely applicable. If it is an internal routine, then it does not need to be published in GigaScience and can be reported in the EMO BON handbook. If the authors still think that MetaGOflow is widely applicable and of interest to the wider audience of GigaScience, they need to provide a much better scientific description of their pipeline, inputs and scientific outputs/applications than what is currently presented in either the paper or the manual/wiki. Not having this information will make it extremely difficult for other researchers to use the pipeline and to compare it with other available tools. Current paper and wiki/manual seem to be written for people who routinely use the pipeline within the EMO BON project, not for the wider audience.Please try to imagine your reader as a researcher who sent the sample for metagenomic sequencing and received the fastq files that need to be analyzed. Such reader would want to go through your manuscript and wiki and quickly understand:Can I use metaGOflow with my read data?Do I need to adjust my data and if so, how?What results will I get?How can I use these results?What scientific conclusions will I be able to make?How are these results different from whatever I can obtain from other pipelines?The authors argue that in the "manuscript we are not aiming to describe what metagenomics is, or how it's used, or how it's interpreted." The authors may want to reconsider.Many creators of other tools go well out of their way to very plainly explain how their software is used, what it produces, and how to interpret the results using varied examples.Specific questions:Issue 1.3 and 1.9: This is far from sufficient description. The new figure 3 shows the json structure but does not provide any usable information. I understand that the authors are trying to keep the manuscript short, but they can include more information in the manual. The manual needs to include the scientific description of each input and output file and its contents (What are the rows and columns?). How the data are supposed to be interpreted: e.g., are the base quality plots shown in Fig. 1b good or bad?Issue 1.4: Thank you for addressing some of these issues, we understand it can be challenging to create packages that work "out of the box" so to speak that do not require at least some kind of environment setup. However, if the intention of metaGOflow is to be used by "professional systems administrators" of larger computing infrastructure, this should be explicitly stated or at least remove mentions of the software being "easy-to-use".Issue 1.5: The use of

metaGOflow on fish and human gut samples illustrates a wider applicability of the pipeline. I appreciate the inclusion of these results in the manuscript. Regarding the host sequences: would it be possible to include an optional step for their removal? This will make the pipeline easier to use for researchers.Issue 1.7: I appreciate inclusion of the table listing the pipeline steps. In addition, a similar table comparing the scientific results produced by each pipeline needs to be included.Issue 1.8: The readers should not be forced to browse the Handbook to figure out what kind of data can be analyzed with the pipeline. Specifically, the question before the user is whether the pipeline is dependent on what adapters are used for library construction.Issue 1.10. The authors mention that some of the data can be visualized with tools like "ShinyGO, DICE, KOBAS-i and more. Please provide brief instructions for doing this together with the scientific description of results as discussed in 1.3.Issue 1.11 The explanation of the further use of data included in the latest version is insufficient. How are the outputs of the pipeline used at EMO BON? The authors need to significantly expand on this topic in the manual/wiki if not in the manuscript itself. Specifically, this discussion should include what tools can be used (e.g. LefSE), what files can be used as input (i.e. extracted from the RO-crate), do they need to be modified, example scripts.

Level of Interest

Please indicate how interesting you found the manuscript: Choose an item.

Quality of Written English

Please indicate the quality of language in the manuscript: Choose an item.

Declaration of Competing Interests

Please complete a declaration of competing interests, considering the following questions:

- Have you in the past five years received reimbursements, fees, funding, or salary from an organisation that may in any way gain or lose financially from the publication of this manuscript, either now or in the future?
- Do you hold any stocks or shares in an organisation that may in any way gain or lose financially from the publication of this manuscript, either now or in the future?
- Do you hold or are you currently applying for any patents relating to the content of the manuscript?
- Have you received reimbursements, fees, funding, or salary from an organization that holds or has applied for patents relating to the content of the manuscript?
- Do you have any other financial competing interests?
- Do you have any non-financial competing interests in relation to this paper?

If you can answer no to all of the above, write 'I declare that I have no competing interests' below. If your reply is yes to any, please give details below.

I am a co-owner of Datirium, the developer of a data analysis platform, SciDAP that uses CWL-based pipelines. I may benefit from the existence of cWL pipelines such as MetaGOflow.

I agree to the open peer review policy of the journal. I understand that my name will be included on my report to the authors and, if the manuscript is accepted for publication, my named report including any attachments I upload will be posted on the website along with the authors' responses. I agree for my report to be made available under an Open Access Creative Commons CC-BY license (http://creativecommons.org/licenses/by/4.0/). I understand that any comments which I do not wish to be included in my named report can be included as confidential comments to the editors, which will not be published.

Choose an item.

To further support our reviewers, we have joined with Publons, where you can gain additional credit to further highlight your hard work (see: https://publons.com/journal/530/gigascience). On publication of this paper, your review will be automatically added to Publons, you can then choose whether or not to claim your Publons credit. I understand this statement.

Yes Choose an item.
