# Supplementary material for: metaGOflow: a workflow for the analysis of marine Genomic Observatories shotgun metagenomics data
Source: Gigascience. 2023 Oct 18;12:giad078. doi: 10.1093/gigascience/giad078 (PMC10583283; doi:10.1093/gigascience/giad078)
Supplement: giad078_Reviewer_2_Report_Original_Submission — Samuel Lampa, PhD -- 6/10/2023 Reviewed [file giad078_reviewer_2_report_original_submission.pdf]

Reviewer Report

Title: metaGOflow: a workflow for the analysis of marine Genomic Observatories shotgun metagenomics data

Version: Original Submission **Date:** 6/10/2023

Reviewer name: Samuel Lampa, PhD

Reviewer Comments to Author:

The paper reads quite well language-wise, and provides a thorough discussion of the challenges in the field of metagenomics, discusses alternative approaches, and describes the approach taken here, with a suitable level of detail.Main comments============There are two things that in my view need editing though.Firstly, the paper contains a number of sentences that appear very unclear and need editing to make sure statements are clear, concrete and backed up with examples where appropriate.Secondly but closely related is that the discussion of the novel features provided by metaGOflow is quite thin. The novel features are only listed briefly in the Introductions section, and the discussion of what improvements metaGOflow brought is to me very unclear from the discussion.For example, I think sentences like these:"metaGOflow adds on a list of similar approaches such as nf-core/mag [64], metaWRAP [19], MG-RAST [23], JGI-IMG [25],bioBakery 3 (MetaPhlan 3) [20]. metaGOflow highlights the poten-tial that modern workflow managers and containerization technolo-gies support for building workflows upon workflows"... don't say very much at all, and need to be made much more concrete.For example, in what concrete way did workflows and containerization help improve the analysis?The rest of this last paragraph provides more information, but needs to be expanded with more concrete examples.For example in this sentence:"Regarding raw data deriving from GOs, metaGOflow facilitates data generation for the interpretation of biodiversity changes over time [...]"... this sounds OK..."[...] thus granting valuable insights to the scientific community and building a solid foundation for long-term sustainable and high-value data outputs."Here I have questions though. In what way does interpretation over time make data products long-term sustainable?I might be missing something here as a non-expert in metagenomics, but I get the feeling that some of these statements are not connected.Perhaps I'm wrong, but I think at the very least it needs to be made more concrete.Again, as said, I think the paper needs editing to make sure all sentences are clear, concrete and backed up by examples where appropriate.Further comments==============1. The figure legend for Figure 1 is way too short and needs to explain with a few sentences what is seen in the image.2. The CDS abbreviation is not explained in Table 2.3. CDS is missing from the abbreviations list.4. It seems Node.js is a requirement that should be listed, at least as an alternative to Docker/Singularity?Language fixes and typos===================1. Regarding this sentence on page 2:"The computing requirements for the analysis of the EMO BON data exceed the computing capacity that a single research institute or even a regional High Performance Computing (HPC) system can support using the available workflows"It would be fitting with a rough estimate of at least the order of magnitude of computational resources needed here. TBs of RAM? hundreds of CPU hours?2. Typo at the end of page 2 (last paragraph):metaGOlfow -> metaGOflow3. Sentence bug in second paragraph on page 3:"preferably in as

short a period of time possible" -->"preferably in as short a period of time as possible"4. Sentence bug on page 3, 4th paragraph:"metaGOflow data products are packages RO-Crates:" -->"metaGOflow data products are packaged as RO-Crates"5. Sentence bug on page 4:"support for fast sequence retrieval" -->"support fast sequence retrieval"6. Sentence bug on page 5, last paragraph:"metaGOflow adds on a list" -->"metaGOflow adds to a list"

Level of Interest

Please indicate how interesting you found the manuscript: Choose an item.

Quality of Written English

Please indicate the quality of language in the manuscript: Choose an item.

Declaration of Competing Interests

Please complete a declaration of competing interests, considering the following questions:

- Have you in the past five years received reimbursements, fees, funding, or salary from an organisation that may in any way gain or lose financially from the publication of this manuscript, either now or in the future?
- Do you hold any stocks or shares in an organisation that may in any way gain or lose financially from the publication of this manuscript, either now or in the future?
- Do you hold or are you currently applying for any patents relating to the content of the manuscript?
- Have you received reimbursements, fees, funding, or salary from an organization that holds or has applied for patents relating to the content of the manuscript?
- Do you have any other financial competing interests?
- Do you have any non-financial competing interests in relation to this paper?

If you can answer no to all of the above, write 'I declare that I have no competing interests' below. If your reply is yes to any, please give details below.

I declare that I have no competing interests

I agree to the open peer review policy of the journal. I understand that my name will be included on my report to the authors and, if the manuscript is accepted for publication, my named report including any attachments I upload will be posted on the website along with the authors' responses. I agree for my report to be made available under an Open Access Creative Commons CC-BY license (http://creativecommons.org/licenses/by/4.0/). I understand that any comments which I do not wish to be included in my named report can be included as confidential comments to the editors, which will not be published.

Choose an item.

To further support our reviewers, we have joined with Publons, where you can gain additional credit to further highlight your hard work (see: https://publons.com/journal/530/gigascience). On publication of this paper, your review will be automatically added to Publons, you can then choose whether or not to claim your Publons credit. I understand this statement.

Yes Choose an item.
